# Target Class Repurposing Across Membrane Transporter Families Provides Privileged Ligands to Address Specific and Undruggable Pharmacological Targets

**DOI:** 10.1021/acsptsci.5c00430

**Published:** 2026-01-27

**Authors:** Muhammad Rafehi, Franziska Tägl, Nike Sophia Arlt, Maria Neif, Katja Stefan, Wouroud Ismail Al-Khalil, Hauke Busch, Marius Möller, Jörg König, Vigneshwaran Namasivayam, Sven Marcel Stefan

**Affiliations:** † Institute of Clinical Pharmacology, 27177 University Medical Center Göttingen, Robert-Koch-Str. 40, 37075 Göttingen, Germany; ‡ Faculty of Medicine, University of Augsburg and University Hospital of Augsburg, Am Medizincampus 2, 86156 Augsburg, Germany; § Lübeck Institute of Experimental Dermatology (LIED), Medicinal Chemistry and Systems Polypharmacology, 9191 University of Lübeck and University Medical Center Schleswig-Holstein (UKSH), Ratzeburger Allee 160, 23538 Lübeck, Germany; ∥ Department of Pathology, Rikshospitalet, 6305 University of Oslo and Oslo University Hospital, Sognsvannsveien 20, 0372 Oslo, Norway; ⊥ Lübeck Institute of Experimental Dermatology (LIED), Medical Systems Biology, University of Lübeck and University Medical Center Schleswig-Holstein (UKSH), Ratzeburger Allee 160, 23538 Lübeck, Germany; # Institute of Experimental and Clinical Pharmacology and Toxicology, 9171 Friedrich-Alexander-Universität Erlangen-Nürnberg (FAU), 91054 Erlangen, Germany; ∇ FAU NeW - Research Center New Bioactive Compounds, Friedrich-Alexander-Universität Erlangen-Nürnberg (FAU), Nikolaus-Fiebiger-Str. 10, 91058 Erlangen, Germany; ○ Department of Biopharmacy, Medical University of Lublin, Chodzki 4a, 20-093 Lublin, Poland

**Keywords:** ABC transporter, solute carrier, polyspecificity, polypharmacology, undruggability, privileged structure

## Abstract

Altogether, 60–70% of the ATP-binding cassette (ABC) and solute carrier (SLC) transporters can currently not be targeted by drugs, despite their involvement in human diseases. The design of potential drug candidates relies on hit identification and subsequent optimization with regard to selectivity and specificity. However, these workflows ultimately fail if no hit molecules can be found. We pursued a strategy of rational discovery of hit molecules for ‘undruggable’ ABC and SLC transporters based on polypharmacology as an alternative approach in the drug development repertoire. The 42 most polypharmacological ABC transporter modulators were profiled against eight specific (NAT, DAT, and SERT) and polyspecific (OCT1–3, MATE1–2K) SLCs. The general hit rate increased expectedly with the degree of polyspecificity, ranging from 0 to 9.52% (NAT, DAT, SERT) to 19.0–52.4% (OCT1–3, MATE1–2K). Striking was the hit rate for potent drugs, which was highest for the specific transporter SERT (75.0%); additionally, pranlukast (**PRA**) could also be identified as common substrate of NAT, DAT, SERT, and MATE2K. The polypharmacology of drugs correlated with their potency, and a higher degree of polypharmacology against ABCs was reflected in a higher degree of polypharmacology against SLCs. Some compounds mediated between both specific and polyspecific transporters which could be underpinned by the identification of common molecular features (‘privileged structures’). The polypharmacology of selected drugs could be transferred to ABCA1 and Oatp1d1, two transporters for which almost no modulators have been reported before. This strategy provided privileged ligands with high potency at high hit rates to challenge transporter undruggability.

## Graphical abstract

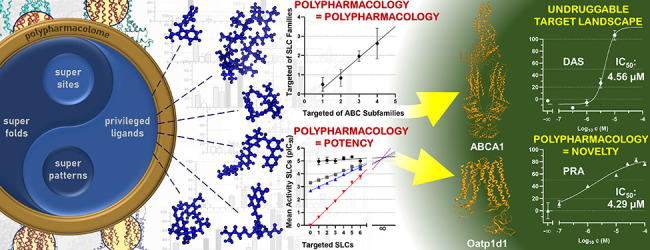

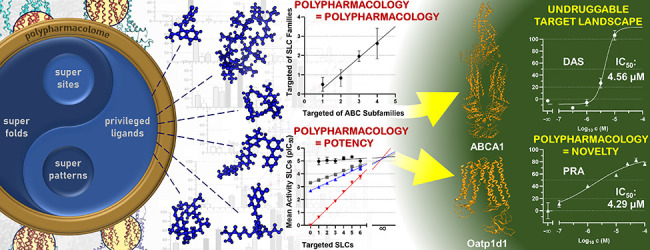

ATP-binding cassette transporters (ABCs) and solute carriers (SLCs) regulate the distribution of solutes across membranes, e.g.*,* amino acids/peptides, hormones/neurotransmitters, ions, bile acids, nucleosides/nucleotides, sugars, and vitamins.^1–3^ Furthermore, they are involved in the absorption, distribution, and elimination of drugs, therefore critically influencing drug pharmacokinetics and efficacy. Many ABCs and SLCs have been identified as important players in both prevalent and rare human diseases, however, their physiological and pathological roles are mostly poorly understood.^2–6^ Chemical probes and drug candidates for these membrane transporters are mostly lacking, and associated diseases can currently not be cured.

In total, 48 functional ABCs and far over 450 SLCs have been identified in the human proteome so far.^5,7^ A recent study suggested the existence of 120 additional SLCs that have not been classified yet.^8^ According to the PubChem database (https://pubchem.ncbi.nlm.nih.gov), 29 ABCs and ≫307 SLCs are undruggable (i.e., as yet no modulators available, and thus currently not chemically/pharmacologically tractable^9^), and a very limited number of ligands (i.e., up to 10) was reported for 7 ABCs and 61 SLCs only.^1,2,10,11^ Figure 1 visualizes the emptiness of the modulator landscapes of ABCs- and SLCs-targeting modulators.

**Figure 1:**
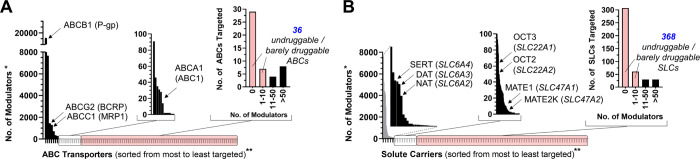
Distribution of the ABC (A) and SLC (B) transporter modulator landscapes; *Data retrieved from PubChem [https://pubchem.ncbi.nlm.nih.gov (accessed March 20–27, 2025); compounds annotated as ‘active’ on the PubChem database have been taken into account; compounds annotated as ‘unspecified’, ‘inconclusive’, and ‘inactive’ have not been considered]; **48 human ABCs and 428 human SLCs were available on PubChem and ‘SLC tables’ (https://slc.bioparadigms.org). (A, B) Left diagrams: Entire landscape; center diagrams: ABCs (A) and SLCs (B) targeted by up to 100 modulators; right diagrams: ABCs (A) and SLCs (B) that are targeted by 0, 1–10, 11–50, and >50 modulators; undruggable (i.e., as yet no modulators available, and thus currently not chemically/pharmacologically tractable) and barely druggable (up to 10 modulators available) ABCs (A) and SLCs (B) are highlighted in pink; black arrows: ABCs analyzed (A) and SLCs assessed (B) within this study.

‘Drugging the undruggable’ is a field of increasingly large interest.^9,12–14^ One strategy to gain modulators for undruggable (i.e., as yet no modulators available, and thus currently not chemically/pharmacologically tractable^9^) or barely druggable (i.e., only very few modulators with unsatisfactory properties available) pharmacological targets is structure-based drug design using resolved protein structures of ABCs and SLCs.^10,15^ However, only very few transporter structures are available so far, particularly considering human orthologs. Artificial intelligence-(AI)-based techniques such as AlphaFold may fill this gap,^16^ however, the prediction accuracy for large and highly flexible proteins, such as ABCs and SLCs, is challenging and the use of AlphaFold therefore disputed within the transporter communities. Another approach involves the use of polypharmacology, i.e., the ability of ligands to address multiple, related or unrelated, pharmacological targets.^17–20^

Proteins of functional and/or phylogenetic distance share structural commonalities (i.e., ‘superfolds’)^17,21–24^ which may form ‘supersites’ ^11,17,21,25–29^ that are addressed by ‘privileged ligands’,^17,29–31^ which, for their part, bear reoccurring chemical partial structures (i.e., ‘privileged structures’ or ‘superpatterns’).^17,19^ The opportunity space spanning superfolds, supersites, privileged ligands, and superpatterns has been referred to by us as the ‘polypharmacolome’ ^17–19^ which forms the molecular basis for the polyspecificity of pharmacological targets (i.e., the ability to bind several ligands) and the polypharmacology of drugs (i.e., the ability to address several pharmacological targets). Pilot studies revealed conserved tyrosine-phenylalanine-serine-threonine binding motifs across ABCs and SLCs,^25,26^ and several drugs have already been suggested as privileged ligands for undruggable membrane transporters.^32^

The discovery of privileged ligands requires comprehensive assessment platforms, including multiple pharmacological targets, which have barely been described in the past.^33,34^ The aim of the present study was the evaluation of diverse drugs against a panel of specific transporters [i.e., the monoamine transporters (MATs) for noradrenaline (NAT, *SLC6A2*), dopamine (DAT, *SLC6A3*), and serotonin (SERT, *SLC6A4*)] and polyspecific transporters [i.e., organic cation transporters 1–3 (OCT1–3; *SLC22A1–3*) as well as the multidrug and toxin extrusion transporters 1 (MATE1; *SLC47A1*) and 2K (MATE2K; *SLC47A2*)]. These particular pharmacological targets were chosen, as they (i) showed different degrees of specificity/polyspecificity; (ii) have been well characterized in the past including well-established expression systems and functional assays; and (iii) were all immediately available in our in-house cell biology. Correlation studies on the relationship between polypharmacology, potency, and chemical composition have been performed, and favorable privileged ligands were assessed against ABCA1 and organic anion transporting polypeptide 1d1 (Oatp1d1), both prototypical model systems for transporter undruggability given the scarce numbers of drugs and reports available.

## Results

### Compilation of a ‘Polypharmacology Compound Library’ Targeting ABC Transporters

In a recent study, we applied a target class repurposing approach to improve the druggability of certain bacterial ABC transporters as promising pharmacological targets against antimicrobial resistance.^35^ This report provided a list of all 280 multitarget (pan-)­ABC transporter modulators that addressed at least 3 ABC transporters, from which only 154 were commercially available. For the present study, we procured over a quarter of these compounds: (i) all compounds addressing at least 5 ABC transporters (25 in total); (ii) three-quarters of all compounds addressing at least 4 ABC transporters (11 in total); and (iii) selected candidates addressing at least 3 ABC transporters based on chemical diversity and affordability (6 in total). In total, 42 candidates were procured for further biological assessment against a panel of SLCs (Table S1).

### Biological Assessment of Pan-ABC Transporter Modulators against Diverse Panel of SLCs

The 42 pan-ABC transporter modulators (10 μM) were biologically evaluated in our in-house assessment platform comprising the specific MATs NAT, DAT, and SERT, as well as the polyspecific multidrug transporters OCT1–3 and MATE1–2K. All compounds showing at least 20% inhibition (+SEM) in the initial screening were analyzed in-depth at various concentrations. Figure 2 visualizes the screening and in-depth results. The entire collection was active against 75.0% of the assessed transporters, failing only to inhibit the specific MATs NAT and DAT (Figure S1). Four compounds (9.52%) inhibited the specific transporter SERT (Figure 2A). The hit rates were slightly higher against OCT1 and OCT3 (19.0 and 26.2%, respectively; Figure 2B,D) – transporters which generally showed a lower degree of polyspecificity. Against the strongly polyspecific multidrug transporters OCT2 and MATE1–2K, the hit rates were substantially higher (42.9, 45.2, and 52.4%, respectively; Figure 2C,E,F).

**Figure 2:**
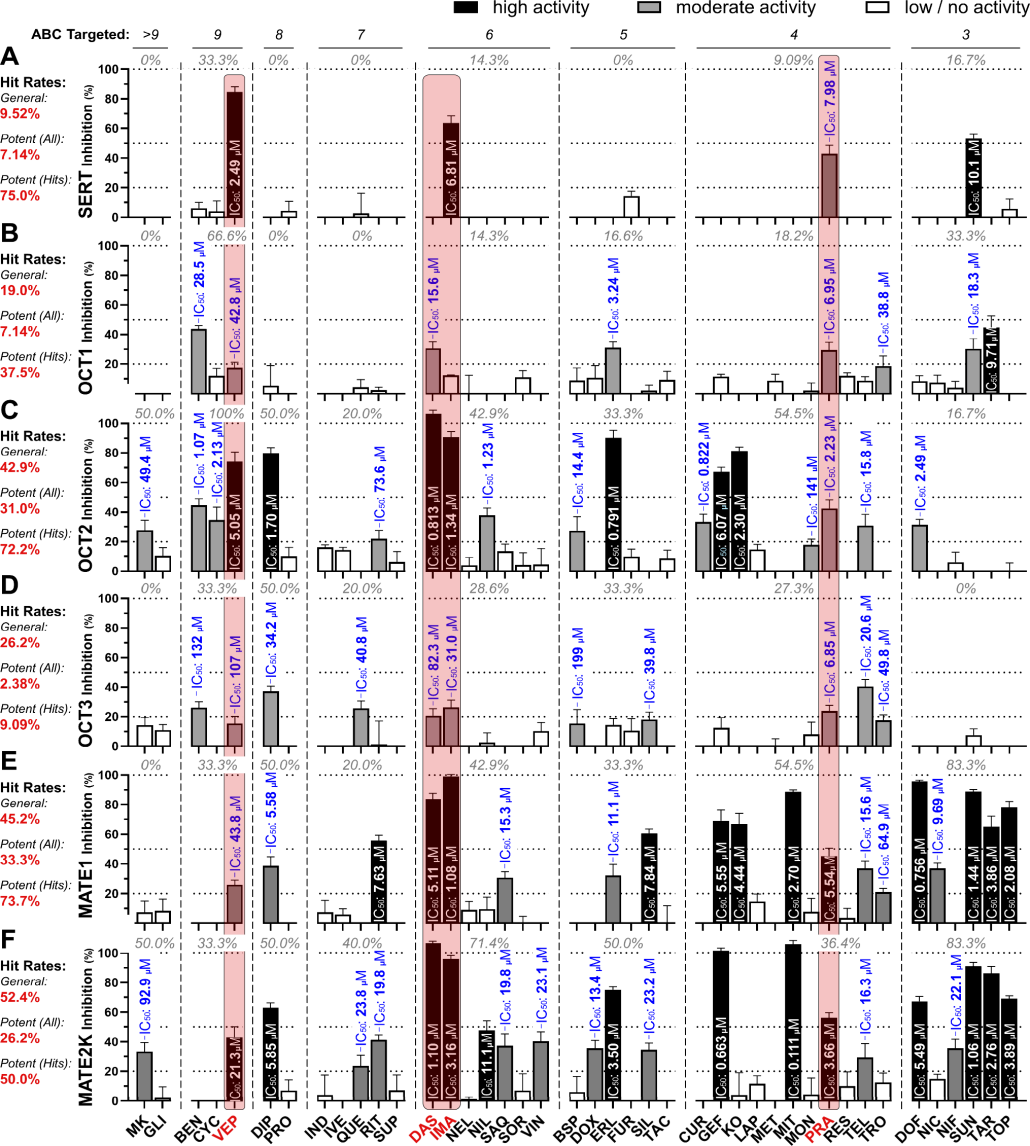
Screening (bar diagrams) and in-depth results (IC_50_ values) of the 42 assessed pan-ABC transporter modulators (screening: 10 μM; in-depth: Various concentrations) against SERT (A), OCT1 (B), OCT2 (C), and OCT3 (D), MATE1 (E), and MATE2K (F). IC_50_ values were determined for all compounds that showed inhibition values of at least 20% (+SEM). Shown are mean ± SEM values of at least three independent experiments.

The average hit rate against these six transporters was 32.5% (range: 9.52–52.4%), while the hit rate of potent (i.e., IC_50_ < 10 μM) compounds was 17.9% (range: 2.38–33.3%) when referred to all 42 test compounds, and 52.9% (range: 9.09–75.0%) when referred to the number of hit molecules (inhibition = 20% + SEM) alone. The latter number indicates that ∼50% of the hit compounds were on average potent inhibitors. Figures S2–S7 show all concentration-effect curves determined, and all relevant numeric values [i.e., IC_50_, maximal inhibition (*I*_max_), Hill slopes, and number of biological replicates] are summarized in Table S2.

From the screening and in-depth analyses, privileged ligands that addressed most of the assessed transporters with comparably high potency could be identified: **VEP** (6 SLCs and 3 SLC families), **DAS** (5 SLCs and 2 SLC families), **IMA** (5 SLCs and 3 SLC families), and **PRA** (6 SLCs and 3 SLC families). Figure 3 provides the respective concentration-effect curves.

**Figure 3:**
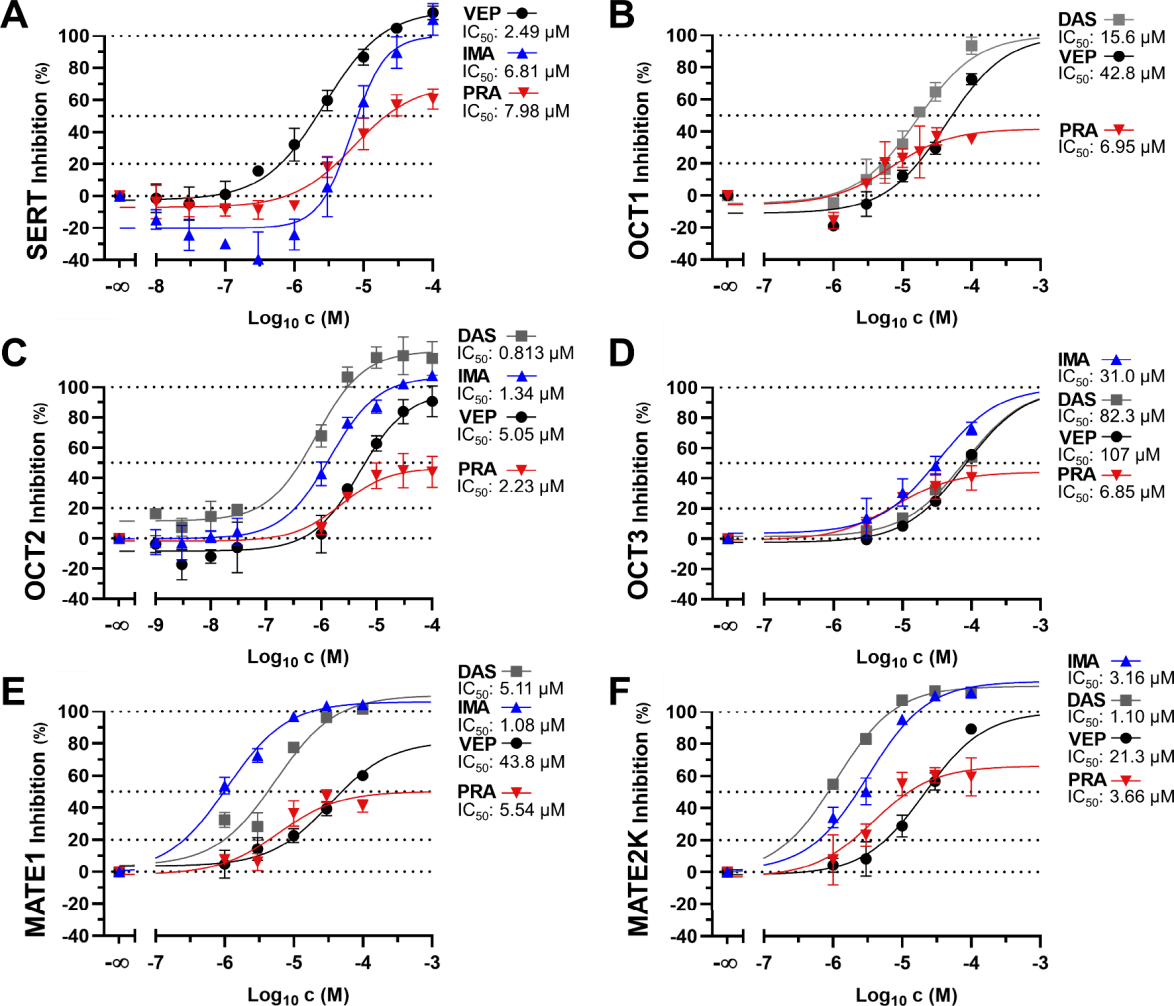
In-depth assessment of identified privileged ligands **VEP** (black closed circles), **DAS** (gray closed squares), **IMA** (blue closed upward triangles), and **PRA** (red closed downward triangles) against SERT (A), OCT1 (B), OCT2 (C), OCT3 (D), MATE1 (E), and MATE2K (F). Shown are mean ± SEM values of at least three independent experiments.

### Correlation Analyses on Polypharmacology and Potency of Privileged Ligands

The results shown in Figure 2 indicate that polypharmacology is a compound property transferable to other protein superfamilies. We performed statistical analyses with the acquired biological data to substantiate this hypothesis. In a first step, the numbers of individually targeted SLCs (0, 1, ···, 6; Figure 4A) and the numbers of targeted SLC families (0, 1, 2, 3; Figure 4B) were plotted against either the numbers of individual ABCs or ABC families targeted according to Table S1. While no correlation could be observed for the plots including individual ABC transporters targeted, the regressions of both individual SLCs (*r* ^2^: 0.964; *p*: 0.0184) and SLC families targeted (*r* ^2^: 0.963; *p*: 0.0185) were statistically significant. The slopes of 0.809 and 0.429, respectively, confirm the positive correlations, suggesting that for each targeted ABC family roughly one SLC transporter and half an SLC family are targeted. A ‘leave-one-out’ approach, which determines the contribution of each compound to the resulting data points and slopes (Figure S8), revealed that **PRA** was an outlier. However, a clear positive trend was present even when **PRA** was included.

**Figure 4:**
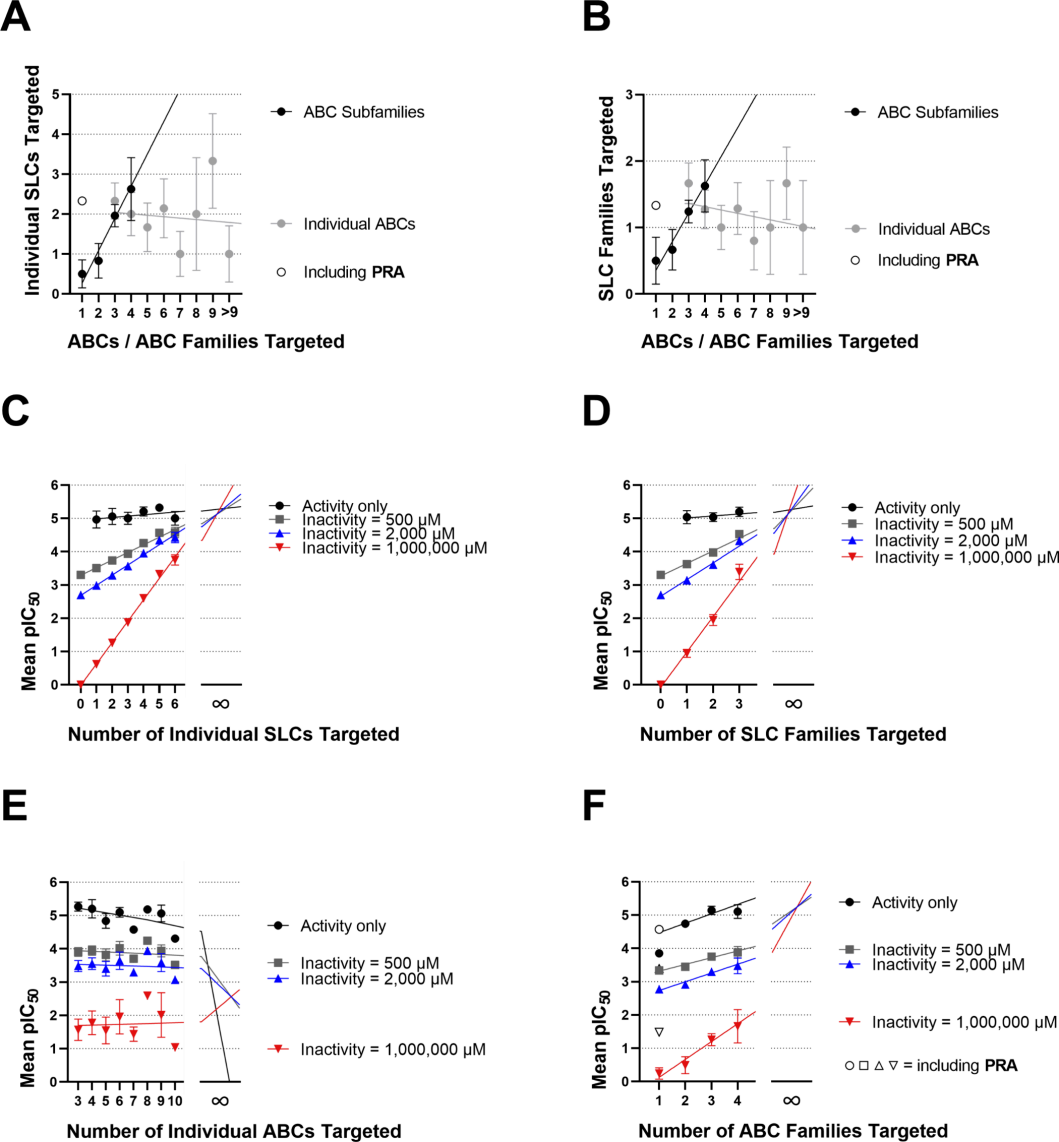
Correlations between herein acquired activity data of the 42 pan-ABC transporter modulators and targeted membrane transporters. (A) Individual SLCs vs individual ABCs (gray closed circles) or ABC families [black closed circles; excluded outlier: **PRA** (*r* ^2^: 0.964; *p*: 0.0184; slope: 0.809); open circle: Data point including **PRA**]. (B) SLC families vs individual ABCs (gray closed circles) or ABC families [black closed circles; excluded outlier: **PRA** (*r* ^2^: 0.963; *p*: 0.0185; slope: 0.429); open circle: Data point including **PRA**]. Furthermore are shown plots with mean pIC_50_ values of compound groups targeting a defined number of individual SLCs (C), SLC families (D), individual ABCs (E), and ABC families (F); black closed circles: Activity values only; gray closed squares: Low activity assumption (inactivity threshold: 500 μM); blue closed upward triangles: Intermediate value assumption (inactivity threshold: 2000 μM); red closed downward triangles: No activity assumption (inactivity threshold: 1,000,000 μM); open symbols (F): Group value including **PRA**. Shown are mean ± SEM values.

Our biological results also indicated that polypharmacology is associated with potency. Grouping the compounds according to the numbers of individual SLCs (Figure 4C), SLC families (Figure 4D), individual ABCs (Figure 4E), and ABC families targeted (Figure 4F) and plotting these numbers against the respective mean pIC_50_ values (i.e., negative decadic logarithm of IC_50_ values as normally distributed surrogate for biological activity/potency) provided positive trends for individual SLCs (Figure 4C), SLC families (Figure 4D), and ABC families targeted (Figure 4F). However, these calculations took only compounds into account which our assessment platform discovered as active. Very weak compounds (i.e., inhibitory effect <20% + SEM, which represents an IC_50_ equivalent of ∼500 μM) are the ‘blind spot’ of our (or any other available) assessment platform. Thus, we performed a robustness analysis to show that the found positive associations are stable even under different assumptions for as inactive identified compounds: (i) Low activity assumption: For ‘inactive’ compounds, an activity value of 500 μM was assumed; (ii) no activity assumption: For ‘inactive’ compounds, an activity value of 1,000,000 μM was assumed; and (iii) intermediate value assumption as used by us before:^36^ For ‘inactive’ compounds, an activity value of 2000 μM was assumed. As demonstrated in Figure 4C,D, the positive slope became more pronounced with increased assumption value. Additionally, an intersection of all lines at higher numbers of individual SLCs and SLC families than those tested was observed. No conclusions could be drawn regarding individual ABCs given the strong deviation (Figure 4E). Considering ABC families (Figure 4F), however, a positive trend toward a common intersection at higher numbers than tested was observed as well.

### Correlation Analyses on Polypharmacology and Privileged Structures

One central question of interest in the field of polypharmacology is whether molecular-structural dependencies exist and whether they may be harnessed in future drug development processes, for example, by either omitting them (i.e., generation of highly selective, specific, and/or pharmacokinetically preferable drugs) or intentionally including them (e.g., generation of multitarget drugs as pharmacological tools or potential therapeutics). In order to address this important question, we performed computer-aided pattern analysis, as described before,^11,36–41^ by searching all 734 available chemical substructures in all 42 tested compounds visualized as a binary code (i.e., ‘1’ = present, ‘0’ = not present; Table S3).

Among the 42 compounds, 350 substructures were absent (Table S4); including these substructures in future drug design would increase the chance to obtain compounds with minor ABC- and SLC-targeting properties. However, it should be taken note that the original data set included ABC transporter modulators that targeted at least 3 ABCs,^35^ and most of the procured compounds targeted 4 ABCs or more (Table S1). Consequently, the distribution of these 350 substructures is unknown for dual-targeting, selective, or inactive compounds. Furthermore, many of these 350 substructures (e.g., 9-deazapurine^42^ and thienopyridine^43^) have been found in compounds with pronounced multitarget activity against ABC transporters before; thus, the number of 42 compounds is a limitation of this analysis. We cross-checked the 350 substructures and searched them in the entire data set of 280 multitarget ABC transporter modulators,^35^ and identified 169 substructures that ultimately occurred in at least one compound. Figure 5A shows a selection of substituents, linkers, and basic scaffolds with greater relevance to modern drug development that were among the 181 substructures not present in the original data set of 280 pan-ABC transporter modulators.

**Figure 5:**
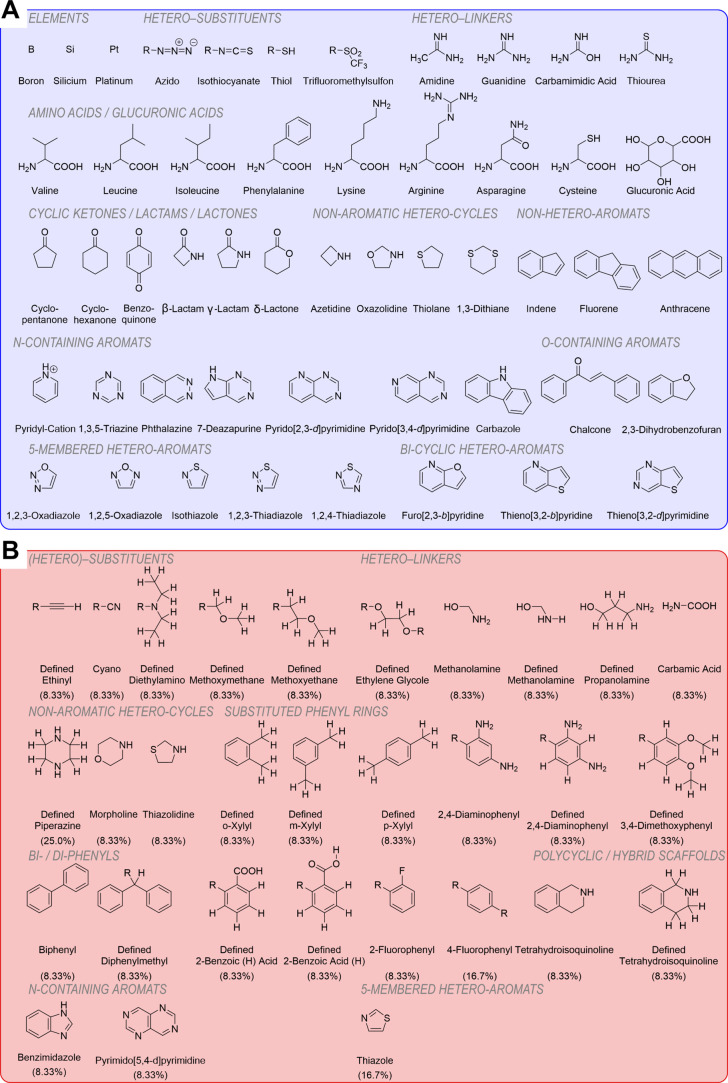
Molecular substructures associated with multitarget inhibition of ABCs and SLCs. (A) Selected substructures from 181 and 350 substructures that did not occur in the populations of 280 pan-ABC transporter modulators and 42 test compounds presented in this study, respectively. (B) The 30 most pronounced substructures among the 384 substructures that occurred at least once within the set of 42 pan-ABC transporter modulators.

On the other hand, we identified 384 substructures that occurred at least once within the 42 test compounds (Table S5). To identify the most relevant ones, we split the entire compound population of 42 pan-ABC transporter modulators into two subpopulations: (i) Compounds that inhibited ≥3 ABC families, ≥3 individual SLCs, and ≥2 SLC families (i.e., **VEP**, **DIP**, **RIT**, **IMA**, **DAS**, **ERL**, **TEL**, **GEF**, **TRO**, **SUN**, **DOF**, and **TAR**); and (ii) all other pan-ABC transporter inhibitors of Table S2. We sorted the substructures for each group according to occurrence [i.e., how many times the respective substructure occurred in the (i) 12 and (ii) 30 compounds of each group] and calculated the percentage (100–0%). In total, we identified 186 and 354 substructures, respectively, present in both populations. The difference in these numbers can be explained by the different population size and, thus, the difference in chemical diversity. We were mostly interested in those substructures that occurred most frequently within the 12 privileged ligands that did not occur in the other 30 test compounds. We compared the two subpopulations and identified 30 substructures of the 12 molecules that occurred in at least 1 molecule (Figure 5B). Of particular interest are those occurring in several molecules, i.e., *H*-defined piperazine (3), 4-fluorophenyl (2), and thiazole (2). These privileged substructures found the molecular basis of the collective polypharmacology against ABCs and SLCs particularly in light of the biological evaluations presented.

### Uptake Experiments Reveal Some Privileged Ligands to be Polysubstrates

Inhibition of transporters inevitably raises the question whether the observed effects are based on competitive inhibition of the model substrates (e.g., MPP^+^ and ASP^+^), and whether the test compounds themselves are substrates. Polypharmacological drugs could in this context be referred to as ‘polysubstrates’, i.e., substrates of several transporters. Particularly the question whether a polysubstrate of different SLC families could be discovered, potentially mediating between both specific (e.g., MATs) and polyspecific (e.g., OCTs) transporters, is of importance in this field. In order to investigate these questions, we performed uptake experiments with selected privileged ligands using LC-MS/MS, and the results are shown in Figure 6.

**Figure 6:**
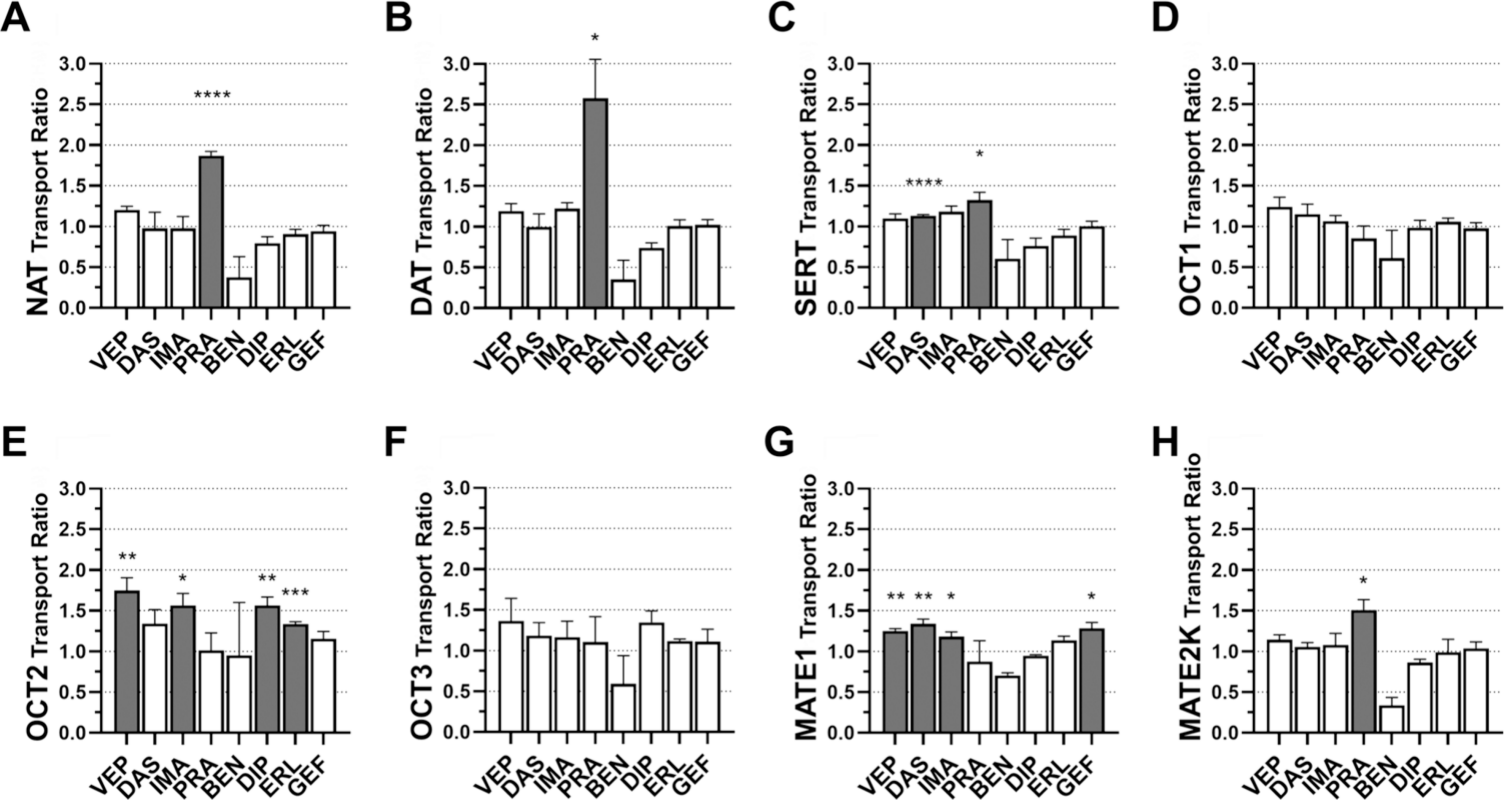
Cellular uptake experiments with selected privileged ligands using LC-MS/MS and HEK293 cells expressing NAT (A), DAT (B), SERT (C), OCT1 (D), OCT2 (E), OCT3 (F), MATE1 (G), or MATE2K (H). Significance was calculated using a multiple *t* test and is given as *: *p* ≤ 0.05, **: *p* ≤ 0.01, ***: *p* ≤ 0.001, and ****: *p* ≤ 0.0001. Shown are mean ± SEM values of at least three independent experiments.

**PRA** exhibited transport ratios of 1.87 and 2.58 for NAT and DAT, respectively. Given the lack of response in the inhibition assays (Figure S1), it is possible that **PRA** is either cotransported together with the NAT and DAT substrate MPP^+^ or channels a completely independent path through these transporters. **PRA** was also transported by SERT (ratio: 1.32), and its inhibition of SERT-mediated MPP^+^ transport suggests competitive inhibition. Despite its consistent inhibitory activity against the multidrug transporters of our assessment platform, only MATE2K showed moderate **PRA** transport (ratio: 1.51) – outlining **PRA** as a rare and new example of a polysubstrate spanning between specific and polyspecific transporters. These results could also explain why **PRA** showed partial inhibition only (*I*_max_ span: 69.9–41.6%; Figure 3): Apart from its discovered role as a substrate for SERT and MATE2K, **PRA** could in parallel to its inhibition have a stimulatory effect on OCT1–3 and MATE1 at particularly higher concentrations, disabling maximal inhibition – which could also be true for **ERL** (OCT1), **BEN** (OCT2), **CUR** (OCT2), **CYC** (OCT2), **DOF** (OCT2), **NIL** (OCT2), **DIP** (MATE1), and **ERL** (MATE1). An apparent activation (lower ‘inhibition effect values’ than 0% as defined by nontreated, transfected HEK cells) by **IMA** (SERT), **ERL** (OCT2), and **KO** (OCT2) support this hypothesis. Another explanation could be a limited solubility of the compounds as this was hypothesized to at least contribute to this effect of ‘partial inhibition’.^44^ However, this is unlikely given the inconsistent effects throughout the panel of SLCs assessed. It should be considered that ‘partial inhibition’ and ‘activation’ are controversially discussed in the transporter communities, and no satisfactory explanation has been found for these effects until today.

As for the other privileged ligands, OCT2 showed transport of **VEP** (ratio: 1.77), **IMA** (ratio: 1.57), **DIP** (ratio: 1.56), and **ERL** (ratio: 1.35), while MATE1 demonstrated transport of **VEP** (ratio: 1.25), **DAS** (ratio: 1.34), **IMA** (ratio: 1.18), and **GEF** (ratio: 1.28).

### Using Privileged Ligands to Target Barely Druggable Transporters

Privileged ligands may not only address several pharmacological targets of structural, functional, and/or phylogenetic distance simultaneously, they may also project this ability to yet undruggable (i.e., as yet no modulators available, and thus currently not chemically/pharmacologically tractable^9^) or barely druggable (i.e., only very few modulators available) pharmacological targets.^19,26^ We screened our in-house biological libraries and identified two membrane transporters that qualified for further assessment:

(i) ABCA1, an important ABC transporter in various diseases, for which, before our recent reports,^25,26,38^ only 14 inhibitors with mostly very poor potencies (i.e., triple-digit micromolar concentrations) were known;^5^ and
(ii) Oatp1d1, an important multidrug transporter influencing pharmacokinetics and detoxification in zebrafish regularly used in drug development as in vivo assessment platform.^25,45^ Only 78 mostly very weak (i.e., double- to quadruple-digit micromolar concentrations) Oatp1d1 modulators have been identified until today.^25^

**VEP**, **DAS**, **IMA**, and **PRA** were used as model privileged ligands. The protein kinase inhibitors (PKIs) **DAS** and **IMA** showed very strong inhibitory activities against ABCA1 (Figure 7A; IC_50_: 4.56 and 1.95 μM, respectively), rendering these compounds as two of the most potent ABCA1 inhibitors reported so far.^5,25,26,38^ Similarly, **PRA** revealed high inhibitory activity against Oatp1d1 (IC_50_: 4.29 μM) making it one of the most potent Oatp1d1 inhibitors in the literature.^25^

**Figure 7:**
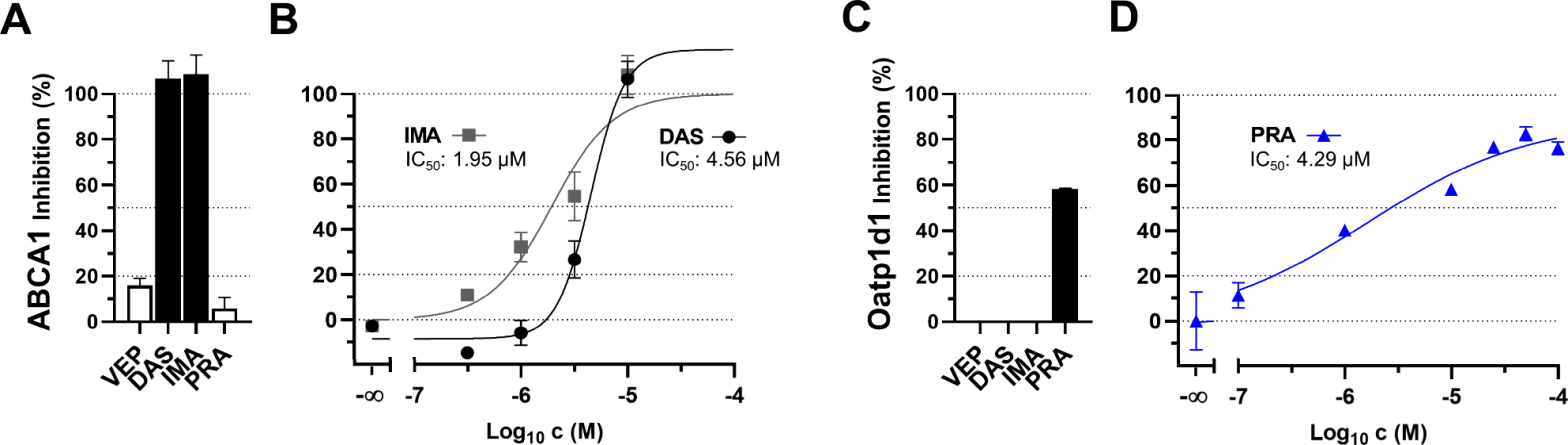
Assessment of the privileged ligands **VEP**, **DAS**, **IMA**, and **PRA** against the barely druggable transporters ABCA1 (A, B) and zebrafish Oatp1d1 (C, D).^25^ Shown are the initial screenings (A, C) and in-depth analyses (B, D) applying cell-based functional assays using fluorescent 25-NBD-cholesterol (A, B) or radiolabeled [^3^H]­BSP (C, D) and J774A.1 (A, B) or HEK293-Oatp1d1 (C, D) cells. Shown are mean ± SEM values of at least four independent experiments.

### Bioinformatic, Spectroscopic, and Toxicological Analyses of Test Compounds

In order to assess the specificity of the observed effects by the 42 tested drugs and drug-like compounds, we conducted comprehensive complementary assessments. First, we analyzed the molecular structures of the compounds for pan-assay interference compound (PAINS) substructures, which may indicate increased chemical reactivity and/or instability. From the 42 tested compounds, only a minor fraction (16.7%, seven compounds) had such substructures. Five of these are approved drugs, rendering them ultimately suitable for therapeutic application despite being flagged as PAINS. Second, only **DIP** (100 μM; weak), **MON** (100 μM; weak), **CUR** (100 μM; weak), **DOX** (10 and 100 μM; strong), **MIT** (100 μM; weak), and **TOP** (100 μM; weak) showed autofluorescence after excitation at 480 nm (±10 nm; Table S7). However, only the effect of **DOX** was significant, and given the fact that LC-MS/MS was applied to assess the 42 pan-ABC transporter modulators for their biological activity against the panel of SLCs, and **DOX** in particular was not tested against ABCA1 in the 25-NBD-cholesterol fluorescence assay, **DOX**’s autofluorescence is not of relevance to the present work. Third, the vast majority of compounds showed no or only minor intrinsic toxicity at 10 and 100 μM after exposure of 2 h (Figure S9A,B), which is much longer than the assay time for all performed SLC experiments (0.5–5 min). In addition, the privileged ligands **VEP**, **DAS**, **IMA**, and **PRA** tested against ABCA1 maintained a cell viability at 10 μM and 72 h of at least ∼50% (Figure S9C).

## Discussion

ABCs and SLCs harbor promising pharmacological targets for which successful medicinal chemistry campaigns^46–53^ and drug development stories are known.^2,54^ However, the vast majorities of ABCs and SLCs (and suggested SLCs^8^) are undruggable (Figure 1); many of which are associated with prevalent and rare diseases.^2,3^ Here, we presented the approach of ‘target class repurposing’ to identify privileged ligands that mediate between ABCs and SLCs to overcome transporter undruggability. We compiled a collection of 280 pan-ABC transporter modulators^35^ and selected 42 chemically and pharmacologically diverse molecules (Table S1). This number seems low at a first glance. However, only 154 of the 280 compounds were commercially available, and our collection represented a significant fraction (27.3%); we procured particularly 76.6% of compounds targeting >3 transporters and 100% of compounds targeting >4 transporters.

In total, 75.0% of investigated SLCs and 100% of investigated SLC families were addressed by these 42 compounds. The average hit rate was 32.5% (range: 9.52–52.4%), and the hit rates increased with enhanced polyspecific character of the transporters (NAT/DAT < SERT < OCT1/3 < OCT2 < MATE1/2K). Although approved drugs bear generally a higher degree of polypharmacology,^27,29,55–57^ and particularly polyspecific transporters (e.g., OCT2, MATE1, and MATE2K) accept a magnitude of ligands, our findings are of significance for six reasons:

(i) Hit rates in double-digit percentages are exceptional even for single-targeted approaches, let alone multitarget approaches^11,40,41^ – particularly considering the lack of structural knowledge or the usually necessary large numbers of compounds to be tested;
(ii) the alignment of different ligand preferences of these transporters is a challenge, particularly taking both specific and polyspecific transporters into account;
(iii) the identification of many potent hits (i.e., IC_50_ ≪ 10 μM; on average 52.9% of hits were potent) came to a surprise, as chemically diverse compound libraries are not expected to provide hit molecules in these activity ranges;
(iv) the literature data associated with the 42 tested compounds was very diverse (e.g., different assessment platforms, broad time span, potentially diverging results, etc.),^35,36,40,41^ and yet, this collective literature data could reliably be used;
(v) apart from rare examples (e.g., **CYC** ^33,34^ ), most test compounds were not described in the context of pan-SLC transporter modulation, ultimately demonstrating translation of polypharmacology between structurally, functionally, and phylogenetically distinct protein families; and
(vi) the collection showed higher activities against polyspecific (19.0–52.4%) than specific (0–9.52%) transporters, which resembled their polypharmacology against ABC transporters (preferably addressing ABCB1, ABCB11, ABCC1, and ABCG2, than, for example, ABCA1, ABCB5, ABCC9, ABCE1, or ABCFs);

Further statistical analysis showed that the polypharmacology of compounds against one protein superfamily (e.g., ABCs) seemed preferably to result in the polypharmacology of these compounds against another protein superfamily (e.g., SLCs), particularly at increased structural, functional, and/or phylogenetic distance (Figure 4A,B). Additionally, polypharmacology went along with potency, as lower IC_50_ values were observed for compounds targeting higher numbers of individual SLCs and SLC families. It was demonstrated earlier that polypharmacological drugs show higher potencies, however, particularly comparably high activities were markedly reduced,^58^ specifically for transporters.^59^

We identified critical chemical patterns that underpin the polypharmacology of pan-ABC transporter inhibitors against various membrane transporters. In total, 30 substructures that promote multitargeting and 350 substructures that impede multitargeting found the molecular basis of polypharmacology against ABCs and SLCs (Figure 5; Supporting Information, Tables S4 and S5). There seemed to be a discrepancy between these numbers. However, it should be taken note that the original C@PA model already highlighted that it is mostly the ‘negative multitarget fingerprint’ (i.e., the collective substructures identified to impede multitargeting) that discriminated between activity and inactivity against multiple targets, while the ‘positive multitarget fingerprint’ (i.e., collective substructures identified to promote multigargeting) rather shaped the chemical composition of the output molecules.^41^ It is noteworthy that we identified fluorine-substituted phenyl rings as strongly polypharmacology promoting. This may have strong implications in modern drug design since the ‘*F*-walk’ is one major branch of organic synthesis strategies to elucidate structure–activity relationships and to optimize target engagement. Similarly, morpholine – regularly used to increase drug solubility – is also associated with multitarget membrane transporter inhibition. One striking finding is the identification of *H*-defined piperazine (present in **DAS**, **IMA**, and **DOF**) as polypharmacology-promoting substructure, while regular (i.e., nondefined) piperazine (present in **DAS**, **IMA**, **KO**, and **DOF**) was present in both groups of the 12 and 30 molecules analyzed. This highlights the importance to consider (position-specific and nonpolar) hydrogens which are usually disregarded in other computational approaches apart from very few exceptions.^60,61^ Thus, C@PA expands the relevant chemical space of substructures, particularly in retrospective SAR analysis (as in the present study) but also in prospective computational prediction models and modern drug development.

**PRA** was identified as a polysubstrate that bridged between specific (i.e., MATs) and polyspecific (i.e., MATE2K) transporters (Figure 6), supporting the hypothesis of common structural motifs among otherwise structurally, functionally, and/or phylogenetically distant transporters addressable by privileged ligands. As proof-of-concept, we screened our in-house cell biology and identified two transporters that fulfilled the criteria for being almost undruggable, i.e., ABCA1 ^5,25,26,38^ and Oatp1d1.^25,45^ Notably, **DAS** and **IMA** as well as **PRA** could be identified as three of the most potent inhibitors of ABCA1 as well as Oatp1d1, respectively.^5,25,26,38,45,62–67^

The use of polypharmacological drugs that unselectively interact with a broad array of targets is often perceived as not favorable and as not having practical utility. However, in many cases of unavailability of hit or lead structures for undruggable or barely druggable pharmacological targets, e.g., SLCs, polypharmacology represents a valuable source of privileged ligands. Since many drugs are easily commercially available, they represent an affordable and sustainable solution for drug development. Many herein presented privileged ligands are approved drugs, and thus, these may be used off-label or repurposed for certain diseases associated with undruggable or barely druggable SLCs–providing them with immediate clinical implications. Since approved drugs harbor anyway a high degree of polypharmacology,^27,29,55–57^ the clinical efficacy of drugs may even be supported by polypharmacology as such.^27^ The well-described organic synthesis of most drugs enables hit-to-lead optimization to design-out their polypharmacology^17^ – which makes this approach also relevant for medicinal chemistry beyond state-of-the-art drug repurposing which has already been demonstrated in the past.^68,69^ On the other hand, optimization and ″design-out” are not necessarily required for target validation if proper cellular models, e.g., HEK293 cells (over)­expressing the target-of-interest, are established.

## Conclusions

Our studies demonstrated that target class repurposing is a valid strategy to gain chemically novel and active compounds with high originality and high potency for drug targets of structural, functional, and/or phylogenetic distance. Specifically the translation of their polypharmacology toward an unknown and new pharmacological target (human or non-human) landscape represents an added value to the current experimental pharmacology and medicinal chemistry repertoire of methodologies, holding the key to unlock undruggable pharmacological targets by addressing common structural motifs for future therapeutic and diagnostic development.

## Materials and Methods

### Bioinformatic Compound Selection

The National Center for Biotechnology Information (NCBI; https://www.ncbi.nlm.nih.gov) database was manually searched for every single ABC transporter (ABCB1, ABCC1, ···, ABCG5/8) in combination with key terms (e.g., ‘inhibitor’, ‘activator’, or ‘modulator’), and particularly (almost) undruggable ABC transporters for which scarce information (including a few modulators only) is available (e.g., the A family) were searched for in various other databases, including PubChem (https://pubchem.ncbi.nlm.nih.gov), ChEMBL (https://www.ebi.ac.uk/chembl), UniProt (https://www.uniprot.org), and DrugBank (https://go.drugbank.com). Summarizing literature of ABC transporter families (i.e., ABCA,^5^ ABCB,^70^ ABCC,^33,71,72^ and ABCG^73^) as well as recent multitarget data sets^36–39^ were also taken into account. Compounds were selected that showed an inhibitory, activatory, or other modulatory interaction with the respective transporters or their energy-supplying unit, the ABC transporter ATPase. In addition, substrates and compounds that increased the susceptibility of cell lines expressing the respective transporter against antineoplastic agents were also considered. From the 280 identified small-molecules that targeted at least three different ABCs,^35^ half was commercially available. For the present study, 42 drugs and drug-like compounds have been selected [Figure 8 (graphic generated using iGraph^74^) and Table S1] according to defined criteria: (i) targeting at least three different ABCs (preferably different families); (ii) the entire collection must target the currently druggable ABC transporter proteome (‘ABC-ome’); (iii) the entire collection must be chemically diverse (e.g., naturally derived compounds and synthetic drugs); (iv) the entire collection must harbor diverse drug development stages (e.g., approved drugs and experimental compounds); and (v) commercial availability and affordability.

**Figure 8:**
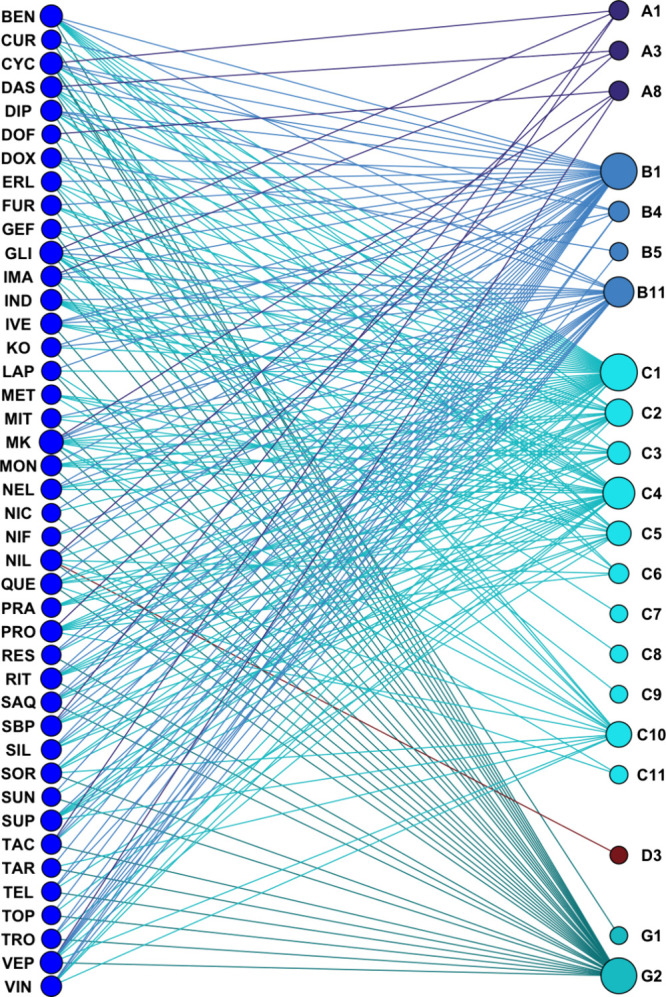
Bioactivity network of the 42 pan-ABC transporter modulators as listed in Table S1 (blue circles; left) and the entire druggable ABC transporter proteome (‘ABC-ome’; blueish/greenish circles, right). Red: Rare case of regulation of an ABCD transporter, ABCD3, by **NIL**. The circle size represents the polypharmacology of the compounds (blue, left) and the polyspecificity of ABCs (blueish/greenish, right).

### Chemicals

The SLC probe substrates 1-methyl-4-phenylpyridinium (MPP^+^) for NAT, DAT, SERT, and MATE1–2K, as well as 4-(4-(dimethylamino)­styryl)-*N*-methylpyridinium (ASP^+^) for OCT1–3 were purchased from Sigma-Aldrich (St. Louis, MO, USA). The 42 procured and studied pan-ABC transporter modulators benzbromarone (**BEN**; PubChem CID 2333), curcumin (**CUR**; PubChem CID 969516), cyclosporine A (**CYC**; PubChem CID 5284373), dasatinib (**DAS**; PubChem CID 3062316), dipyridamole (**DIP**; PubChem CID 3108), dofequidar (**DOF**; PubChem CID 213040), doxorubicin (**DOX**; PubChem CID 31703), erlotinib (**ERL**; PubChem CID 176870), furosemide (**FUR**; PubChem CID 3440), gefitinib (**GEF**; PubChem CID 123631), glibenclamide (**GLI**; PubChem CID 3488), imatinib (**IMA**; PubChem CID 5291), indomethacin (**IND**; PubChem CID 3715), ivermectin (**IVE**; PubChem CIDs 6321424 and 6321425), Ko143 (**KO**; PubChem CID 10322450), lapatinib (**LAP**; PubChem ID 208908), methotrexate (**MET**; PubChem CID 126941), mitoxantrone (**MIT**; Pubchem CID 4212), MK-571 (**MK**; PubChem CID 5281888), montelukast (**MON**; PubChem CID 5281040), nelfinavir (**NEL**; PubChem CID 64143), nicardipine (**NIC**; PubChem CID 4474), nifedipine (**NIF**; PubChem CID 4485), nilotinib (**NIL**; PubChem CID 644241), pranlukast (**PRA**; PubChem CID 4887), probenecid (**PRO**; PubChem CID 4911), quercetin (**QUE**; PubChem CID 5280343), reserpine (**RES**; PubChem CID 5770), ritonavir (**RIT**; PubChem CID 392622), saquinavir (**SAQ**; PubChem CID 441243), silymarin (**SIL**; PubChem CID 5213), sorafenib (**SOR**; PubChem CID 216239), sulfinpyrazone (**SUP**; PubChem CID 5342), sulfobromophthalein (**SBP**; PubChem CID 5345), sunitinib (**SUN**; PubChem CID 5329102), tacrolimus (**TAC**; PubChem CID 445643), tariquidar (**TAR**; PubChem CID 148201), telmisartan (**TEL**; PubChem CID 65999), topotecan (**TOP**; PubChem CID 60700), troglitazone (**TRO**; PubChem CID 5591), verapamil (**VEP**; PubChem CID 2520), and vinblastine (**VIN**; PubChem CID 13342) as well as all other chemicals for cell culture were obtained from Sigma-Aldrich (St. Louis, MO, USA). The test compounds were stored at −20 °C (10 mM stocks) in dimethyl sulfoxide (DMSO).

### Cell Culture

Human embryonic kidney (HEK) 293 cells expressing NAT, DAT, SERT, OCT1, OCT2, OCT3, MATE1, or MATE2K were generated by stable transfection using the Flp-In system (ThermoFisher Scientific, Waltham, MA, US). The generation of HEK293-NAT, HEK293-DAT, and HEK293-SERT cells, their characterization, and their initial application has been described in detail previously.^75^ Transfected and nontransfected HEK293 cells were cultured in Dulbecco’s Modified Eagle’s Medium (DMEM) supplemented with 10% fetal bovine serum (FBS), streptomycin (100 μg/mL), penicillin G (100 units/mL), and _L_-glutamine (4.5 mM; all ThermoFisher Scientific, Waltham, MA, US). The cells were stored in liquid nitrogen (FBS: 90%; DMSO: 10%) and cultivated at 37 °C under a 5% CO_2_-humidified atmosphere. A trypsin-EDTA solution (0.05%/0.02%; ThermoFisher Scientific, Waltham, MA, US) was used to detach the cells for either subculturing or biological investigation at a confluence of ∼90%. Cell counting was performed with a Neubauer cell counting chamber (ThermoFisher Scientific, Waltham, MA, US).

J774A.1 macrophages expressing the murine ABCA1 ortholog were obtained from American Type Culture Collection (ATCC; TB-67). Cultivation was performed using DMEM (Biowest, Nuaillé, France) supplemented with 10% (v/v) FBS.^26,38^

The stably transfected HEK293 cells recombinantly overexpressing the zebrafish uptake transporter drOatp1d1 were established as described previously.^45^

### Transport Inhibition Assays

#### MATs, OCTs, and MATEs

The 42 procured pan-ABC transporter modulators were studied using state-of-the-art in vitro assays assessing NAT, DAT, SERT, OCT1–3, and MATE1–2K function as described earlier:^26^ 48 h prior to the experiment, 300,000 transfected HEK293 cells per well were plated into poly-_D_-lysine precoated 24-well plates (Greiner Bio-One, Kremsmünster, Austria). Every plate contained two wells of empty vector-transfected cells as a control to account for transporter-independent uptake of the probe substrates (either MPP^+^ or ASP^+^). On the day of the experiment, the cells were washed once with prewarmed (37 °C) Hanks’ Balanced Salt solution (HBSS; ThermoFisher Scientific, Waltham, MA, US) supplemented with 10 mM HEPES at pH 7.4 (Sigma-Aldrich, Taufkirchen, Germany) adjusted to pH 7.4 (Sigma-Aldrich, Taufkirchen, Germany), hereafter referred to as HBSS^+^. Cells expressing MATE1–2K were additionally incubated with 30 mM ammonium chloride (NH_4_Cl) in HBSS^+^ for 30 min to change the direction of transport. Dilution series of the 42 procured pan-ABC transporter modulators were performed in DMSO and subsequently diluted in HBSS^+^ (final DMSO concentration: 1%). The cells were eventually exposed to 250 μL of a solution containing on the one hand the SLC probe substrates MPP^+^ (NAT, DAT, and SERT: 0.2 μM; MATE1–2K: 2.0 μM; Sigma-Aldrich, Taufkirchen, Germany) or ASP^+^ (OCT1–3: 2.0 μM; Sigma-Aldrich, Taufkirchen, Germany) and on the other hand the respective test compound at concentrations between 0.1–100 μM in HBSS^+^ for 5 min (NAT, DAT, SERT, OCT1–3) or 0.5 min (MATE1–2K). By the addition of ice-cold HBSS^+^, the incubation was stopped. After the cells were washed twice with ice-cold HBSS^+^, they were lysed using 80% acetonitrile (MeCN; 10 min; LGC Standards, Wesel, Germany) using a 3D platform shaker (Heidolph, Schwabach, Germany). For MPP^+^, the lysate was centrifuged for 15 min and 50 μL of the supernatant were transferred into a 96-well plate, evaporating MeCN at 40 °C under nitrogen flow, and redissolving the samples in 200 μL formic acid [HCOOH; 0.1% (v/v)] followed by liquid chromatography coupled to tandem mass spectrometry (LC-MS/MS) measurement. MPP^+^ isolation was achieved using a Brownlee SPP RP-amide column (4.6 mm × 100 mm inner dimension; particle size: 2.7 μm) with a C18 precolumn in a Shimadzu Nexera HPLC system with autosampler (SIL-30AC), column oven (CTO-20AC), pump (LC-30AD), and controller (CBM-20A; all Shimadzu, Kyoto, Japan). Mobile phase: 80.0% HCOOH (0.1%), 17.2% MeCN, 2.8% methanol (MeOH); flow rate: 0.3 mL/min; oven temperature: 40 °C; detection: API 4000 tandem mass spectrometer [MRM mode; AB SCIEX, Darmstadt, Germany; retention time: 3.5 min; first quadrupole mass: 170.016 Da; third quadrupole mass: 128.1 (102.2) Da; declustering potential: 10 V; collision energy: 42 (63) V; collision cell exit potential: 8 (6) V; internal standard: Fenoterol; integration and quantification software: Analyst (version 1.6.2; AB SCIEX, Darmstadt, Germany)]. For ASP^+^, the cell lysate was transferred into a black 96-well plate, and fluorescence intensity was measured using a Tecan Ultra fluorescence microplate reader (excitation: 482 nm; emission: 612 nm; Tecan, Crailsheim, Germany).

#### ABCA1

ABCA1 transport activity was determined as described previously.^25,26,38^ In short, 160 μL of a J774A.1 cell suspension (45,000 cells per well) were incubated with test compound (i.e., **VEP**, **DAS**, **IMA**, or **PRA**; 20 μL) in a clear 96-well plate (Brand, Wertheim, Germany) for 24 h. Subsequently, the fluorescence dye 25-[*N*-[(4-nitro-2,1,3-benzoxadiazol-7-yl) methylamino]-27-norcholesterol (25-NBD-cholesterol; 20 μL; 10 μM, final concentration: 1 μM; Avanti Polar Lipids, Alabaster, AL, USA) was added and incubated for a further 48 h. Inhibition of ABCA1 reflects an increased fluorescence (i.e., reduced 25-NBD-cholesterol efflux) detected using an Attune NxT flow cytometer (excitation: 488 nm; emission: 530/30 nm; Invitrogen, Waltham, MA, USA).

#### Oatp1d1

Using the prototypic OATP substrate bromosulphophthalein (**BSP**), uptake experiments were performed as described previously.^25,76^ In total, 7 × 10^5^ HEK293-Oatp1d1 as well as HEK293 vehicle control cells were cultivated in poly-_D_-lysine-coated 12-well plates (Sarstedt, Nümbrecht, Germany) for 24 h, followed by exposure to sodium butyrate (10 mM) for another 24 h. Subsequently, the cells were washed with prewarmed (37 °C) uptake buffer (142 mM NaCl, 5 mM KCl, 1 mM K_2_HPO_4_, 1.2 mM MgSO_4_, 1.5 mM CaCl_2_, 5 mM glucose, and 12.5 mM HEPES; pH 7.3). Radiolabeled and unlabeled **BSP** were mixed in uptake buffer to obtain final concentrations of 1 μM and applied without or with added test compounds (i.e., **VEP**, **DAS**, **IMA**, or **PRA**) at various concentrations between 0.1 and 100 μM. After 10 min of incubation, cells were washed with ice-cold uptake buffer (3×) and lysed [0.2% sodium dodecyl sulfate (SDS)] before measuring the intracellular [^3^H]**BSP** concentration by liquid scintillation counting (ThermoFisher Scientific, Waltham, MA, US).

### Computer-aided Pattern Analysis (C@PA)

A substructure catalog of in total 734 substructures was used based on our previous reports.^11,36–41^ These were searched for in all 42 procured and tested compounds using the query search function of InstantJChem version 23.15.3. The results were provided as a binary pattern distribution scheme (i.e., ‘1’ = present, ‘0’ = not present; Table S3).

### Polysubstrate Cell Uptake Experiments

Assay plates were prepared as described for the inhibition experiments. For cell uptake experiments, the cells were washed once with HBSS^+^ (37 °C, ThermoFisher Scientific, Waltham, MA, US). MATE1- and MATE2K-expressing cells were preincubated (30 min) with 30 mM NH_4_Cl to reverse the transport direction. Transporter-transfected and vector control cells were exposed to 2.5 μM of the potential substrate in HBSS^+^ at 37 °C and 5% CO_2_-humidified atmosphere for 2 min (OCTs and MATs) or 1 min (MATE1–2K). Addition of ice-cold HBSS^+^ stopped the transport activity. After two washing steps with HBSS^+^, the cells were lysed with 80% (v/v) MeCN in water (LGC Standards, Wesel, Germany) containing an internal standard for subsequent mass spectrometric analyses. The intracellular accumulation of the potential substrates was measured by LC-MS/MS calculated as the fold change (i.e., transport ratio; dividing uptake into transporter-overexpressing cells by uptake into vector control cells). Total protein quantification per well for normalization purposes was achieved by lysing cells in radioimmunoprecipitation assay buffer (50 mM Tris HCl, 150 mM NaCl, 1.0 mM EDTA, 1% Nonidet P-40, 0.25% sodium deoxycholate, and 0.1% SDS; pH 7.4) and using a BCA assay kit.^77^

### Complementary Compound Assessment

Compound specificity was assessed applying several bioinformatic, spectroscopic, and cell-biological measures: (i) Compound stability and chemical reactivity were assessed with a pan-assay interference compounds (PAINS) substructure search using the SMILES query function of SwissADME (http://www.swissadme.ch),^78^ according to the methodology described by Baell and Holloway;^79^ (ii) potential assay interference in plate reader-based assays [i.e., ASP^+^ (excitation: 482 nm) and 25-NBD-cholesterol (excitation: 488 nm)] was assessed by analysis of emission wavelengths 500–850 nm after excitation at 480 nm (±10 nm);^26^ (iii) intrinsic toxicity of the compounds was determined using a concentration- and time-dependent 3-(4,5-dimethylthiazol-2-yl)-2,5-diphenyltetrazolium bromide-(MTT)-based cell viability assay, as described previously.^80,81^ In short, 20 mL of dilutions of the test compounds (10 and 100 μM) in water were added to 180 μL of a HEK293 (nontransfected) cell suspension into a 96-well flat-bottom plate (Brand, Wertheim, Germany). The suspension was prepared as described above and contained different numbers of cells depending on the respective incubation time (2 h: 50,000 cells per well; 72 h: 1000 cells per well; incubation at 37 °C and 5% CO_2_-humidified atmosphere). DMEM and DMSO defined 100 and 0% cell viability, respectively. After the respective incubation time, 40 μL of an MTT solution (5 mg/mL) were added to each well followed by another incubation of 1 h (37 °C and 5% CO_2_-humidified atmosphere). The supernatant was removed and 100 μL of DMSO were added per well, subsequently measuring absorbance (570 nm; background correction: 690 nm) using a Tecan Ultra microplate reader (Tecan, Crailsheim, Germany).

### Data Analysis and Processing

All experiments were conducted independently at least three times (biological replicates only; no technical replicates) unless stated otherwise due to operational reasons. Compounds that showed at least 20% [+ standard error of the mean (SEM)] inhibition in the initial screenings were assessed again with at least six different concentrations, and their half-maximal inhibition concentration (IC_50_) values were calculated by nonlinear regression using GraphPad Prism (version 8.4.0., San Diego, CA, USA) considering both three- and four-parameter logistic equations, whichever was statistically preferred. Curve fits have been performed as follows: (i) free extrapolation to maximal inhibition given clearly indicated plateau of effect-concentrations; and (ii) if no plateau given by the effect-concentrations, fit to 100% of control mean. The final curves had r^2^ values of at least 0.800. Data was used as normally distributed negative decadic logarithm of the IC_50_ (pIC_50_) values as calculated by GraphPad Prism. After calculation of the mean and SEM, the data was delogarithmized to obtain the final numeric representation as IC_50_ as described earlier.^36^

The physicochemical properties calculated octanol–water partition coefficient (CLogP), molecular weight (MW), molar refractivity (MR), and topological polar surface area (TPSA) were calculated using Molecular Operating Environment (MOE; Chemical Computing Group, Montreal, QC, Canada; version 2024.06).^82^

## Supplementary Material





## Notes

*Literature Data.* Table S1 provides the molecular formulas and pharmacological profiles of the 42 compounds used. *Biological Data.* Table S2 provides the biological data of the 42 compounds as determined within this study. Figures S1–S7 provide screenings and in-depth analyses of the 42 compounds assessed against the selection of SLCs. *Bioinformatic Data.* Tables S3–S5 provide the computer-aided pattern analysis (C@PA) of the 42 compounds. Figure S8 provides all individual plots of the ‘leave-one-out’ method. *Complementary Assessment.* Table S6 provides emission spectra of the 42 compounds assessed for autofluorescence at an excitation wavelength of 480 nm (±10 nm). Figure S9 provides cell viability assay results for the 42 compounds assessed. (XLSX; PDF)

## ABBREVIATIONS

ABC: ATP-binding cassette
ABC-ome: ABC transporter proteome
AI: artificial intelligence
ASP+: 4-(4-(dimethylamino)­styryl)-*N*-methylpyridinium
ATCC: American Type Culture Collection
BCA: bicinchoninic acid assay
CaCl_2_: calcium chloride
C@PA: computer-aided pattern analysis
cDNA: complementary DNA
CLogP: calculated octanol–water partition coefficient
CO_2_: carbon dioxide
DAT: dopamine transporter
DMEM: Dulbecco’s Modified Eagle’s Medium
DMSO: dimethyl sulfoxide
EDTA: ethylenediaminetetraacetic acid
FBS: fetal bovine serum
HBSS: Hanks’ Balanced Salt solution
HCOOH: formic acid
HCl: hydrochloric acid
HEK: human embryonic kidney
HEPES: 4-(2-hydroxyethyl)-1-piperazineethanesulfonic acid
IC_50_: half-maximal inhibition concentration
I_max_: maximal inhibition
K_2_HPO_4_: dipotassium hydrogen orthophosphate
KCl: potassium chloride
LC-MS/MS: liquid chromatography coupled to tandem mass spectrometry
MAT: monoamine transporter
MATE: multidrug and toxin extrusion protein
MeCN: acetonitrile
MeOH: methanol
MgSO_4_: magnesium sulfate
MPP^+^: 1-methyl-4-phenylpyridinium
MOE: Molecular Operating Environment
MR: molar refractivity
mRNA: messenger ribonucleic acid
MTT: 3-(4,5-dimethylthiazol-2-yl)-2,5-diphenyltetrazolium bromide
MW: molecular weight
NaCl: sodium chloride
NAT: noradrenaline transporter
25-NBD-cholesterol: 25-[*N*-[(4-nitro-2,1,3-benzoxadiazol-7-yl) methylamino]-27-norcholesterol
NCBI: National Center for Biotechnology Information
NH_4_Cl: ammonium chloride
Oatp: organic anion transporting polypeptide
OCT: organic cation transporter
PAINS: pan-assay interference compounds
pIC_50_: negative decadic logarithm of the IC_50_
PKI: protein kinase inhibitor
r^2^: correlation coefficient
RT-PCR: real-time polymerase chain reaction
SAR: structure–activity relationship
SDS: sodium dodecyl sulfate
SEM: standard error of the mean
SERT: serotonin transporter
SLC: solute carrier
SMILES: Simplified Molecular Input Line Entry System
TPSA: topological polar surface area.
