# Supplementary material for: Target Class Repurposing Across Membrane Transporter Families Provides Privileged Ligands to Address Specific and Undruggable Pharmacological Targets
Source: ACS Pharmacol Transl Sci. 2026 Jan 27;9(2):332–47. doi: 10.1021/acsptsci.5c00430 (PMC12910493; doi:10.1021/acsptsci.5c00430)
Supplement: Supplementary file 2 [file pt5c00430_si_002.pdf]

SUPPORTING INFORMATION

Target Class Repurposing Across Membrane Transporter Families Provides Privileged Ligands to Address Specific and Undruggable Pharmacological Targets

Muhammad Rafehi [1,2,*], Franziska Tägl [1], Nike Sophia Arlt [1], Maria Neif [1], Katja Stefan [3,4], Wouroud Ismail Al-Khalil [1], Hauke Busch [5], Marius Möller [5], Jörg König [6,7], Vigneshwaran Namasivayam [3,*], Sven Marcel Stefan [3,4,8,*]

[1] University Medical Center Göttingen, Institute of Clinical Pharmacology, Robert-Koch-Str. 40, 37075 Göttingen, Germany

[2] University Hospital of Augsburg and University of Augsburg, Faculty of Medicine, Am Medizincampus 2, 86156 Augsburg, Germany

[3] University of Lübeck and University Medical Center Schleswig-Holstein (UKSH), Lübeck Institute of Experimental Dermatology (LIED), Medicinal Chemistry and Systems Polypharmacology, Ratzeburger Allee 160, 23538 Lübeck, Germany

[4] University of Oslo and Oslo University Hospital, Department of Pathology, Rikshospitalet, Sognsvannsveien 20, 0372 Oslo, Norway

[5] University of Lübeck and University Medical Center Schleswig-Holstein (UKSH), Lübeck Institute of Experimental Dermatology (LIED), Medical Systems Biology, Ratzeburger Allee 160, 23538 Lübeck, Germany

[6] Institute of Experimental and Clinical Pharmacology and Toxicology, Friedrich-Alexander-Universität Erlangen-Nürnberg (FAU), 91054 Erlangen, Germany

[7] FAU NeW - Research Center New Bioactive Compounds, Friedrich-Alexander-Universität Erlangen-Nürnberg (FAU), Nikolaus-Fiebiger-Str. 10, 91058 Erlangen, Germany

[b] Medical University of Lublin, Department of Biopharmacy, Chodzki 4a, 20-093 Lublin, Poland

Corresponding authors:

Muhammad Rafehi	(muhammad.rafehi@med.uni-goettingen.de)
Vigneshwaran Namasivayam	(vigneshwaran.namasivayam@uksh.de)
Sven Marcel Stefan	(sven.stefan@uni-luebeck.de)

Table of Contents

Supplementary Table S1 ……………………………………………………S3–S7
Supplementary Figure S1……………………………………………………S8
Supplementary Figure S2……………………………………………………S9
Supplementary Figure S3……………………………………………………S10–S11
Supplementary Figure S4……………………………………………………S12–S15
Supplementary Figure S5……………………………………………………S16–S18
Supplementary Figure S6……………………………………………………S19–S23
Supplementary Figure S7……………………………………………………S24–S28
Supplementary Figure S8……………………………………………………S29–S31
Supplementary Figure S9……………………………………………………S32
References of Supplementary Table S1..………………………………………S33–S45

Supplementary Table S1.

Compilation of pan-ABC transporter modulators as privileged ligands for target class repurposing toward SLC transporters including their names, abbreviations as used within this report, molecular structures, PubChem CIDs, ChEMBL Compound IDs, DrugBank Accession Numbers, IUPHAR/BPS Guide to Pharmacology Ligand IDs, Chemical Abstract Service (ACS) Numbers, physicochemical properties CLogP, MW, MR, and TPSA as calculated using MOE version 2024.06, the numbers of targeted individual ABC transporters, the numbers of targeted ABC transporter families, as well as example references for each targeted ABC transporter; * = rare case of regulation of an ABCD transporter, ABCD3, by nilotinib (**NIL**).

**Name (Abbreviation) & Molecular Structure**	**PubChem CID**	**ChEMBL Compound ID**	**DrugBank Accession Number**	**IUPHAR / BPS Guide to Pharma-cology Ligand ID**	**Chemical Abstract Service (CAS) Number**	**CLogP**	**MW (g · mol^{-1})**	**MR**	**TPSA (Å)**	**Number of Targeted ABC Transporters**	**Number of Targeted ABC Transporter Families**	**Targeted ABC Transporters (References)**
>9 ABC Targets												
MK-571 (**MK**) 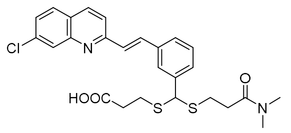	5281888	CHEMBL15177	-	10346	115104-28-4	5.82	515.10	14.42	70.50	12	4	**A8**,[1] **B1**,[2] **B4**,[3] **B11**,[4] **C1**,[5-8] **C2**,[2,6,7] **C3**,[7] **C4**,[6-9] **C5**,[7,10,11] **C10**,[7,12] **C11**,[13] **G2**[2]
Glibenclamide (**GLI**) 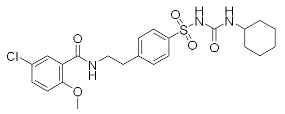	3488	CHEMBL472	DB01016	2414	10238-21-8	4.14	494.01	12.98	113.60	10	4	**A1**,[14] **B1**,[2] **B11**,[4,15] **C1**,[5,7] **C2**,[7] **C5**,[11] **C7**,[16] **C8**,[17] **C9**,[17] **G2**[2]
9 ABC Targets												
Benzbromarone (**BEN**) 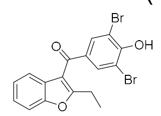	2333	CHEMBL388590	DB12319	-	3562-84-3	5.70	424.09	9.24	50.44	9	3	**B1**,[18] **B11**,[4] **C1**,[5,7] **C2**,[2] **C3**,[19] **C4**,[20,21], **C5**,[7,11] **C6**,[7] **G2**[2,18]
Cyclosporine A (**CYC**) 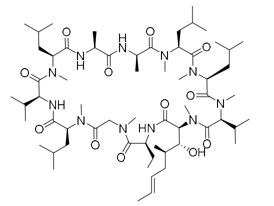	5284373	CHEMBL160	DB00091	1024	79217-60-0	5.29	1202.64	33.08	278.80	9	4	**A1**,[14] **B1**,[22] **B4**,[3] **B11**,[4] **C1**,[22] **C2**,[23] **C10**,[7] **G1**,[24] **G2**[22]
Verapamil (**VEP**) 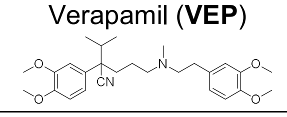	2520	CHEMBL6966	DB00661	2406	52-53-9	5.35	454.61	13.37	63.95	9	4	**A8**,[1] **B1**,[2] **B4**,[3] **B5**,[25] **B11**,[15] **C1**,[5,7,8] **C4**,[21] **C10**,[12] **G2**[2]
8 ABC Targets												
Dipyridamole (**DIP**) 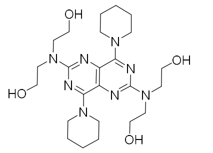	3108	CHEMBL932	DB00975	4807	58-32-2	-0.24	504.64	13.54	145.44	8	3	**B1**,[2] **B4**,[3] **B11**,[4] **C1**,[5] **C2**,[2] **C4**,[8] **C5**,[11] **G2**[2]
Probenecid (**PRO**) 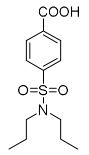	4911	CHEMBL897	DB01032	4357	57-66-9	2.24	285.36	7.54	74.68	8	2	**A8**[1], **C1**,[5,7] **C2**,[7] **C3**,[19] **C4**,[20,26] **C5**,[7,11,26] **C6**,[7,27] **C10**[12]

7 ABC Targets												
Indomethacin (**IND**) 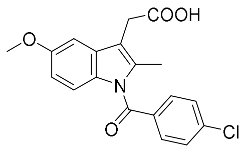	3715	CHEMBL6	DB00328	1909	53-86-1	4.37	357.79	9.64	68.53	7	2	**B11**,[4] **C1**,[5,7] **C2**,[7] **C3**,[19] **C4**,[7,21] **C5**,[10] **C6**[7,27]
Ivermectin (**IVE**) 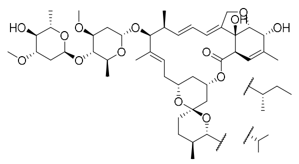	6321424 & 6321425	CHEMBL263291 & CHEMBL552	DB00602	2373	71827-03-7 & 70209-81-3	6.32	875.11	22.90	170.06	7	3	**B1**,[2,28,29] **B4**,[3] **B11**,[28] **C1**,[2,29] **C2**,[2,28] **C3**,[28] **G2**[28,29]
Quercetin (**QUE**) 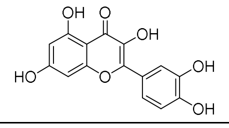	5280343	CHEMBL50	DB04216	5346	117-39-5	2.03	302.24	7.24	127.45	7	3	**B1**,[30] **C1**,[5] **C2**,[31] **C4**,[8] **C5**,[8] **C11**,[32] **G2**[31]
Ritonavir (**RIT**) 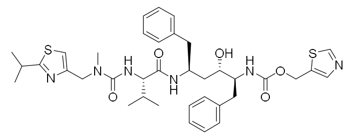	392622	CHEMBL163	DB00503	8804	155213-67-5	5.00	720.96	20.32	145.78	7	3	**B1**,[2,28,29] **B11**,[4,28] **C1**,[5,7,29] **C2**,[28] **C3**,[28] **C4**,[33] **G2**[2,28,29,34]
Sulfinpyrazone (**SUP**) 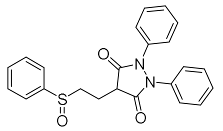	5342	CHEMBL832	DB01138	5826	57-96-5	2.90	404.49	11.56	76.90	7	2	**B11**,[4] **C1**,[5,7] **C2**,[7] **C3**,[19], **C4**,[7,20,26] **C5**,[7,10,11,26] **C10**[35]
6 ABC Targets												
Dasatinib (**DAS**) 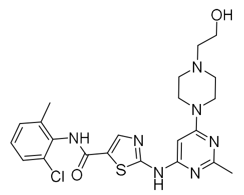	3062316	CHEMBL1421	DB01254	5678	302962-49-8	1.54	488.02	13.06	106.51	6	4	**A3**,[36] **B1**,[37] **C4**,[38] **C6**,[27] **C10**,[39] **G2**[37]
Imatinib (**IMA**) 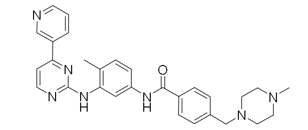	5291	CHEMBL941	DB00619	5687	152459-95-5	2.83	493.61	14.52	86.28	6	4	**A3**,[36] **B1**,[2,37,40] **B11**,[4] **C1**,[5,37,40] **C10**,[37,39] **G2**[2,37,40]
Nelfinavir (**NEL**) 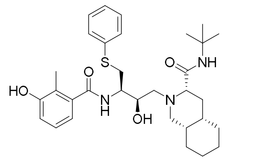	64143	CHEMBL584	DB00220	11090	159989-64-7	5.36	567.79	16.22	101.90	6	3	**B1**,[5] **B11**,[4] **C1**,[5,7] **C2**,[7] **C4**,[33] **G2**[34]
Nilotinib (**NIL**) 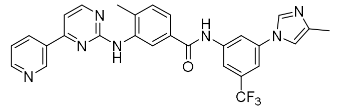	644241	CHEMBL255863	DB04868	5697	641571-10-0	4.34	529.53	14.13	97.62	6 (7)	4 (5)	**A3**,[36] **B1**,[37,40] **C1**,[37,40] **C6**,[27] **C10**,[37,39] **D3**,[41*] **G2**[37,40]
Saquinavir (**SAQ**) 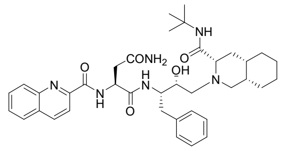	441243	CHEMBL114	DB01232	4813	127779-20-8	3.31	686.90	19.61	166.75	6	3	**B1**,[2] **B11**,[4] **C1**,[5,7] **C2**,[7] **C4**,[33] **G2**[2,34]
Sorafenib (**SOR**) 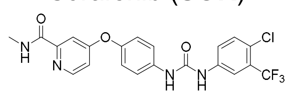	216239	CHEMBL1336	DB00398	5711	284461-73-0	3.73	464.83	11.37	92.35	6	3	**B1**,[37] **C1**,[37] **C2**,[37] **C4**,[37] **C10**,[37] **G2**[37]

Vinblastine (**VIN**) 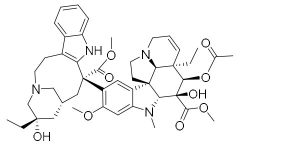	13342	CHEMBL159	DB00570	6851	865-21-4	5.01	810.99	21.97	154.10	6	2	**B1**,[2] **B4**,[3] **B11**,[4] **C1**,[5,7] **C2**,[7] **C10**,[7]
5 ABC Targets												
Bromosulfophthaleine (**BSP**) 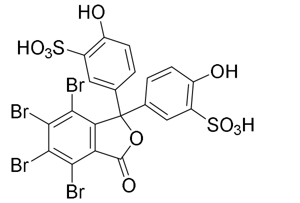	5345	CHEMBL574431	DB13215	4506	297-83-6	5.86	794.04	13.88	175.50	5	3	**A1**,[14] **B11**,[4] **C2**,[2,7] **C4**,[26] **C5**[26]
Doxorubicin (**DOX**) 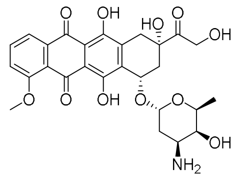	31703	CHEMBL53463	DB00997	7069	23214-92-8	0.51	543.52	13.28	206.07	5	2	**B1**,[42] **B5**,[25] **C1**,[5] **C4**,[43] **C10**[44]
Erlotinib (**ERL**) 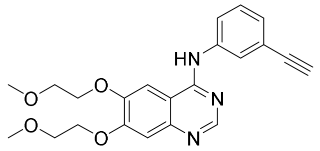	176870	CHEMBL553	DB00530	4920	183321-74-6	2.34	393.44	11.19	74.73	5	3	**B1**,[37,40] **C1**,[40] **C4**,[9] **C10**,[37,39] **G2**[37,40]
Furosemide (**FUR**) 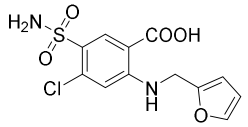	3440	CHEMBL35	DB00695	4839	54-31-9	0.82	330.75	7.74	122.63	5	3	**B1**,[45] **C1**,[5] **C2**,[7] **C4**,[7,20,46] **G2**[46]
Silymarin (**SIL**) 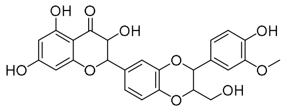	5213	CHEMBL1401508	DB09298	12449	65666-07-1	2.64	482.44	11.96	155.14	5	2	**B1**,[47] **B11**,[4] **C1**,[5] **C4**,[8] **C5**[8]
Tacrolimus (**TAC**) 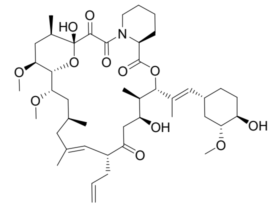	445643	CHEMBL269732	DB00864	6784	104987-11-3	4.57	804.03	21.58	178.36	5	4	**A1**,[14] **B1**,[48] **B11**,[49] **C1**,[22] **G2**[22]
4 ABC Targets												
Curcumin (**CUR**) 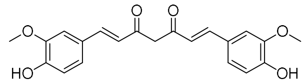	969516	CHEMBL140	DB11672	7000	458-37-7	3.72	368.38	10.13	93.06	4	3	**B1**,[50] **C1**,[22] **C5**,[51] **G2**[22]
Gefitinib (**GEF**) 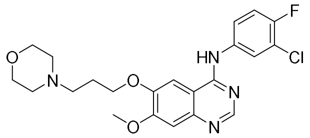	123631	CHEMBL939	DB00317	4941	184475-35-2	3.15	446.91	11.85	68.74	4	3	**B1**,[2,37,40] **C1**,[37,40] **C4**,[9] **G2**[2,37,40]
Ko143 (**KO**) 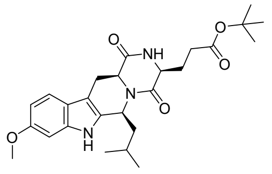	10322450	CHEMBL488910	-	10007	461054-93-3	3.87	469.58	12.92	100.73	4	3	**B1**,[2] **B11**,[4] **C1**,[5] **G2**[2]

Lapatinib (**LAP**) 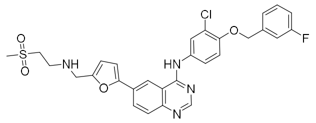	208908	CHEMBL554	DB01259	5692	231277-92-2	4.84	581.07	15.52	106.35	4	3	**B1**,[37] **C1**,[5] **C10**,[37] **G2**[37]
Methotrexate (**MET**) 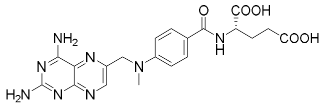	126941	CHEMBL34259	DB00563	4815	59-05-2	-0.44	454.45	11.60	210.54	4	1	**C3**,[19] **C4**,[21] **C5**,[52] **C10**[44]
Mitoxantrone (**MIT**) 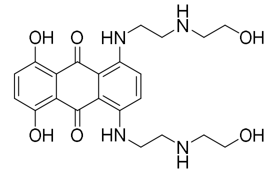	4212	CHEMBL58	DB00563	7242	65271-80-9	-0.54	444.49	11.75	163.18	4	3	**B11**,[49] **C1**,[5] **C4**,[43] **G2**[53]
Montelukast (**MON**) 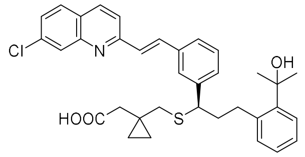	5281040	CHEMBL787	DB00471	3340	158966-92-8	9.15	586.20	17.03	70.42	4	1	**C1**,[5,6] **C2**,[6] **C3**,[6] **C4**[6]
Pranlukast (**PRA**) 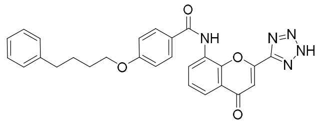	4887	CHEMBL21333	DB01411	3634	103177-37-3	4.44	481.51	13.52	119.09	4	1	**C1**,[5-7] **C2**,[6] **C3**,[6] **C4**[6]
Reserpine (**RES**) 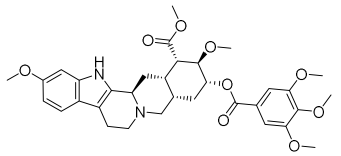	5770	CHEMBL772	DB00206	4823	50-55-5	3.52	608.69	16.23	117.78	4	3	**B1**,[22] **B11**,[4] **C1**,[54] **G2**[55]
Telmisartan (**TEL**) 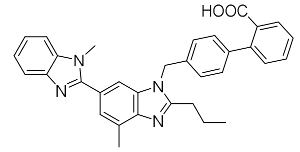	65999	CHEMBL1017	DB00966	592	144701-48-4	8.49	514.63	15.48	72.94	4	3	**B1**,[56] **B11**,[4] **C5**,[57] **G2**,[56]
Troglitazone (**TRO**) 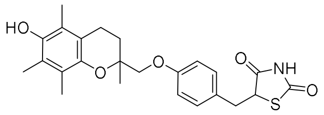	5591	CHEMBL408	DB00197	2693	97322-87-7	4.94	441.55	12.08	84.86	4	3	**B1**,[58] **B11**,[59] **C4**,[60] **G2**[58]
3 ABC Targets												
Dofequidar (**DOF**) 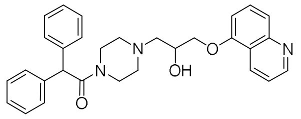	213040	CHEMBL65067	DB14067	-	129716-58-1	3.86	481.60	14.26	65.90	3	3	**A8**,[1] **C1**,[5] **G2**,[61]
Nicardipine (**NIC**) 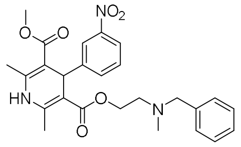	4474	CHEMBL1484	DB00622	2559	55985-32-5	4.28	479.53	13.17	113.69	3	3	**B1**,[22] **C1**,[22] **G2**[22]
Nifedipine (**NIF**) 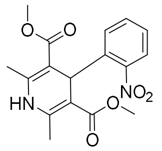	4485	CHEMBL193	DB01115	2514	21829-25-4	2.57	346.34	8.94	110.45	3	3	**B11**,[59] **C1**,[5] **G2**[62]

Sunitinib (**SUN**) 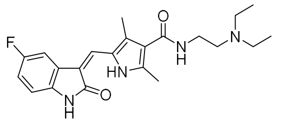	5329102	CHEMBL535	DB01268	5713	557795-19-4	2.57	398.48	11.17	77.23	3	3	**B1**,[63] **C4**,[64] **G2**[63]
Tariquidar (**TAR**) 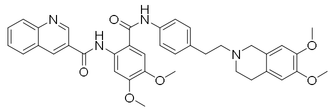	148201	CHEMBL348475	DB06240	11787	206873-63-4	5.35	646.74	18.46	111.25	3	3	**B1**,[65] **C10**,[66] **G2**,[65]
Topotecan (**TOP**) 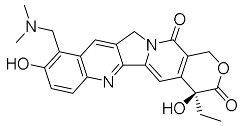	60700	CHEMBL84	DB01030	7101	123948-87-8	2,04	421.45	11.22	103.20	3	3	**B1**,[67] **C4**,[68] **G2**,[67]

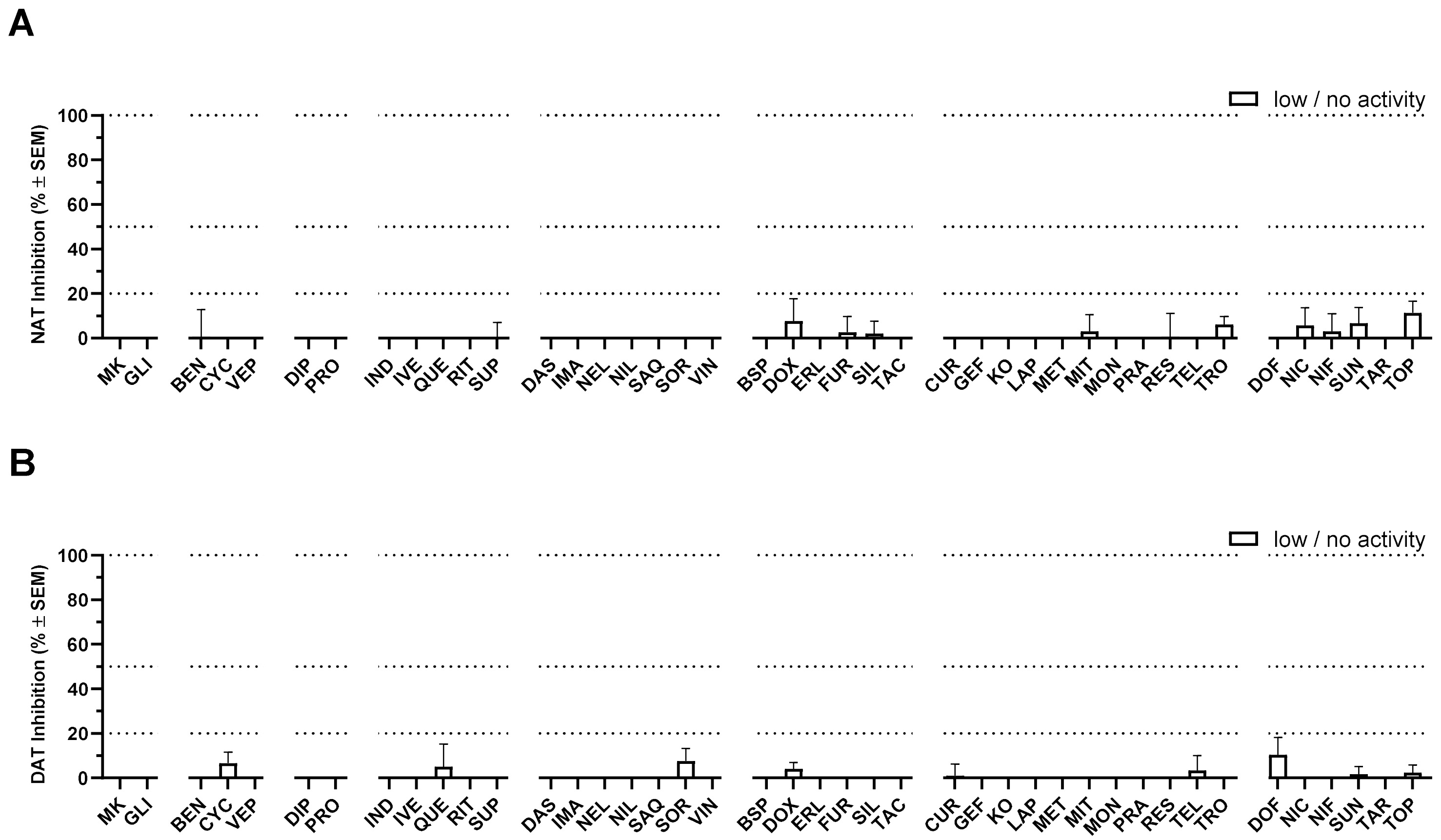

Supplementary Fig. S1. Screening of polypharmacological drugs and drug-like compounds against NAT (*SLC6A2*; **A**) and DAT (*SLC6A3*; **B**). Shown are mean ± standard error of the mean (SEM) values of at least three independent experiments.

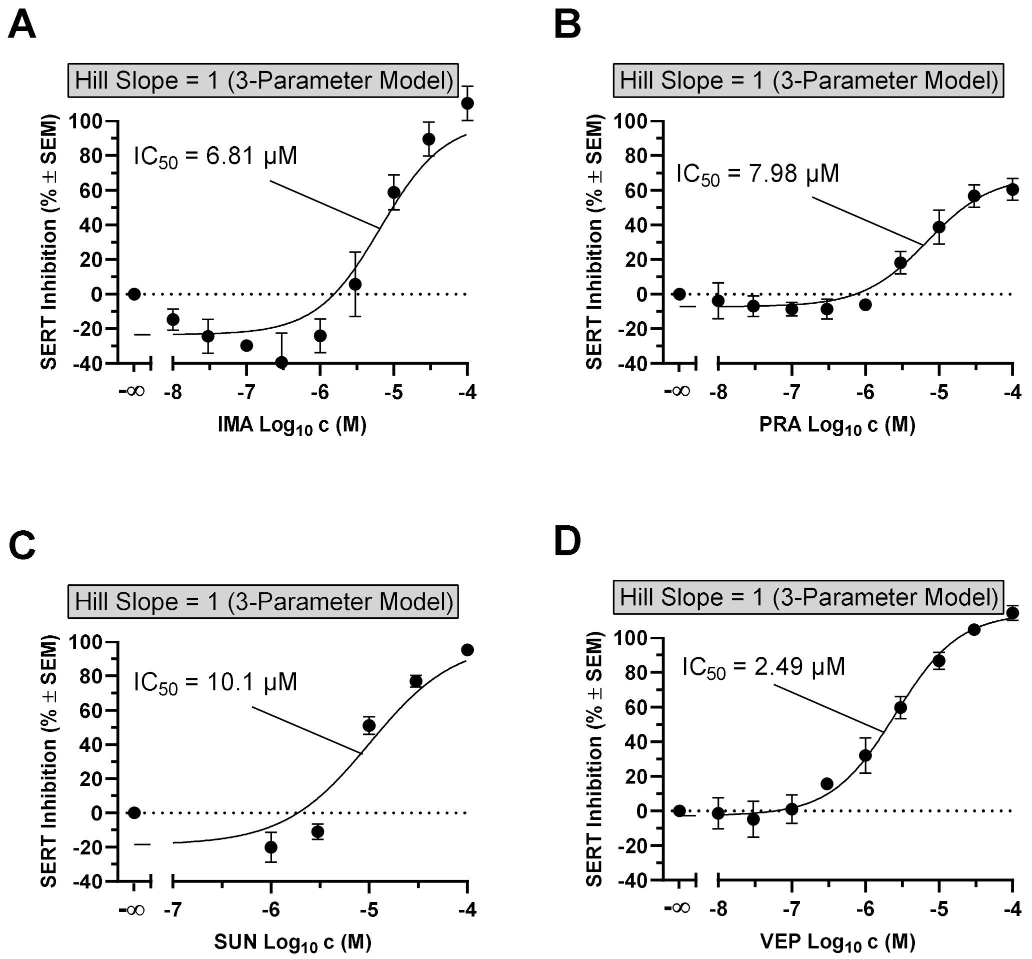

Supplementary Fig. S2. Concentration-dependent effect of hit compounds against SERT with inhibition values in the initial screening of ≥20% + standard error or the mean (SEM); **A** imatinib (**IMA**; IC_{50} = 6.81 µM ± 1.27 µM; r^2 = 0.821); **B** pranlukast (**PRA**; IC_{50} = 7.98 µM ± 1.66 µM; I_{max} = 69.9% ± 5.2%; r^2 = 0.817); **C** sunitinib (**SUN**; IC_{50} = 10.1 µM ± 0.6 µM; r^2 = 0.881); **D** verapamil (**VEP**; IC_{50} = 2.49 µM ± 0.47 µM; I_{max} = 116% ± 4%; r^2 = 0.925). Data shown as mean ± SEM of at least three independent experiments.

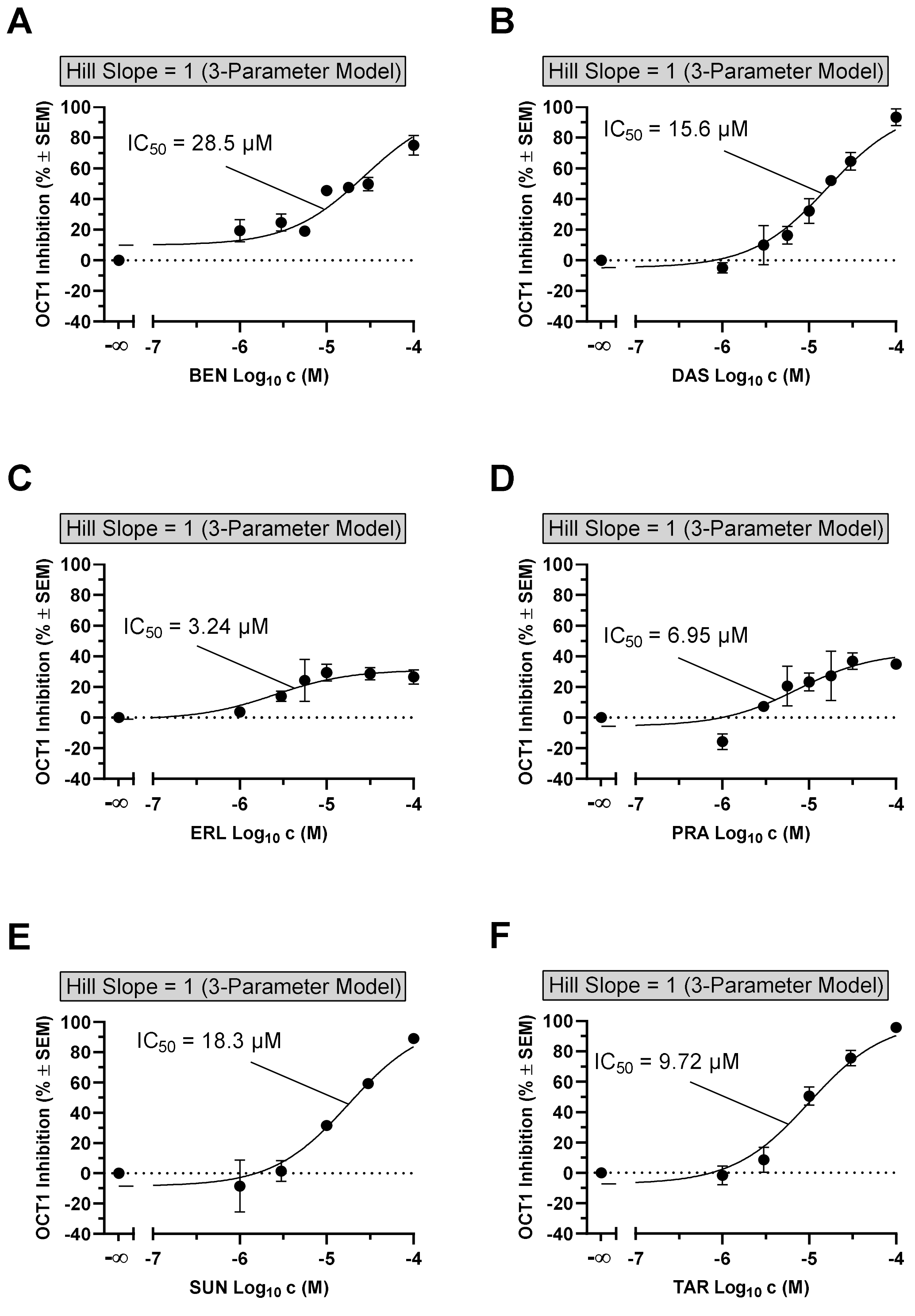
A
Hill Slope = 1 (3-Parameter Model)
IC50 = 28.5 µM
OCT1 Inhibition (% ± SEM)
BEN Log10 c (M)
B
Hill Slope = 1 (3-Parameter Model)
IC50 = 15.6 µM
OCT1 Inhibition (% ± SEM)
DAS Log10 c (M)
C
Hill Slope = 1 (3-Parameter Model)
IC50 = 3.24 µM
OCT1 Inhibition (% ± SEM)
ERL Log10 c (M)
D
Hill Slope = 1 (3-Parameter Model)
IC50 = 6.95 µM
OCT1 Inhibition (% ± SEM)
PRA Log10 c (M)
E
Hill Slope = 1 (3-Parameter Model)
IC50 = 18.3 µM
OCT1 Inhibition (% ± SEM)
SUN Log10 c (M)
F
Hill Slope = 1 (3-Parameter Model)
IC50 = 9.72 µM
OCT1 Inhibition (% ± SEM)
TAR Log10 c (M)

G

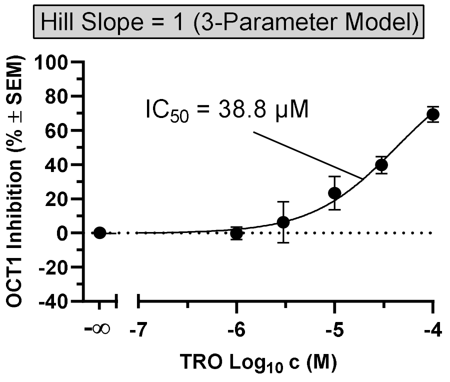

H

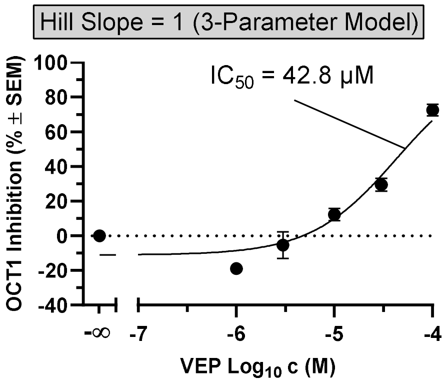

Supplementary Fig. S3. Concentration-dependent effect of hit compounds against OCT1 with inhibition values in the initial screening of ≥20% SEM; **A** benzbromarone (**BEN**; IC_{50} = 28.5 μM ± 3.8 μM; r^2 = 0.803); **B** dasatinib (**DAS**; IC_{50} = 15.6 μM ± 2.9 μM; r^2 = 0.849); **C** erlotinib (**ERL**; IC_{50} = 3.24 μM ± 0.52 μM; I_{max} = 32.5% ± 3.8%; r^2 = 0.818; **D** pranlukast (**PRA**; IC_{50} = 6.95 μM ± 1.32 μM; I_{max} = 41.6% ± 5.0%; r^2 = 0.841); **E** sunitinib (**SUN**; IC_{50} = 18.3 μM ± 1.0 μM); r^2 = 0.856); **F** tariquidar (**TAR**; IC_{50} = 9.71 μM ± 0.72 μM; r^2 = 0.912); **G** troglitazone (**TRO**; IC_{50} = 38.8 μM ± 2.0 μM; r^2 = 0.800); **H** verapamil (**VEP**; IC_{50} = 42.8 μM ± 2.0 μM; r^2 = 0.908) Data shown as mean ± SEM of at least three independent experiments.

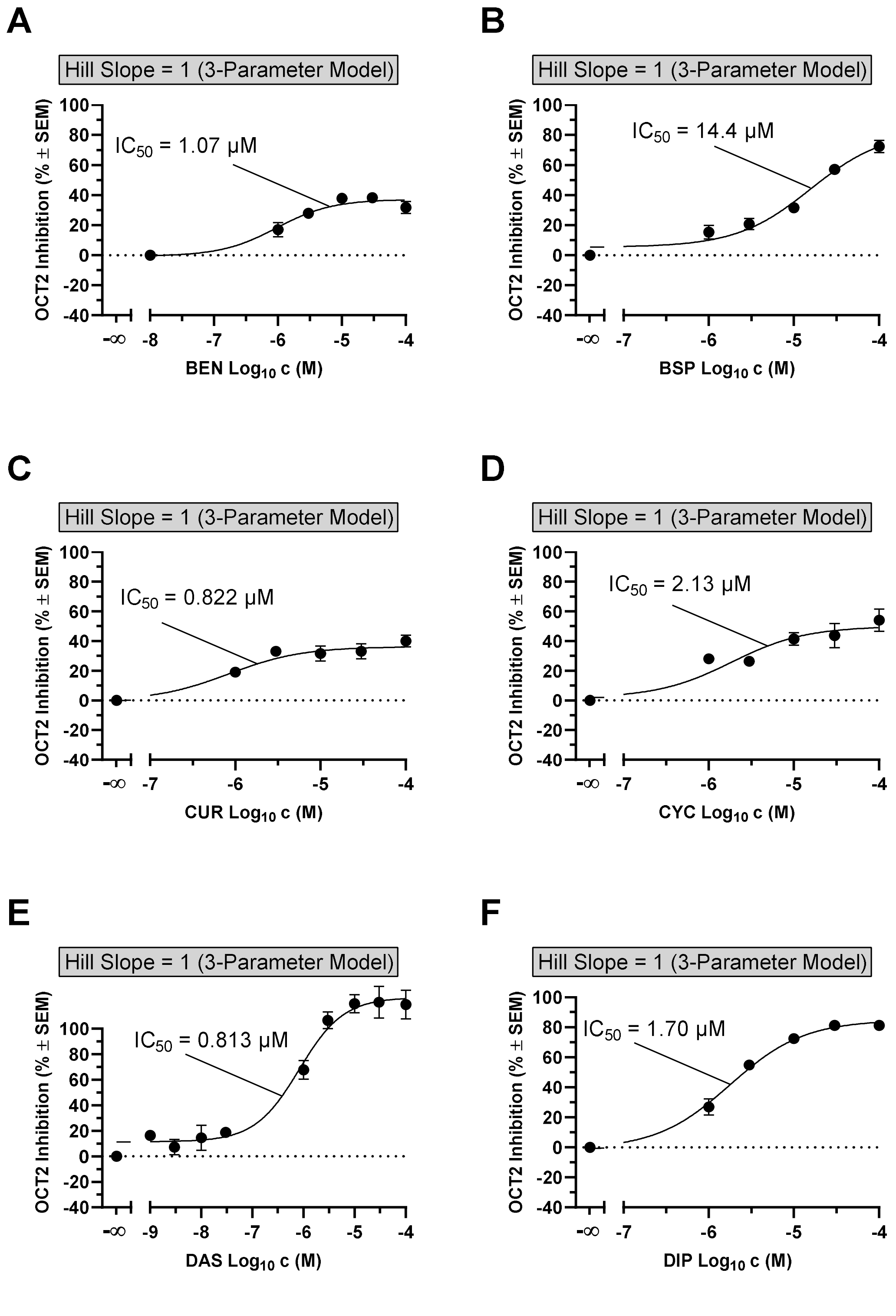
A
Hill Slope = 1 (3-Parameter Model)
IC50 = 1.07 µM
OCT2 Inhibition (% ± SEM)
BEN Log10 c (M)
B
Hill Slope = 1 (3-Parameter Model)
IC50 = 14.4 µM
OCT2 Inhibition (% ± SEM)
BSP Log10 c (M)
C
Hill Slope = 1 (3-Parameter Model)
IC50 = 0.822 µM
OCT2 Inhibition (% ± SEM)
CUR Log10 c (M)
D
Hill Slope = 1 (3-Parameter Model)
IC50 = 2.13 µM
OCT2 Inhibition (% ± SEM)
CYC Log10 c (M)
E
Hill Slope = 1 (3-Parameter Model)
IC50 = 0.813 µM
OCT2 Inhibition (% ± SEM)
DAS Log10 c (M)
F
Hill Slope = 1 (3-Parameter Model)
IC50 = 1.70 µM
OCT2 Inhibition (% ± SEM)
DIP Log10 c (M)

G

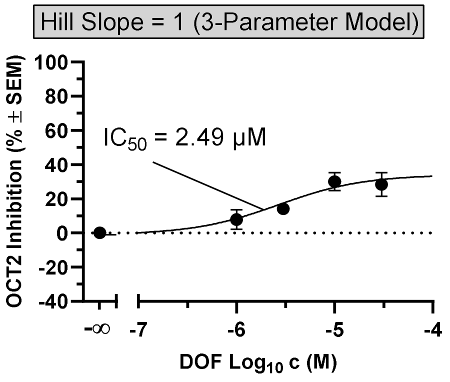
Hill Slope = 1 (3-Parameter Model)
IC50 = 2.49 μM
OCT2 Inhibition (% ± SEM)
100
80
60
40
20
0
-20
-40
-∞
-7
-6
-5
-4
DOF Log10 c (M)

H

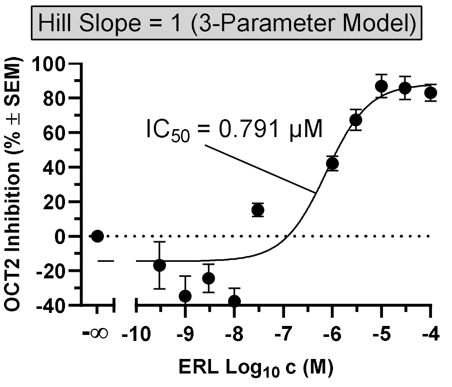
Hill Slope = 1 (3-Parameter Model)
IC50 = 0.791 μM
OCT2 Inhibition (% ± SEM)
100
80
60
40
20
0
-20
-40
-∞
-10
-9
-8
-7
-6
-5
-4
ERL Log10 c (M)

I

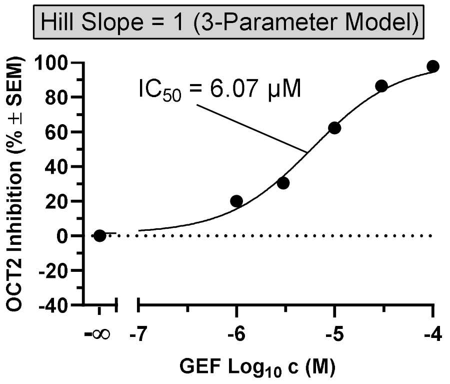
Hill Slope = 1 (3-Parameter Model)
IC50 = 6.07 μM
OCT2 Inhibition (% ± SEM)
100
80
60
40
20
0
-20
-40
-∞
-7
-6
-5
-4
GEF Log10 c (M)

J

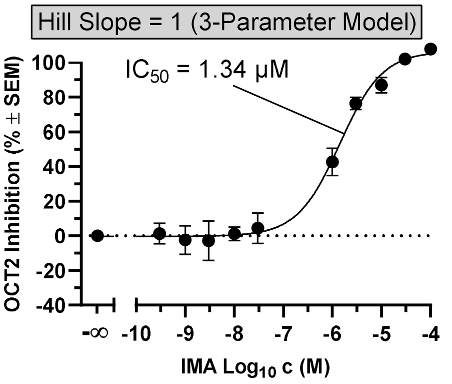
Hill Slope = 1 (3-Parameter Model)
IC50 = 1.34 μM
OCT2 Inhibition (% ± SEM)
100
80
60
40
20
0
-20
-40
-∞
-10
-9
-8
-7
-6
-5
-4
IMA Log10 c (M)

K

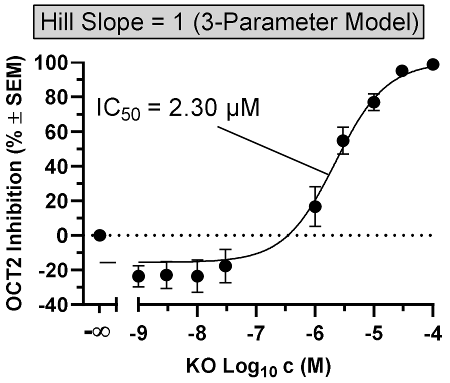
Hill Slope = 1 (3-Parameter Model)
IC50 = 2.30 μM
OCT2 Inhibition (% ± SEM)
100
80
60
40
20
0
-20
-40
-∞
-9
-8
-7
-6
-5
-4
KO Log10 c (M)

L

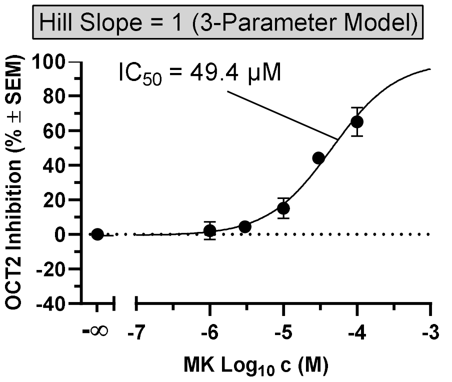
Hill Slope = 1 (3-Parameter Model)
IC50 = 49.4 μM
OCT2 Inhibition (% ± SEM)
100
80
60
40
20
0
-20
-40
-∞
-7
-6
-5
-4
-3
MK Log10 c (M)

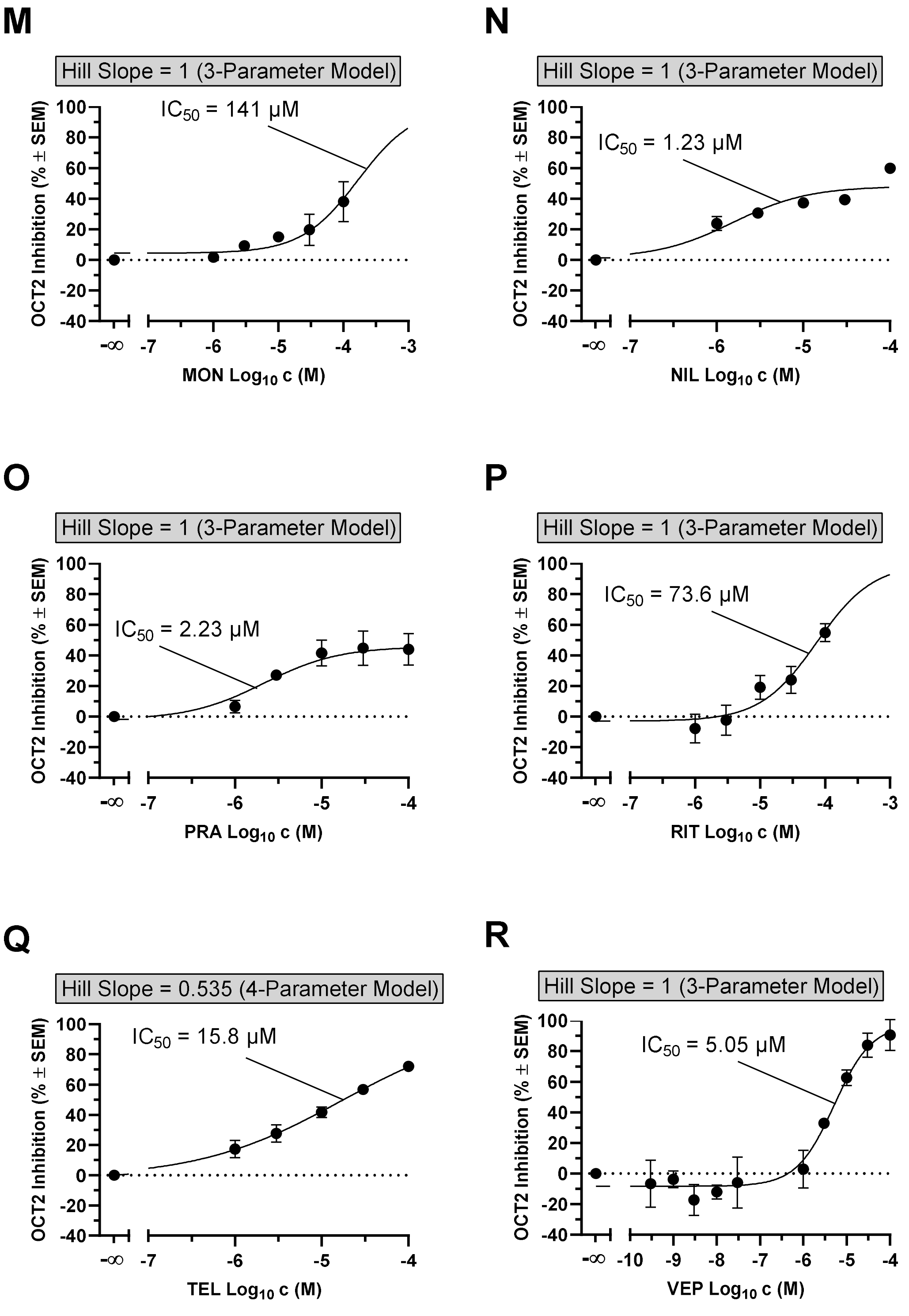
M
Hill Slope = 1 (3-Parameter Model)
IC50 = 141 μM
OCT2 Inhibition (% ± SEM)
MON Log10 c (M)
N
Hill Slope = 1 (3-Parameter Model)
IC50 = 1.23 μM
OCT2 Inhibition (% ± SEM)
NIL Log10 c (M)
O
Hill Slope = 1 (3-Parameter Model)
IC50 = 2.23 μM
OCT2 Inhibition (% ± SEM)
PRA Log10 c (M)
P
Hill Slope = 1 (3-Parameter Model)
IC50 = 73.6 μM
OCT2 Inhibition (% ± SEM)
RIT Log10 c (M)
Q
Hill Slope = 0.535 (4-Parameter Model)
IC50 = 15.8 μM
OCT2 Inhibition (% ± SEM)
TEL Log10 c (M)
R
Hill Slope = 1 (3-Parameter Model)
IC50 = 5.05 μM
OCT2 Inhibition (% ± SEM)
VEP Log10 c (M)

Supplementary Fig. S4. Concentration-dependent effect of hit compounds against OCT2 with inhibition values in the initial screening of ≥20% SEM; **A** benzbromarone (**BEN**; IC_{50} = 1.07 µM ± 0.11 µM; I_{max} = 37.5% ± 2.3%; r^2 = 0.807); **B** bromosulfophthaleine (**BSP**; IC_{50} = 14.4 µM ± 2.6 µM; I_{max} = 83.7% ± 6.7%; r^2 = 0.925); **C** curcimin (**CUR**; IC_{50} = 0.822 µM ± 0.110 µM, I_{max} = 35.7% ± 3.6%; r^2 = 0.863); **D** cyclosporine A (**CYC**; IC_{50} = 2.13 µM ± 0.44 µM; I_{max} = 50.9% ± 5.8%; r^2 = 0.813); **E** dasatinib (**DAS**; IC_{50} = 0.813 µM ± 0.033 µM; I_{max} = 124% ± 9%; r^2 = 0.925); **F** dipyridamole (**DIP**; IC_{50} = 1.70 µM ± 0.25 µM; I_{max} = 84.9% ± 2.7%; r^2 = 0.971); **G** dofequidar (**DOF**; IC_{50} = 2.49 µM ± 0.45 µM; I_{max} = 35.9% ± 4.9%; r^2 = 0.841); **H** erlotinib (**ERL**; IC_{50} = 0.791 µM ± 0.054 µM; I_{max} = 88.2% ± 6.0%; r^2 = 0.867); **I** gefitinib (**GEF**; IC_{50} = 6.07 µM ± 0.40 µM; r^2 = 0.979); **J** imatinib (**IMA**; IC_{50} = 1.34 µM ± 0.21 µM; I_{max} = 107% ± 2%; r^2 = 0.930); **K** Ko143 (**KO**; IC_{50} = 2.30 µM ± 0.39 µM; r^2 = 0.893); **L** MK-571 (**MK**; IC_{50} = 49.4 µM ± 7.6 µM; r^2 = 0.882); **M** montelukast (**MON**; IC_{50} = 141 µM ± 26 µM; r^2 = 0.888); **N** nilotinib (**NIL**; IC_{50} = 1.23 µM ± 0.26 µM; I_{max} = 46.5% ± 3.4%; r^2 = 0.825); **O** pranlukast (**PRA**; IC_{50} = 2.23 µM ± 0.37 µM; I_{max} = 46.7% ± 9.8%; r^2 = 0.817); **P** ritonavir (**RIT**; IC_{50} = 73.6 µM ± 9.4 µM; r^2 = 0.846); **Q** telmisartan (**TEL**; IC_{50} = 15.8 µM ± 2.7 µM; slope: 0.535 ± 0.048; r^2 = 0.954); **R** verapamil (**VEP**; IC_{50} = 5.05 µM ± 0.33 µM; I_{max} = 97.1% ± 6.8%; r^2 = 0.874). Data shown as mean ± SEM of at least three independent experiments.

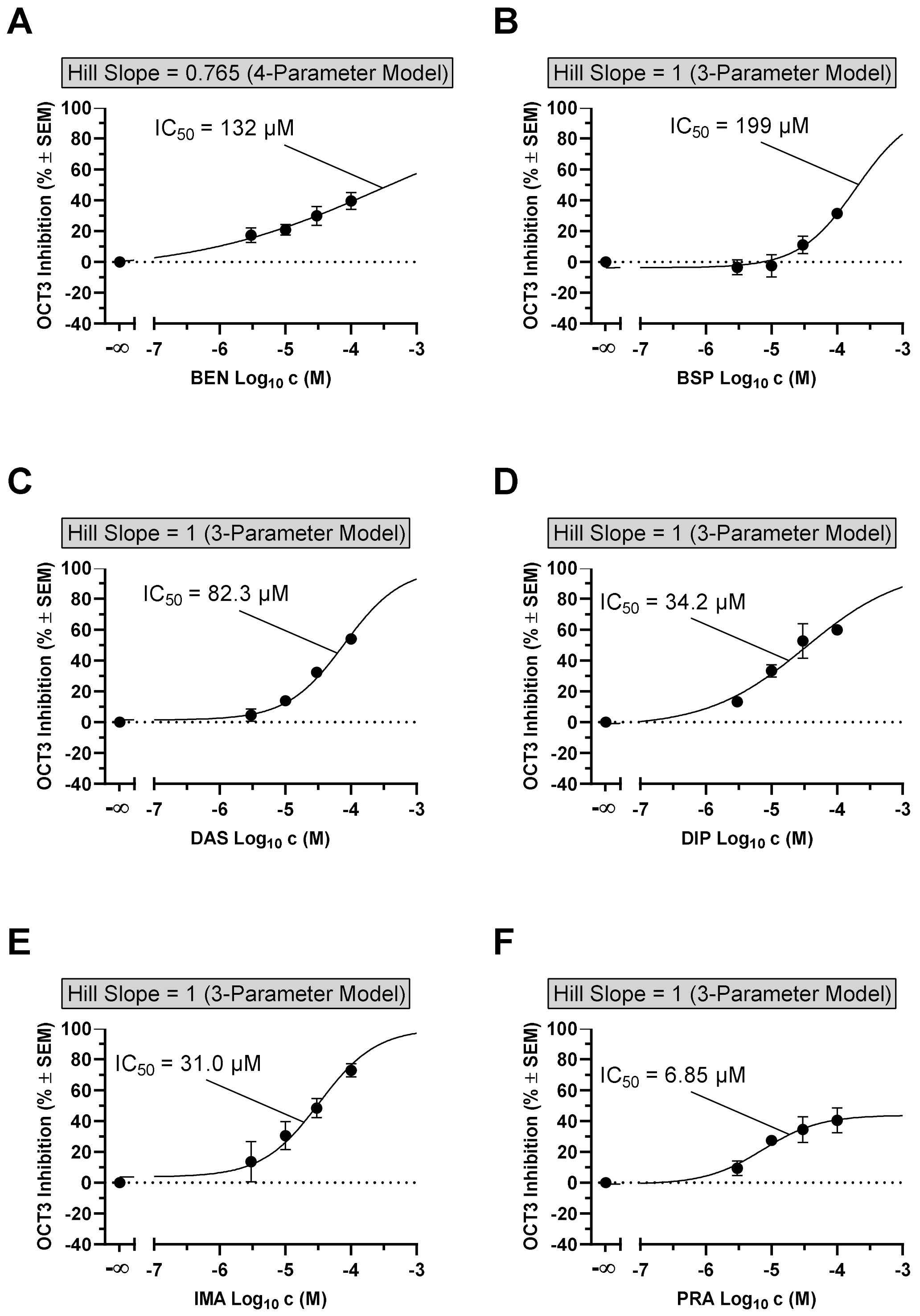
A
Hill Slope = 0.765 (4-Parameter Model)
IC50 = 132 µM
OCT3 Inhibition (% ± SEM)
BEN Log10 c (M)
B
Hill Slope = 1 (3-Parameter Model)
IC50 = 199 µM
OCT3 Inhibition (% ± SEM)
BSP Log10 c (M)
C
Hill Slope = 1 (3-Parameter Model)
IC50 = 82.3 µM
OCT3 Inhibition (% ± SEM)
DAS Log10 c (M)
D
Hill Slope = 1 (3-Parameter Model)
IC50 = 34.2 µM
OCT3 Inhibition (% ± SEM)
DIP Log10 c (M)
E
Hill Slope = 1 (3-Parameter Model)
IC50 = 31.0 µM
OCT3 Inhibition (% ± SEM)
IMA Log10 c (M)
F
Hill Slope = 1 (3-Parameter Model)
IC50 = 6.85 µM
OCT3 Inhibition (% ± SEM)
PRA Log10 c (M)

G

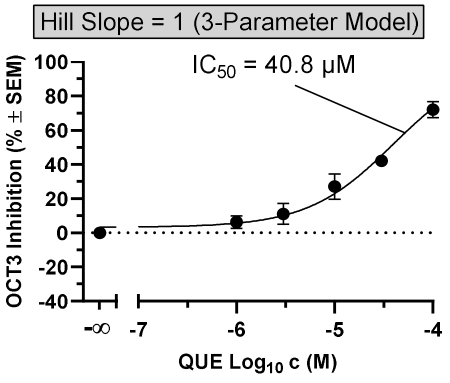
Hill Slope = 1 (3-Parameter Model)
IC50 = 40.8 µM
OCT3 Inhibition (% ± SEM)
100
80
60
40
20
0
-20
-40
-∞
-7
-6
-5
-4
QUE Log10 c (M)

H

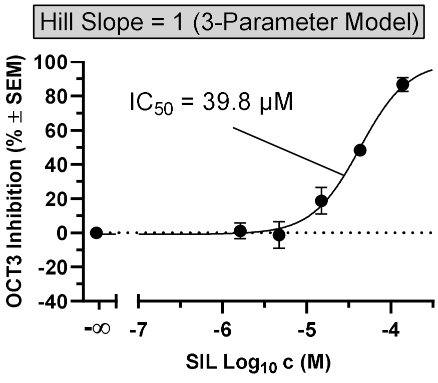
Hill Slope = 1 (3-Parameter Model)
IC50 = 39.8 µM
OCT3 Inhibition (% ± SEM)
100
80
60
40
20
0
-20
-40
-∞
-7
-6
-5
-4
SIL Log10 c (M)

I

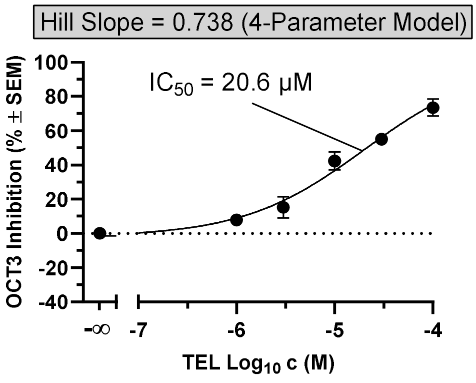
Hill Slope = 0.738 (4-Parameter Model)
IC50 = 20.6 µM
OCT3 Inhibition (% ± SEM)
100
80
60
40
20
0
-20
-40
-∞
-7
-6
-5
-4
TEL Log10 c (M)

J

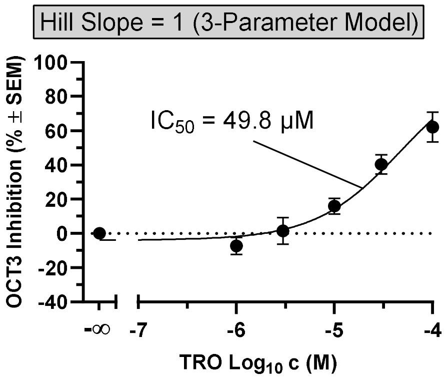
Hill Slope = 1 (3-Parameter Model)
IC50 = 49.8 µM
OCT3 Inhibition (% ± SEM)
100
80
60
40
20
0
-20
-40
-∞
-7
-6
-5
-4
TRO Log10 c (M)

K

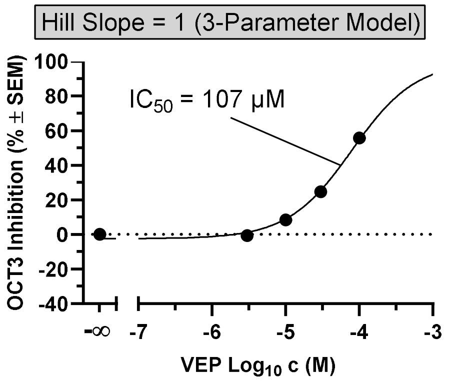
Hill Slope = 1 (3-Parameter Model)
IC50 = 107 µM
OCT3 Inhibition (% ± SEM)
100
80
60
40
20
0
-20
-40
-∞
-7
-6
-5
-4
-3
VEP Log10 c (M)

Supplementary Fig. S5. Concentration-dependent effect of hit compounds against OCT3 with inhibition values in the initial screening of ≥20% SEM; **A** benzbromarone (**BEN**; IC_{50} = 132 μM ± 33 μM; slope: 0.765 ± 0.118; r^2 = 0.949); **B** bromosulfophthaleine (**BSP**; IC_{50} = 199 μM ± 17 μM; r^2 = 0.967); **C** dasatinib (**DAS**; IC_{50} = 82.3 μM ± 6.3 μM; r^2 = 0.956); **D** dipyridamole (**DIP**; IC_{50} = 34.2 μM ± 3.8 μM; r^2 = 0.834); **E** imatinib (**IMA**; IC_{50} = 31.0 μM ± 4.3 μM; r^2 = 0.829); **F** pranlukast (**PRA**; IC_{50} = 6.85 μM ± 1.04 μM; I_{max} = 44.0% ± 7.7%; r^2 = 0.814); **G** quercetin (**QUE**; IC_{50} = 40.8 μM ± 4.8 μM; r^2 = 0.867); **H** silymarin (**SIL**; IC_{50} = 39.8 μM ± 4.2 μM; r^2 = 0.871); **I** telmisartan (**TEL**; IC_{50} = 20.6 μM ± 3.0 μM; slope: 0.738 ± 0.102; r^2 = 0.914); **J** troglitazone (**TRO**; IC_{50} = 49.8 μM ± 7.6 μM; r^2 = 0.832); **K** verapamil (**VEP**; IC_{50} = 107 μM ± 13 μM; r^2 = 0.984). Data shown as mean ± SEM of at least three independent experiments.

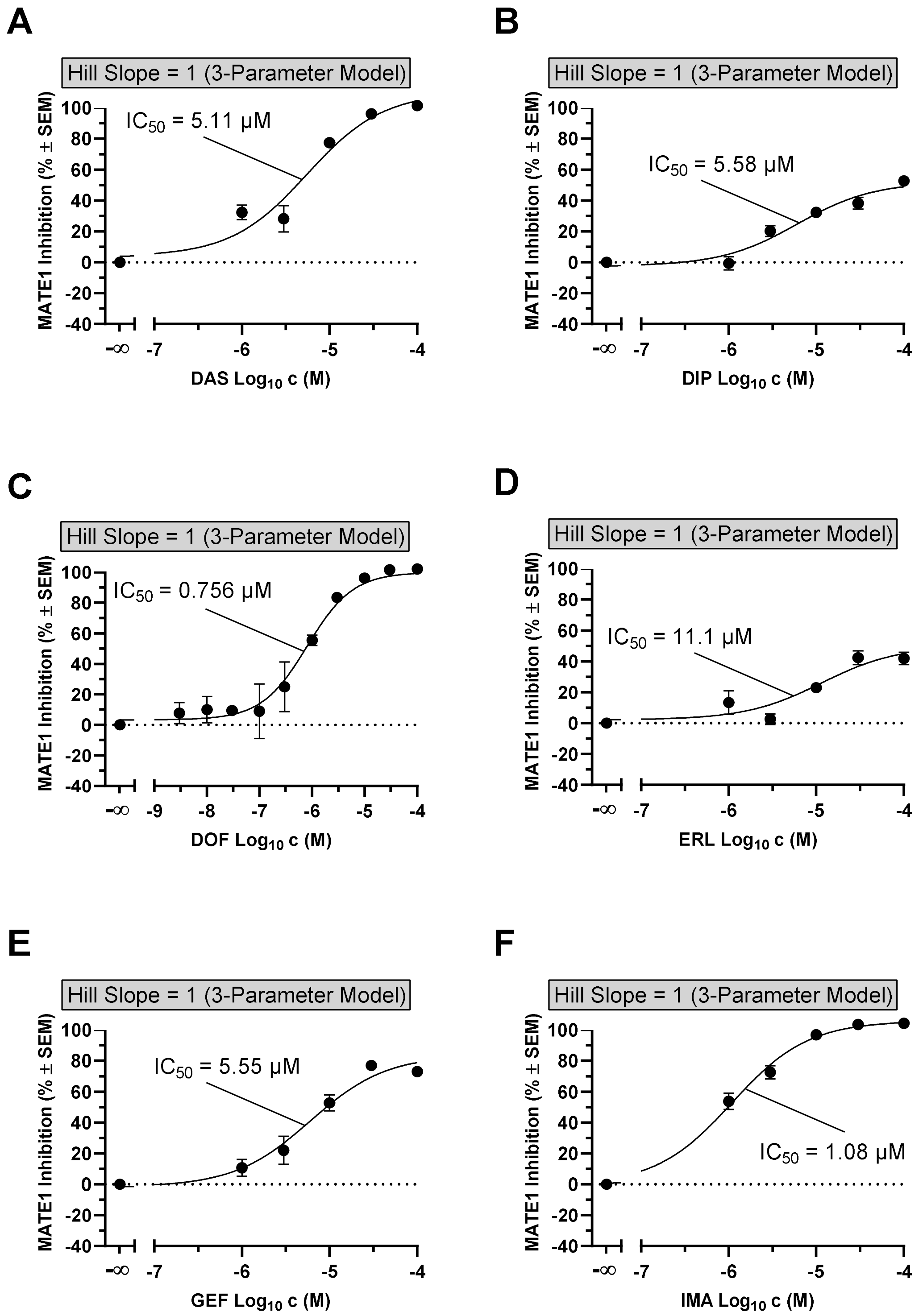
A
Hill Slope = 1 (3-Parameter Model)
IC50 = 5.11 μM
MATE1 Inhibition (% ± SEM)
100
80
60
40
20
0
-20
-40
-∞
-7
-6
-5
-4
DAS Log10 c (M)
B
Hill Slope = 1 (3-Parameter Model)
IC50 = 5.58 μM
MATE1 Inhibition (% ± SEM)
DIP Log10 c (M)
C
Hill Slope = 1 (3-Parameter Model)
IC50 = 0.756 μM
MATE1 Inhibition (% ± SEM)
-∞
-9
-8
-7
-6
-5
-4
DOF Log10 c (M)
D
Hill Slope = 1 (3-Parameter Model)
IC50 = 11.1 μM
MATE1 Inhibition (% ± SEM)
ERL Log10 c (M)
E
Hill Slope = 1 (3-Parameter Model)
IC50 = 5.55 μM
MATE1 Inhibition (% ± SEM)
GEF Log10 c (M)
F
Hill Slope = 1 (3-Parameter Model)
IC50 = 1.08 μM
MATE1 Inhibition (% ± SEM)
IMA Log10 c (M)

G

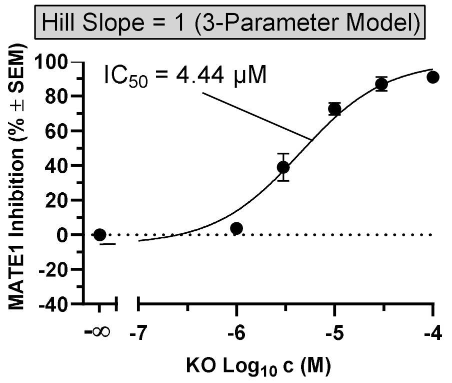
Hill Slope = 1 (3-Parameter Model)
IC_{50} = 4.44 μM
MATE1 Inhibition (% ± SEM)
KO Log_{10} c (M)

H

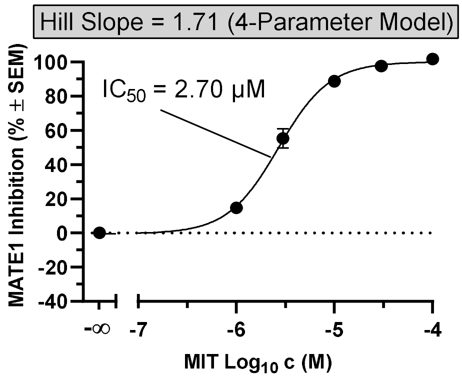
Hill Slope = 1.71 (4-Parameter Model)
IC_{50} = 2.70 μM
MATE1 Inhibition (% ± SEM)
MIT Log_{10} c (M)

I

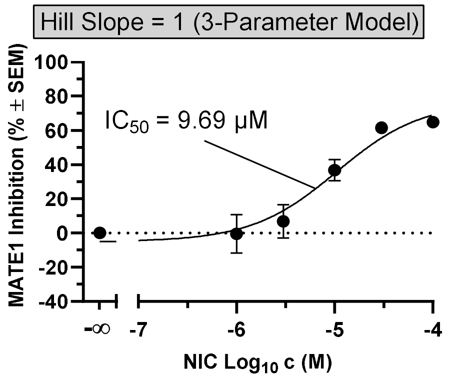
Hill Slope = 1 (3-Parameter Model)
IC_{50} = 9.69 μM
MATE1 Inhibition (% ± SEM)
NIC Log_{10} c (M)

J

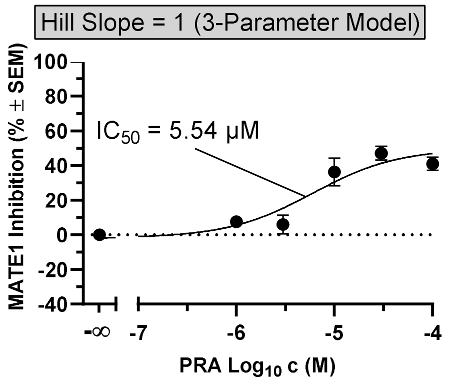
Hill Slope = 1 (3-Parameter Model)
IC_{50} = 5.54 μM
MATE1 Inhibition (% ± SEM)
PRA Log_{10} c (M)

K

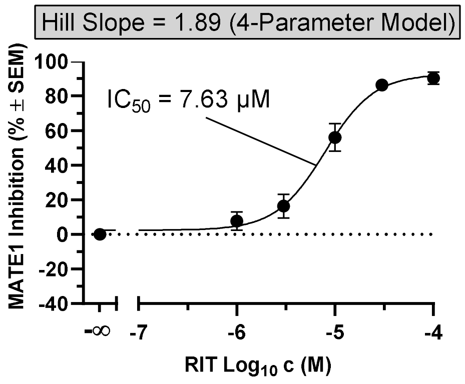
Hill Slope = 1.89 (4-Parameter Model)
IC_{50} = 7.63 μM
MATE1 Inhibition (% ± SEM)
RIT Log_{10} c (M)

L

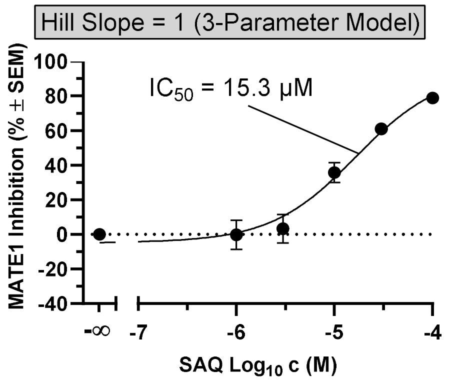
Hill Slope = 1 (3-Parameter Model)
IC_{50} = 15.3 μM
MATE1 Inhibition (% ± SEM)
SAQ Log_{10} c (M)

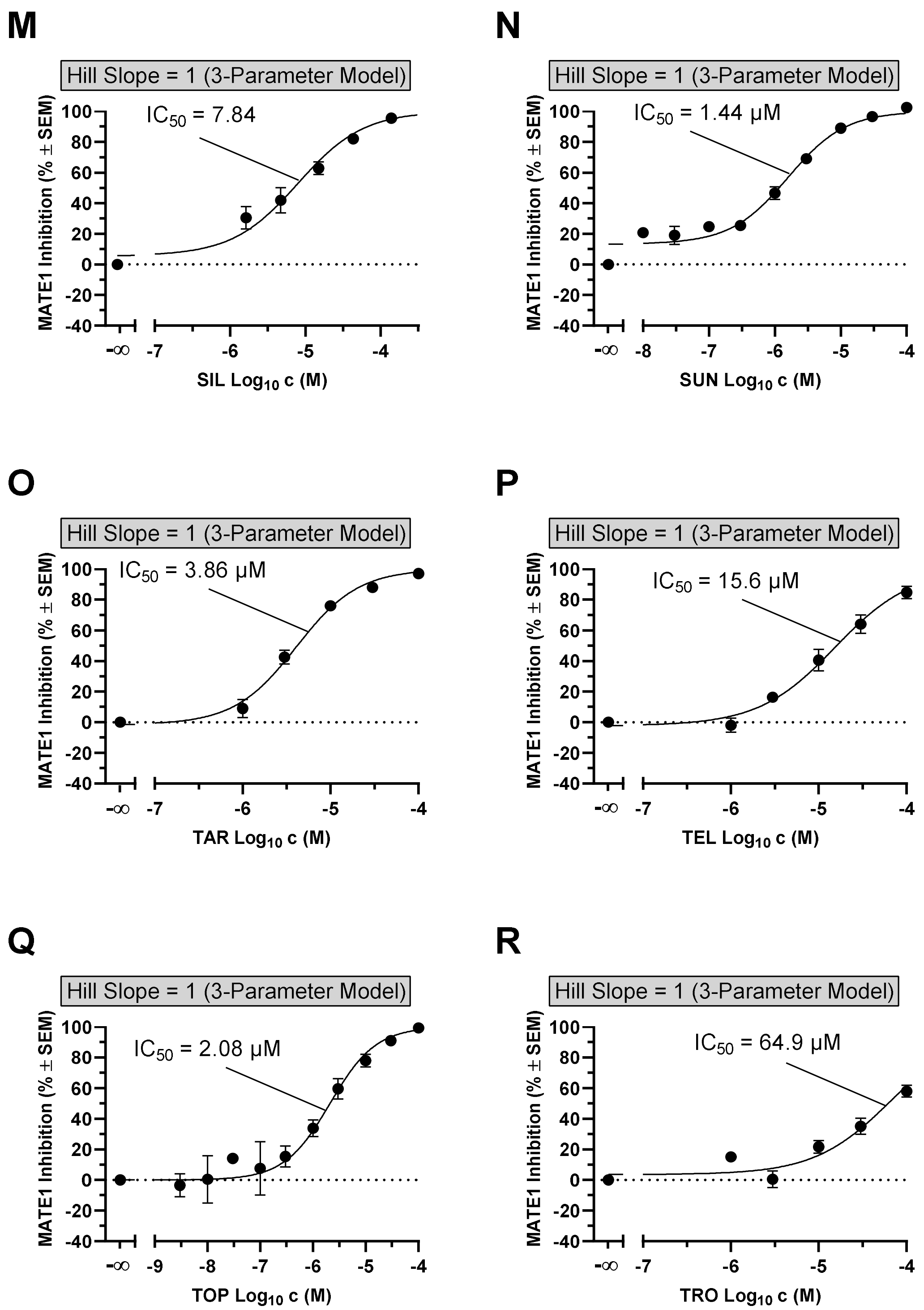
M
Hill Slope = 1 (3-Parameter Model)
IC50 = 7.84
MATE1 Inhibition (% ± SEM)
SIL Log10 c (M)
N
Hill Slope = 1 (3-Parameter Model)
IC50 = 1.44 μM
MATE1 Inhibition (% ± SEM)
SUN Log10 c (M)
O
Hill Slope = 1 (3-Parameter Model)
IC50 = 3.86 μM
MATE1 Inhibition (% ± SEM)
TAR Log10 c (M)
P
Hill Slope = 1 (3-Parameter Model)
IC50 = 15.6 μM
MATE1 Inhibition (% ± SEM)
TEL Log10 c (M)
Q
Hill Slope = 1 (3-Parameter Model)
IC50 = 2.08 μM
MATE1 Inhibition (% ± SEM)
TOP Log10 c (M)
R
Hill Slope = 1 (3-Parameter Model)
IC50 = 64.9 μM
MATE1 Inhibition (% ± SEM)
TRO Log10 c (M)

S

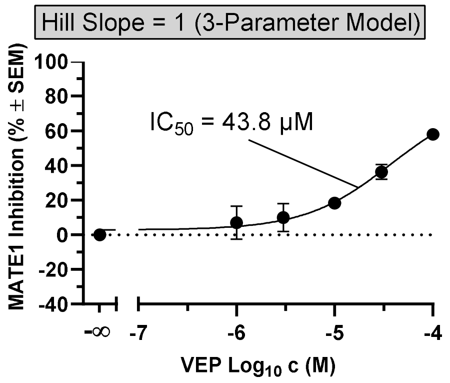

Supplementary Fig. S6. Concentration-dependent effect of hit compounds against MATE1 with inhibition values in the initial screening of ≥20% SEM; **A** dasatinib (**DAS**; IC_{50} = 5.11 μM ± 0.84 μM; I_{max} = 110% ± 3%; r^2 = 0.925); **B** dipyridamole (**DIP**; IC_{50} = 5.58 μM ± 0.68 μM; I_{max} = 52.0% ± 2.0%; r^2 = 0.890); **C** dofequidar (**DOF**; IC_{50} = 0.756 μM ± 0.065 μM; r^2 = 0.935); **D** erlotinib (**ERL**; IC_{50} = 11.1 μM ± 0.7 μM; I_{max} = 49.6% ± 3.0%; r^2 = 0.862); **E** gefitinib (**GEF**; IC_{50} = 5.55 μM ± 1.11 μM; I_{max} = 84.8% ± 4.5%; r^2 = 0.909); **F** imatinib (**IMA**; IC_{50} = 1.08 μM ± 0.14 μM; I_{max} = 106% ± 1%; r^2 = 0.978); **G** Ko143 (**KO**; IC_{50} = 4.44 μM ± 0.49 μM; r^2 = 0.943); **H** mitoxantrone (**MIT**; IC_{50} = 2.70 μM ± 0.19 μM; slope: 1.71 ± 0.04; r^2 = 0.990); **I** nicardipine (**NIC**; IC_{50} = 9.69 μM ± 0.92 μM; I_{max} = 76.4% ± 1.9%; r^2 = 0.828); **J** pranlukast (**PRA**; IC_{50} = 5.54 μM ± 1.05 μM; I_{max} = 50.1% ± 4.4%; r^2 = 0.810); **K** ritonavir (**RIT**; IC_{50} = 7.63 μM ± 0.69 μM; I_{max} = 92.3% ± 1.3%; slope: 1.89 ± 0.30; r^2 = 0.961); **L** saquinavir (**SAQ**; IC_{50} = 15.3 μM ± 2.5 μM; I_{max} = 94.5% ± 3.9%; r^2 = 0.903); **M** silymarin (**SIL**; IC_{50} = 7.84 μM ± 0.79 μM; r^2 = 0.927); **N** sunitinib (**SUN**; IC_{50} = 1.44 μM ± 0.05 μM; r^2 = 0.952); **O** tariquidar (**TAR**; IC_{50} = 3.86 μM ± 0.19 μM; r^2 = 0.982); **P** telmisartan (**TEL**; IC_{50} = 15.6 μM ± 2.5 μM r^2 = 0.937); **Q** topotecan (**TOP**; IC_{50} = 2.08 μM ± 0.34 μM r^2 = 0.931); **R** troglitazone (**TRO**; IC_{50} = 64.9 μM ± 5.4 μM; r^2 = 0.870); **S**

verapamil (**VEP**; IC_{50} = 43.8 μM ± 5.4 μM; I_{max} = 81.6% ± 2.0%; r^2 = 0.857). Data shown as mean ± SEM of at least three independent experiments.

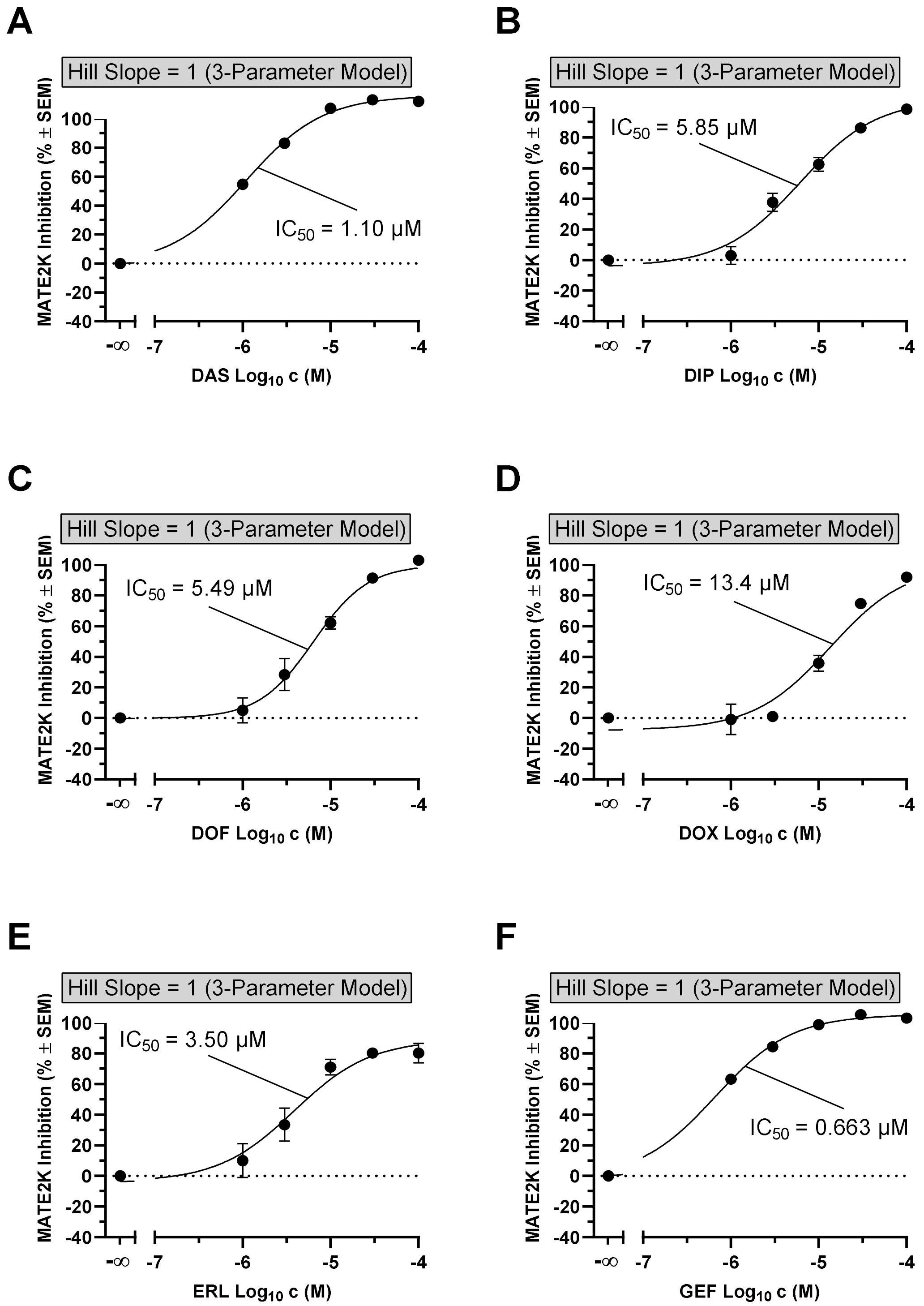
A
Hill Slope = 1 (3-Parameter Model)
IC50 = 1.10 µM
MATE2K Inhibition (% ± SEM)
DAS Log10 c (M)
B
Hill Slope = 1 (3-Parameter Model)
IC50 = 5.85 µM
MATE2K Inhibition (% ± SEM)
DIP Log10 c (M)
C
Hill Slope = 1 (3-Parameter Model)
IC50 = 5.49 µM
MATE2K Inhibition (% ± SEM)
DOF Log10 c (M)
D
Hill Slope = 1 (3-Parameter Model)
IC50 = 13.4 µM
MATE2K Inhibition (% ± SEM)
DOX Log10 c (M)
E
Hill Slope = 1 (3-Parameter Model)
IC50 = 3.50 µM
MATE2K Inhibition (% ± SEM)
ERL Log10 c (M)
F
Hill Slope = 1 (3-Parameter Model)
IC50 = 0.663 µM
MATE2K Inhibition (% ± SEM)
GEF Log10 c (M)

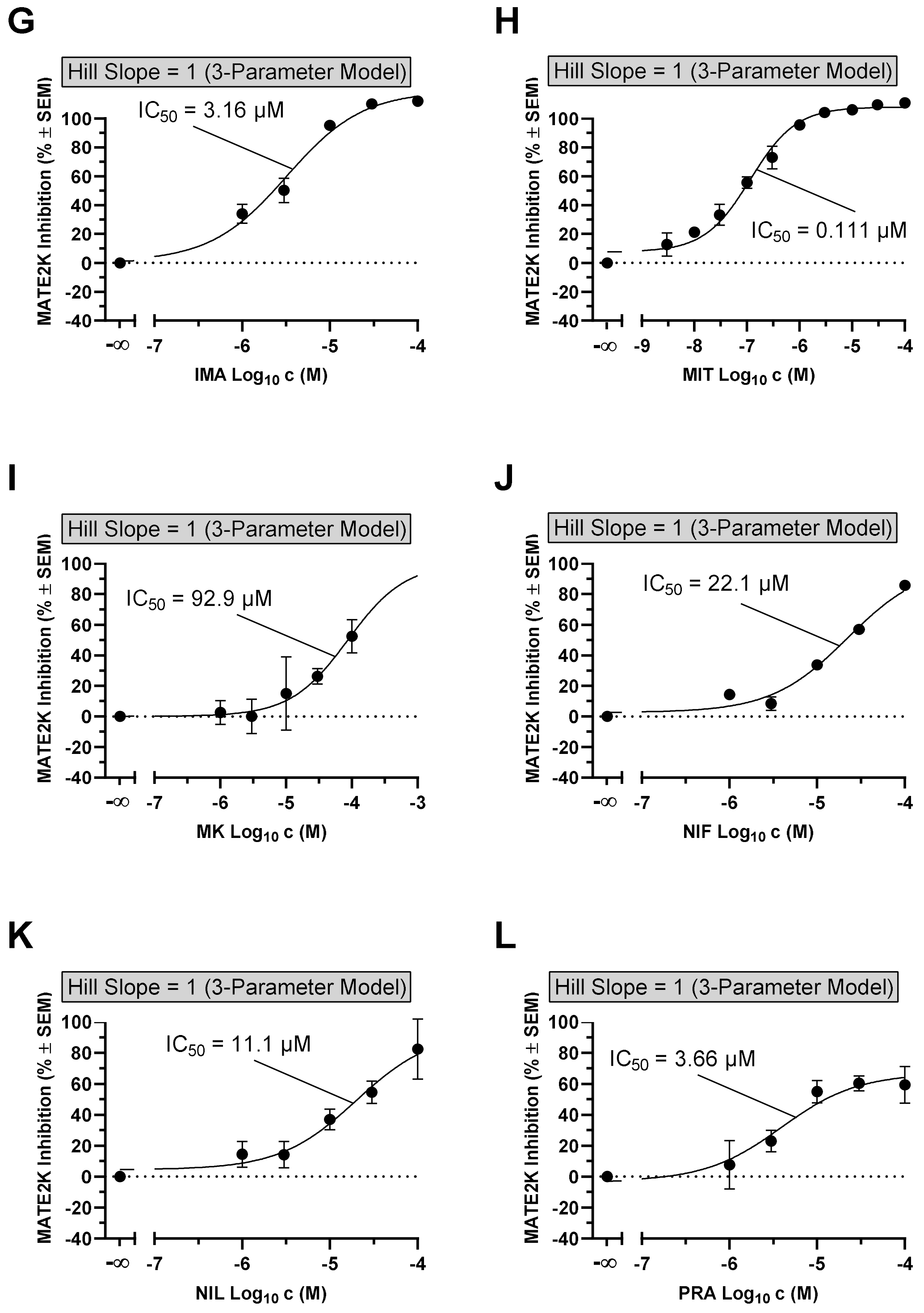
G
Hill Slope = 1 (3-Parameter Model)
IC50 = 3.16 µM
MATE2K Inhibition (% ± SEM)
IMA Log10 c (M)
H
Hill Slope = 1 (3-Parameter Model)
IC50 = 0.111 µM
MATE2K Inhibition (% ± SEM)
MIT Log10 c (M)
I
Hill Slope = 1 (3-Parameter Model)
IC50 = 92.9 µM
MATE2K Inhibition (% ± SEM)
MK Log10 c (M)
J
Hill Slope = 1 (3-Parameter Model)
IC50 = 22.1 µM
MATE2K Inhibition (% ± SEM)
NIF Log10 c (M)
K
Hill Slope = 1 (3-Parameter Model)
IC50 = 11.1 µM
MATE2K Inhibition (% ± SEM)
NIL Log10 c (M)
L
Hill Slope = 1 (3-Parameter Model)
IC50 = 3.66 µM
MATE2K Inhibition (% ± SEM)
PRA Log10 c (M)

M

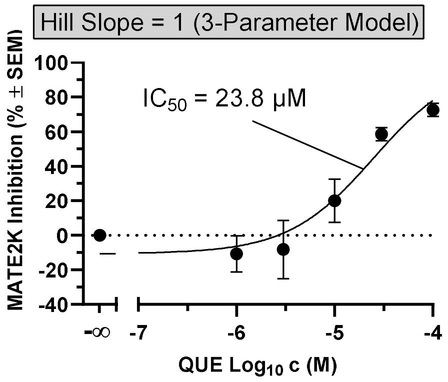
Hill Slope = 1 (3-Parameter Model)
IC50 = 23.8 μM
MATE2K Inhibition (% ± SEM)
QUE Log10 c (M)

N

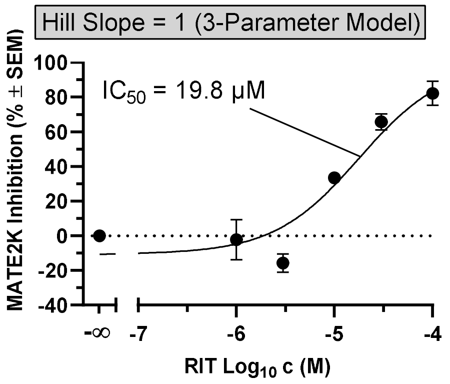
Hill Slope = 1 (3-Parameter Model)
IC50 = 19.8 μM
MATE2K Inhibition (% ± SEM)
RIT Log10 c (M)

O

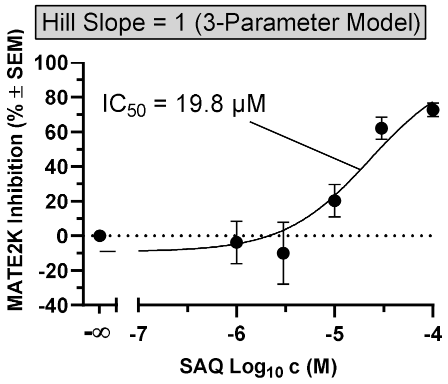
Hill Slope = 1 (3-Parameter Model)
IC50 = 19.8 μM
MATE2K Inhibition (% ± SEM)
SAQ Log10 c (M)

P

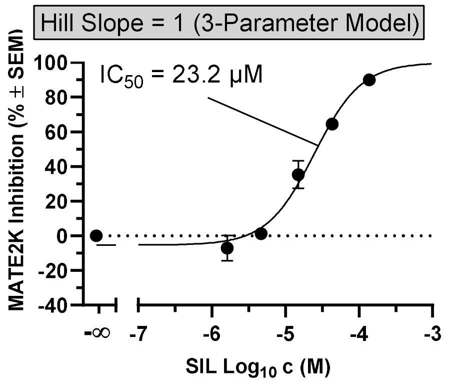
Hill Slope = 1 (3-Parameter Model)
IC50 = 23.2 μM
MATE2K Inhibition (% ± SEM)
SIL Log10 c (M)

Q

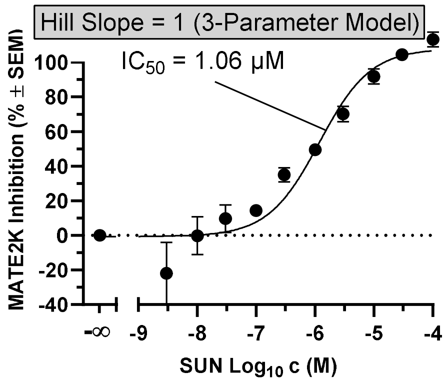
Hill Slope = 1 (3-Parameter Model)
IC50 = 1.06 μM
MATE2K Inhibition (% ± SEM)
SUN Log10 c (M)

R

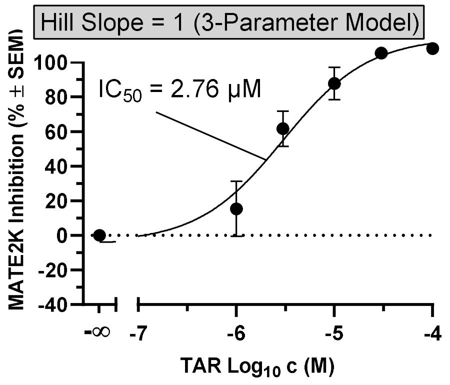
Hill Slope = 1 (3-Parameter Model)
IC50 = 2.76 μM
MATE2K Inhibition (% ± SEM)
TAR Log10 c (M)

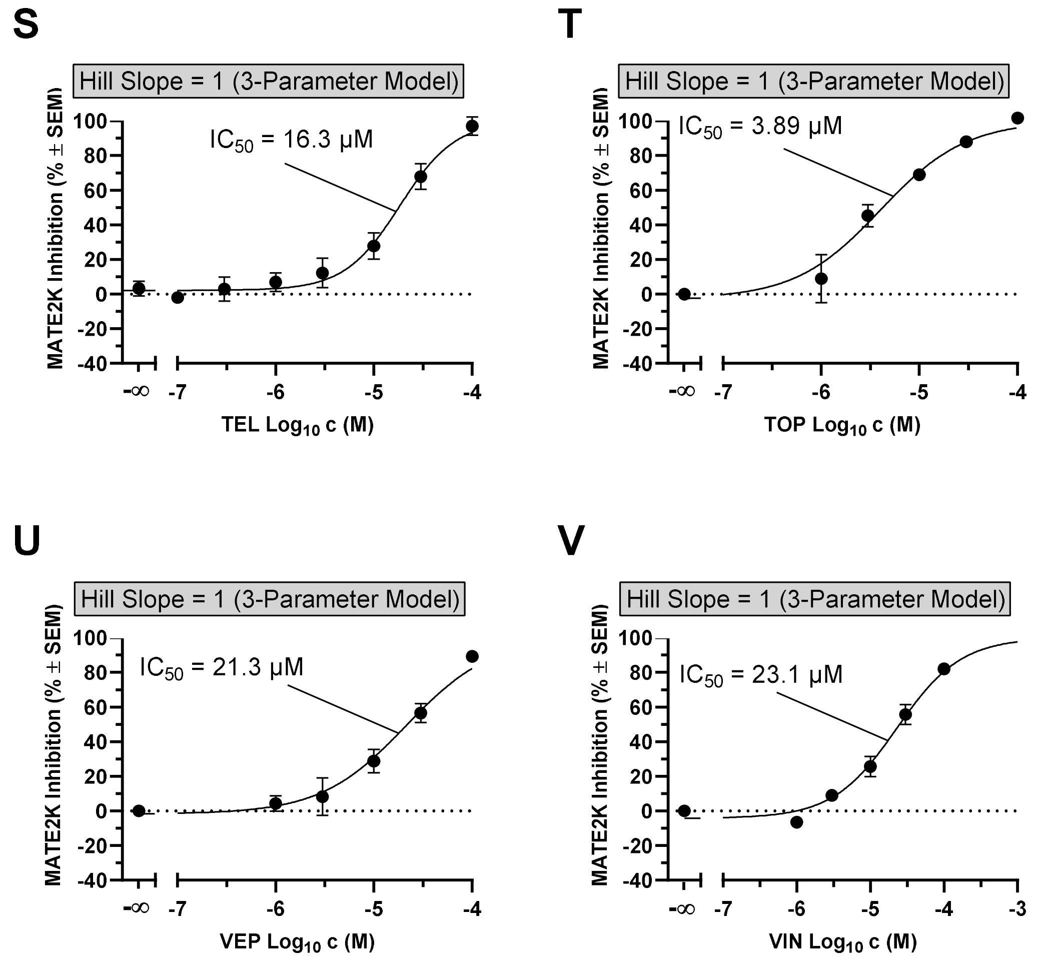

Supplementary Fig. S7. Concentration-dependent effect of hit compounds against MATE2K with inhibition values in the initial screening of ≥20% SEM; **A** dasatinib (**DAS**; IC_{50} = 1.10 µM ± 0.04 µM; I_{max} = 116% ± 1%; r^2 = 0.991); **B** dipyridamole (**DIP**; IC_{50} = 5.85 µM ± 0.63 µM; I_{max} = 105% ± 3%; r^2 = 0.953); **C** dofequidar (**DOF**; IC_{50} = 5.49 µM ± 0.23 µM; r^2 = 0.956); **D** doxorubicin (**DOX**; IC_{50} = 13.4 µM ± 0.5 µM; r^2 = 0.936); **E** erlotinib (**ERL**; IC_{50} = 3.50 µM ± 0.52 µM; I_{max} = 88.5% ± 3.2%; r^2 = 0.854); **F** gefitinib (**GEF**; IC_{50} = 0.663 µM ± 0.035 µM; I_{max} = 105% ± 1%; r^2 = 0.993); **G** imatinib (**IMA**; IC_{50} = 3.16 µM ± 0.39 µM; I_{max} = 119% ± 2%; r^2 = 0.952); **H** mitoxantrone (**MIT**; IC_{50} = 0.111 µM ± 0.021 µM; I_{max} = 107% ± 1%; r^2 = 0.967); **I** MK-571 (**MK**; IC_{50} = 92.9 µM ± 9.7 µM; r^2 =

0.860); **J** nifedipin (**NIF**; IC_{50} = 22.1 μM ± 0.6 μM; r^2 = 0.966); **K** nilotinib (**NIL**; IC_{50} = 11.1 μM ± 1.7 μM; I_{max} = 75.8% ± 8.9%; r^2 = 0.858); **L** pranlukast (**PRA**; IC_{50} = 3.66 μM ± 0.35 μM; I_{max} = 66.2% ± 6.2%; r^2 = 0.807); **M** quercetin (**QUE**; IC_{50} = 23.8 μM ± 1.8 μM; r^2 = 0.844); **N** ritonavir (**RIT**; IC_{50} = 19.8 μM ± 3.2 μM; r^2 = 0.846); **O** saquinavir (**SAQ**; IC_{50} = 19.8 μM ± 4.2 μM; I_{max} = 97.3% ± 6.2%; r^2 = 0.824); **P** silymarin (**SIL**; IC_{50} = 23.2 μM ± 1.2 μM; r^2 = 0.934); **Q** sunitinib (**SUN**; IC_{50} = 1.06 μM ± 0.16 μM; I_{max} = 107% ± 5%; r^2 = 0.922); **R** tariquidar (**TAR**; IC_{50} = 2.76 μM ± 0.35 μM; I_{max} = 114% ± 4%; r^2 = 0.907); **S** telmisartan (**TEL**; IC_{50} = 16.3 μM ± 2.7 μM; r^2 = 0.848); **T** topotecan (**TOP**; IC_{50} = 3.89 μM ± 0.33 μM; r^2 = 0.959). **U** verapamil (**VEP**; IC_{50} = 21.3 μM ± 2.5 μM; r^2 = 0.894); **V** vincristine (**VIN**; IC_{50} = 23.1 μM ± 0.7 μM; r^2 = 0.965). Data shown as mean ± SEM of at least three independent experiments.

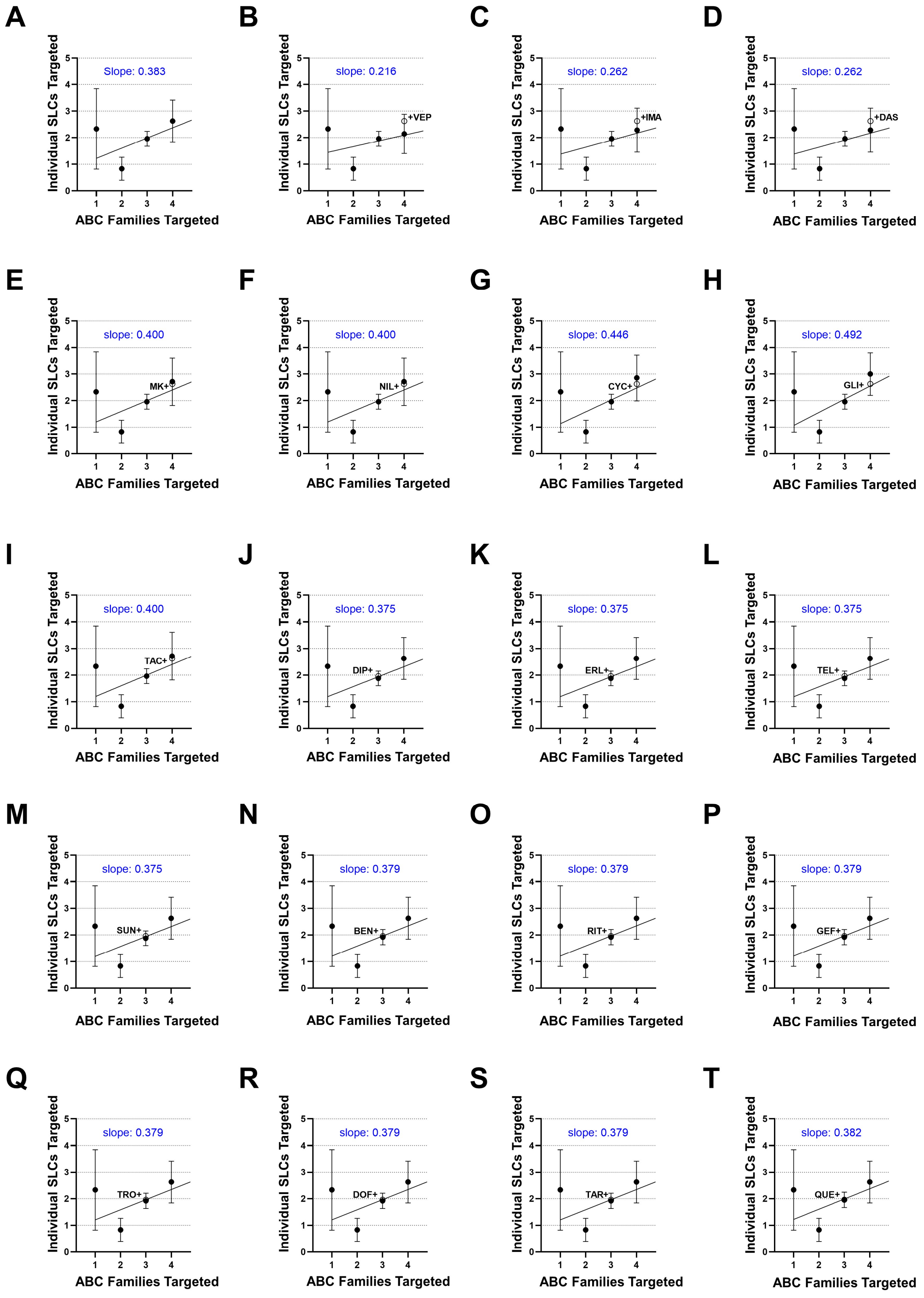

A
Slope: 0.383
Individual SLCs Targeted
ABC Families Targeted
B
slope: 0.216
+VEP
Individual SLCs Targeted
ABC Families Targeted
C
slope: 0.262
+IMA
Individual SLCs Targeted
ABC Families Targeted
D
slope: 0.262
+DAS
Individual SLCs Targeted
ABC Families Targeted
E
slope: 0.400
MK+
Individual SLCs Targeted
ABC Families Targeted
F
slope: 0.400
NIL+
Individual SLCs Targeted
ABC Families Targeted
G
slope: 0.446
CYC+
Individual SLCs Targeted
ABC Families Targeted
H
slope: 0.492
GLI+
Individual SLCs Targeted
ABC Families Targeted
I
slope: 0.400
TAC+
Individual SLCs Targeted
ABC Families Targeted
J
slope: 0.375
DIP+
Individual SLCs Targeted
ABC Families Targeted
K
slope: 0.375
ERL+
Individual SLCs Targeted
ABC Families Targeted
L
slope: 0.375
TEL+
Individual SLCs Targeted
ABC Families Targeted
M
slope: 0.375
SUN+
Individual SLCs Targeted
ABC Families Targeted
N
slope: 0.379
BEN+
Individual SLCs Targeted
ABC Families Targeted
O
slope: 0.379
RIT+
Individual SLCs Targeted
ABC Families Targeted
P
slope: 0.379
GEF+
Individual SLCs Targeted
ABC Families Targeted
Q
slope: 0.379
TRO+
Individual SLCs Targeted
ABC Families Targeted
R
slope: 0.379
DOF+
Individual SLCs Targeted
ABC Families Targeted
S
slope: 0.379
TAR+
Individual SLCs Targeted
ABC Families Targeted
T
slope: 0.382
QUE+
Individual SLCs Targeted
ABC Families Targeted

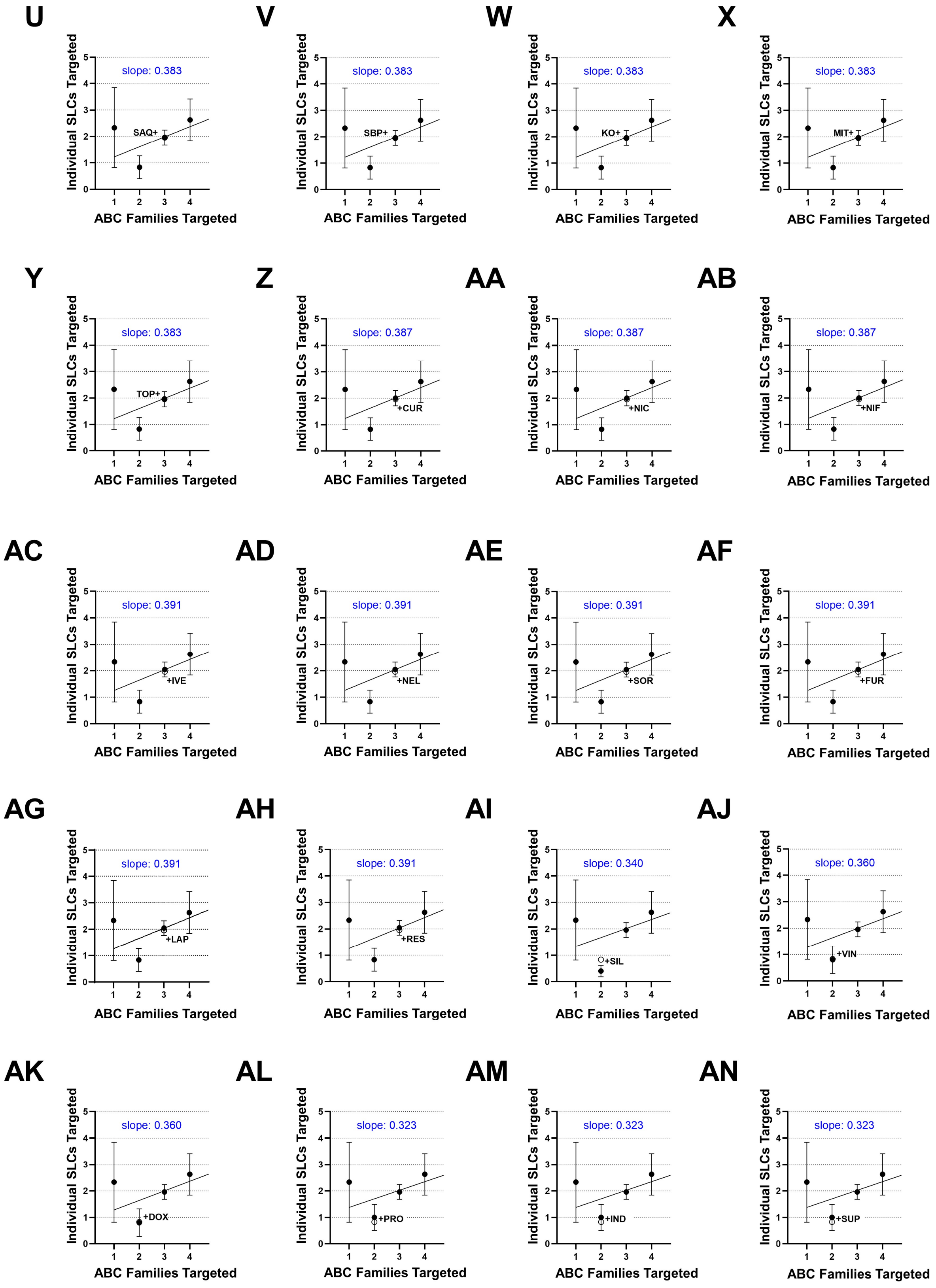
U
slope: 0.383
SAQ+
V
slope: 0.383
SBP+
W
slope: 0.383
KO+
X
slope: 0.383
MIT+
Y
slope: 0.383
TOP+
Z
slope: 0.387
+CUR
AA
slope: 0.387
+NIC
AB
slope: 0.387
+NIF
AC
slope: 0.391
+IVE
AD
slope: 0.391
+NEL
AE
slope: 0.391
+SOR
AF
slope: 0.391
+FUR
AG
slope: 0.391
+LAP
AH
slope: 0.391
+RES
AI
slope: 0.340
+SIL
AJ
slope: 0.360
+VIN
AK
slope: 0.360
+DOX
AL
slope: 0.323
+PRO
AM
slope: 0.323
+IND
AN
slope: 0.323
+SUP
Individual SLCs Targeted
ABC Families Targeted

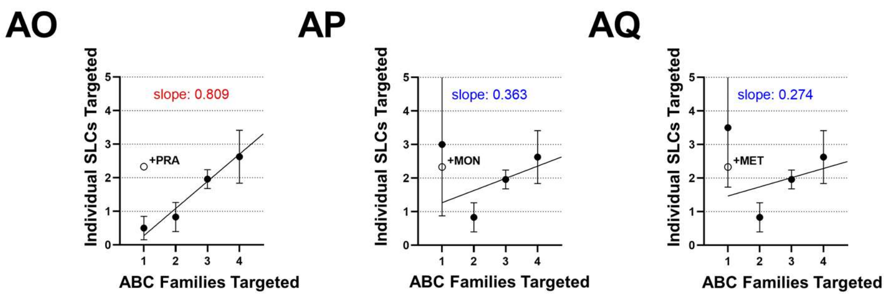

Supplementary Fig. S8. Leave-one-out approach of the correlation between multitargeticity against ABC families and multitargeticity against individual SLCs. The original correlation (black closed circles) is given in **A**. **B**–**AQ** correlation excluding the value for **VEP** (**B**), **IMA** (**C**), **DAS** (**D**), **MK** (**E**), **NIL** (**F**), **CYC** (**G**), **GLI** (**H**), **TAC** (**I**), **DIP** (**J**), **ERL** (**K**), **TEL** (**L**), **SUN** (**M**), **BEN** (**N**), **RIT** (**O**), **GEF** (**P**), **TRO** (**Q**), **DOF** (**R**), **TAR** (**S**), **QUE** (**T**), **SAQ** (**U**), **SBP** (**V**), **KO** (**W**), **MIT** (**X**), **TOP** (**Y**), **CUR** (**Z**), **NIC** (**AA**), **NIF** (**AB**), **IVE** (**AC**), **NEL** (**AD**), **SOR** (**AE**), **FUR** (**AF**), **LAP** (**AG**), **RES** (**AH**), **SIL** (**AI**), **VIN** (**AJ**), **DOX** (**AK**), **PRO** (**AL**), **IND** (**AM**), **SUP** (**AN**), **PRA** (**AO**), **MON** (**AP**), and **MET** (**AQ**). Open circles (**B**–**AQ**): original datapoint with all values included.

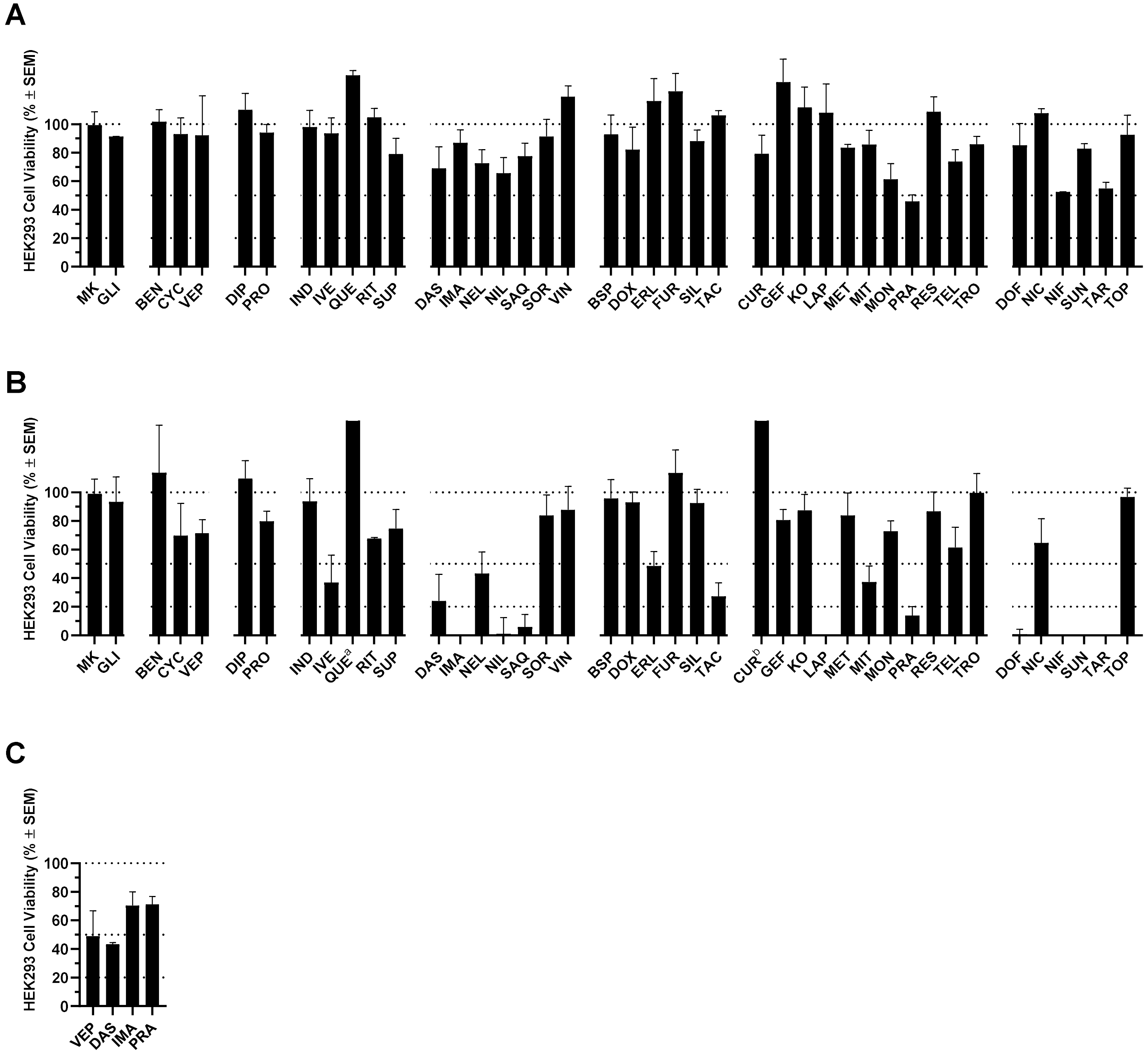

Supplementary Fig. S9. Cell viability screening of the 42 tested pan-ABC transporter modulators in HEK293 cells at 10 µM (**A**) and 100 µM (**B**) after 2 h of exposure; [a] **QUE** increased cell viability to 263% ± 23%; [b] **CUR** increased cell viability to 170% ± 18%. **C** Cell viability screening of the 4 privileged ligands identified in the present study in HEK293 cells at 10 µM after 72 h of exposure. Data shown as mean ± SEM of three independent experiments.

References of Supplementary Table S1

(1) Tsuruoka, S., Ishibashi, K., Yamamoto, H., Wakaumi, M., Suzuki, M., Schwartz, G. J., Imai, M., and Fujimura, A. (2002) Functional analysis of ABCA8, a new drug transporter. Biochem. Biophys. Res. Commun. 298 (1), 41–45. DOI: 10.1016/s0006-291x(02)02389-6

(2) Matsson, P., Pedersen, J. M., Norinder, U., Bergstrom, C. A., and Artursson, P. (2009) Identification of novel specific and general inhibitors of the three major human atp-binding cassette transporters P-gp, BCRP and MRP2 among registered drugs. Pharm. Res. 26 (8), 1816–1831. DOI: 10.1007/s11095-009-9896-0

(3) Bieczynski, F., Burkhardt-Medicke, K., Luquet, C. M., Scholz, S., and Luckenbach, T. (2021) Chemical effects on dye efflux activity in live zebrafish embryos and on zebrafish ABCB4 ATPase activity. FEBS Lett. 595 (6), 828–843. DOI: 10.1002/1873-3468.14015

(4) Pedersen, J. M., Matsson, P., Bergstrom, C. A., Hoogstraate, J., Noren, A., LeCluyse, E. L., and Artursson, P. (2013) Early identification of clinically relevant drug interactions with the human bile salt export pump (BSEP/ABCB11). Toxicol. Sci. 136 (2), 328–343. DOI: 10.1093/toxsci/kft197

(5) Stefan, S. M. and Wiese, M. (2019) Small-molecule inhibitors of multidrug resistance-associated protein 1 and related processes: A historic approach and recent advances. Med. Res. Rev. 39 (1), 176–264. DOI: 10.1002/med.21510

(6) Csandl, M. A., Conseil, G., and Cole, S. P. (2016) Cysteinyl leukotriene receptor 1/2 antagonists nonselectively modulate organic anion transport by

multidrug resistance proteins (MRP1-4). Drug Metab. Dispos. 44 (6), 857–866. DOI: 10.1124/dmd.116.069468

(7) Zhou, S. F., Wang, L. L., Di, Y. M., Xue, C. C., Duan, W., Li, C. G., and Li, Y. (2008) Substrates and inhibitors of human multidrug resistance associated proteins and the implications in drug development. Curr. Med. Chem. 15 (20), 1981–2039. DOI: 10.2174/092986708785132870

(8) Wu, C. P., Calcagno, A. M., Hladky, S. B., Ambudkar, S. V., and Barrand, M. A. (2005) Modulatory effects of plant phenols on human multidrug-resistance proteins 1, 4 and 5 (ABCC1, 4 and 5). FEBS J. 272 (18), 4725–4740. DOI: 10.1111/j.1742-4658.2005.04888.x

(9) Cheung, L., Yu, D. M., Neiron, Z., Failes, T. W., Arndt, G. M., and Fletcher, J. I. (2015) Identification of new MRP4 inhibitors from a library of FDA approved drugs using a high-throughput bioluminescence screen. Biochem. Pharmacol. 93 (3), 380–388. DOI: 10.1016/j.bcp.2014.11.006

(10) Karla, P. K., Quinn, T. L., Herndon, B. L., Thomas, P., Pal, D., and Mitra, A. (2009) Expression of multidrug resistance associated protein 5 (MRP5) on cornea and its role in drug efflux. J. Ocul. Pharmacol. Ther. 25 (2), 121–132. DOI: 10.1089/jop.2008.0084

(11) Borst, P., de Wolf, C., and van de Wetering, K. (2007) Multidrug resistance-associated proteins 3, 4, and 5. Pflugers Arch. 453 (5), 661–673. DOI: 10.1007/s00424-006-0054-9

(12) Zhou, Y., Hopper-Borge, E., Shen, T., Huang, X. C., Shi, Z., Kuang, Y. H., Furukawa, T., Akiyama, S., Peng, X. X., Ashby, C. R., Jr., Chen, X., Kruh, G. D., and Chen, Z. S. (2009) Cepharanthine is a potent reversal agent for

MRP7(ABCC10)-mediated multidrug resistance. Biochem. Pharmacol. 77 (6), 993–1001. DOI: 10.1016/j.bcp.2008.12.005

(13) Tun-Yhong, W., Chinpaisal, C., Pamonsinlapatham, P., and Kaewkitichai, S. (2017) Tenofovir disoproxil fumarate is a new substrate of ATP-binding cassette subfamily C member 11. Antimicrob. Agents Chemother. 61 (4), DOI: 10.1128/AAC.01725-16

(14) Pahnke, J., Bascunana, P., Brackhan, M., Stefan, K., Namasivayam, V., Koldamova, R., Wu, J., Möhle, L., and Stefan, S. M. (2021) Strategies to gain novel alzheimer's disease diagnostics and therapeutics using modulators of ABCA transporters. Free Neuropathol. 2, 33. DOI: 10.17879/freeneuropathology-2021-3528

(15) Horikawa, M., Kato, Y., Tyson, C. A., and Sugiyama, Y. (2003) Potential cholestatic activity of various therapeutic agents assessed by bile canalicular membrane vesicles isolated from rats and humans. Drug Metab. Pharmacokinet. 18 (1), 16–22. DOI: 10.2133/dmpk.18.16

(16) de Jonge, H. R., Ardelean, M. C., Bijvelds, M. J. C., and Vergani, P. (2020) Strategies for cystic fibrosis transmembrane conductance regulator inhibition: From molecular mechanisms to treatment for secretory diarrhoeas. FEBS Lett. 594 (23), 4085–4108. DOI: 10.1002/1873-3468.13971

(17) Lim, J. G., Lee, H. Y., Yun, J. E., Kim, S. P., Park, J. W., Suh, S. I., Jang, B. C., Cho, C. H., Bae, J. H., Kim, S. S., Han, J., Park, M. J., and Song, D. K. (2004) Taurine block of cloned ATP-sensitive K+ channels with different sulfonylurea receptor subunits expressed in xenopus laevis oocytes. Biochem. Pharmacol. 68 (5), 901–910. DOI: 10.1016/j.bcp.2004.05.050

(18) Ivnitski-Steele, I., Larson, R. S., Lovato, D. M., Khawaja, H. M., Winter, S. S., Oprea, T. I., Sklar, L. A., and Edwards, B. S. (2008) High-throughput flow cytometry to detect selective inhibitors of ABCB1, ABCC1, and ABCG2 transporters. Assay Drug Dev. Technol. 6 (2), 263–276. DOI: 10.1089/adt.2007.107

(19) Zelcer, N., Saeki, T., Reid, G., Beijnen, J. H., and Borst, P. (2001) Characterization of drug transport by the human multidrug resistance protein 3 (ABCC3). J. Biol. Chem. 276 (49), 46400–46407. DOI: 10.1074/jbc.M107041200

(20) El-Sheikh, A. A., van den Heuvel, J. J., Koenderink, J. B., and Russel, F. G. (2008) Effect of hypouricaemic and hyperuricaemic drugs on the renal urate efflux transporter, multidrug resistance protein 4. Br. J. Pharmacol. 155 (7), 1066–1075. DOI: 10.1038/bjp.2008.343

(21) Bai, J., Lai, L., Yeo, H. C., Goh, B. C., and Tan, T. M. (2004) Multidrug resistance protein 4 (MRP4/ABCC4) mediates efflux of bimane-glutathione. Int. J. Biochem. Cell. Biol. 36 (2), 247–257. DOI: 10.1016/s1357-2725(03)00236-x

(22) Stefan, S. M., Jansson, P. J., Pahnke, J., and Namasivayam, V. (2022) A curated binary pattern multitarget dataset of focused ATP-binding cassette transporter inhibitors. Sci. Data 9 (1), 446. DOI: 10.1038/s41597-022-01506-z

(23) Williamson, G., Aeberli, I., Miguet, L., Zhang, Z., Sanchez, M. B., Crespy, V., Barron, D., Needs, P., Kroon, P. A., Glavinas, H., Krajcsi, P., and Grigorov, M. (2007) Interaction of positional isomers of quercetin glucuronides with the transporter ABCC2 (cMOAT, MRP2). Drug Metab. Dispos. 35 (8), 1262–1268. DOI: 10.1124/dmd.106.014241

(24) Cserepes, J., Szentpetery, Z., Seres, L., Ozvegy-Laczka, C., Langmann, T., Schmitz, G., Glavinas, H., Klein, I., Homolya, L., Varadi, A., Sarkadi, B., and Elkind, N. B. (2004) Functional expression and characterization of the human ABCG1 and ABCG4 proteins: Indications for heterodimerization. Biochem. Biophys. Res. Commun. 320 (3), 860–867. 10.1016/j.bbrc.2004.06.037: 10.1016/j.bbrc.2004.06.037

(25) Saeed, M. E. M., Boulos, J. C., Elhaboub, G., Rigano, D., Saab, A., Loizzo, M. R., Hassan, L. E. A., Sugimoto, Y., Piacente, S., Tundis, R., Yagi, S., Khalid, H., and Efferth, T. (2019) Cytotoxicity of cucurbitacin e from citrullus colocynthis against multidrug-resistant cancer cells. Phytomedicine 62, DOI: 10.1016/j.phymed.2019.152945

(26) Wielinga, P. R., van der Heijden, I., Reid, G., Beijnen, J. H., Wijnholds, J., and Borst, P. (2003) Characterization of the MRP4- and MRP5-mediated transport of cyclic nucleotides from intact cells. J. Biol. Chem. 278 (20), 17664–17671. DOI: 10.1074/jbc.M212723200

(27) Eadie, L. N., Dang, P., Goyne, J. M., Hughes, T. P., and White, D. L. (2018) ABCC6 plays a significant role in the transport of nilotinib and dasatinib, and contributes to tki resistance in vitro, in both cell lines and primary patient mononuclear cells. PLoS One 13 (1), e0192180. DOI: 10.1371/journal.pone.0192180

(28) Ambrus, C., Bakos, E., Sarkadi, B., Özvegy-Laczka, C., and Telbisz, A. (2021) Interactions of anti-COVID-19 drug candidates with hepatic transporters may cause liver toxicity and affect pharmacokinetics. Sci. Rep. 11 (1), 17810. DOI: 10.1038/s41598-021-97160-3

(29) Telbisz, A., Ambrus, C., Mozner, O., Szabo, E., Varady, G., Bakos, E., Sarkadi, B., and Özvegy-Laczka, C. (2021) Interactions of potential anti-COVID-19 compounds with multispecific ABC and OATP drug transporters. Pharmaceutics 13 (1), 81. DOI: 10.3390/pharmaceutics13010081

(30) Singh, K., Patil, R. B., Patel, V., Remenyik, J., Hegedus, T., and Goda, K. (2023) Synergistic inhibitory effect of quercetin and cyanidin-3O-sophoroside on ABCB1. Int. J. Mol. Sci. 24 (14), DOI: 10.3390/ijms241411341

(31) Mohos, V., Fliszar-Nyul, E., Ungvari, O., Kuffa, K., Needs, P. W., Kroon, P. A., Telbisz, A., Özvegy-Laczka, C., and Poor, M. (2020) Inhibitory effects of quercetin and its main methyl, sulfate, and glucuronic acid conjugates on cytochrome P450 enzymes, and on OATP, BCRP and MRP2 transporters. Nutrients 12 (8), 2306. DOI: 10.3390/nu12082306

(32) Saito, H., Toyoda, Y., Hirata, H., Ota-Kontani, A., Tsuchiya, Y., Takada, T., and Suzuki, H. (2020) Soy isoflavone genistein inhibits an axillary osmidrosis risk factor ABCC11: In vitro screening and fractional approach for ABCC11-inhibitory activities in plant extracts and dietary flavonoids. Nutrients 12 (8), 2452. DOI: 10.3390/nu12082452

(33) Fukuda, Y., Takenaka, K., Sparreboom, A., Cheepala, S. B., Wu, C. P., Ekins, S., Ambudkar, S. V., and Schuetz, J. D. (2013) Human immunodeficiency virus protease inhibitors interact with ATP binding cassette transporter 4/multidrug resistance protein 4: A basis for unanticipated enhanced cytotoxicity. Mol. Pharmacol. 84 (3), 361–371. DOI: 10.1124/mol.113.086967

(34) Gupta, A., Zhang, Y., Unadkat, J. D., and Mao, Q. (2004) HIV protease inhibitors are inhibitors but not substrates of the human breast cancer

resistance protein (BCRP/ABCG2). J. Pharmacol. Exp. Ther. 310 (1), 334–DOI: 10.1124/jpet.104.065342

(35) Bessho, Y., Oguri, T., Ozasa, H., Uemura, T., Sakamoto, H., Miyazaki, M., Maeno, K., Sato, S., and Ueda, R. (2009) ABCC10/MRP7 is associated with vinorelbine resistance in non-small cell lung cancer. Oncol. Rep. 21 (1), 263–268.

(36) Hupfeld, T., Chapuy, B., Schrader, V., Beutler, M., Veltkamp, C., Koch, R., Cameron, S., Aung, T., Haase, D., Larosee, P., Truemper, L., and Wulf, G. G. (2013) Tyrosinekinase inhibition facilitates cooperation of transcription factor SALL4 and ABC transporter A3 towards intrinsic CML cell drug resistance. Br. J. Haematol. 161 (2), 204–213. DOI: 10.1111/bjh.12246

(37) Beretta, G. L., Cassinelli, G., Pennati, M., Zuco, V., and Gatti, L. (2017) Overcoming ABC transporter-mediated multidrug resistance: The dual role of tyrosine kinase inhibitors as multitargeting agents. Eur. J. Med. Chem. 142, 271–289. DOI: 10.1016/j.ejmech.2017.07.062

(38) Furmanski, B. D., Hu, S., Fujita, K. I., Li, L., Gibson, A. A., Janke, L. J., Williams, R. T., Schuetz, J. D., Sparreboom, A., and Baker, S. D. (2013) Contribution of ABCC4-mediated gastric transport to the absorption and efficacy of dasatinib. Clin. Cancer Res. 19 (16), 4359–4370. DOI: 10.1158/1078-0432.CCR-13-0980

(39) Malofeeva, E. V., Domanitskaya, N., Gudima, M., and Hopper-Borge, E. A. (2012) Modulation of the ATPase and transport activities of broad-acting multidrug resistance factor ABCC10 (MRP7). Cancer Res. 72 (24), 6457–6467. DOI: 10.1158/0008-5472.CAN-12-1340

(40) Pick, A. and Wiese, M. (2012) Tyrosine kinase inhibitors influence ABCG2 expression in EGFR-positive MDCK BCRP cells via the PI3K/AKT signaling pathway. ChemMedChem 7 (4), 650–662. DOI: 10.1002/cmdc.201100543

(41) Trojani, A., Pungolino, E., Dal Molin, A., Lodola, M., Rossi, G., D'Adda, M., Perego, A., Elena, C., Turrini, M., Borin, L., Bucelli, C., Malato, S., Carraro, M. C., Spina, F., Latargia, M. L., Artale, S., Spedini, P., Anghilieri, M., Di Camillo, B., Baruzzo, G., De Canal, G., Iurlo, A., Morra, E., and Cairoli, R. (2019) Nilotinib interferes with cell cycle, ABC transporters and JAK-STAT signaling pathway in CD34+/lin- cells of patients with chronic phase chronic myeloid leukemia after 12 months of treatment. PLoS One 14 (7), e0218444. DOI: 10.1371/journal.pone.0218444

(42) Puri, S., Stefan, K., Khan, S. L., Pahnke, J., Stefan, S. M., and Juvale, K. (2023) Indole derivatives as new structural class of potent and antiproliferative inhibitors of monocarboxylate transporter 1 (MCT1; SLC16A1). J. Med. Chem. 66 (1), 657–676. DOI: 10.1021/acs.jmedchem.2c01612

(43) Peng, X. X., Shi, Z., Damaraju, V. L., Huang, X. C., Kruh, G. D., Wu, H. C., Zhou, Y., Tiwari, A., Fu, L., Cass, C. E., and Chen, Z. S. (2008) Up-regulation of MRP4 and down-regulation of influx transporters in human leukemic cells with acquired resistance to 6-mercaptopurine. Leuk. Res. 32 (5), 799–809. DOI: 10.1016/j.leukres.2007.09.015

(44) Chen, Z. S., Hopper-Borge, E., Belinsky, M. G., Shchaveleva, I., Kotova, E., and Kruh, G. D. (2003) Characterization of the transport properties of human multidrug resistance protein 7 (MRP7, ABCC10). Mol. Pharmacol. 63 (2), 351–358. DOI: 10.1124/mol.63.2.351

(45) Al-Mohizea, A. M. (2010) Influence of intestinal efflux pumps on the absorption and transport of furosemide. Saudi Pharm. J. 18 (2), 97–101. DOI: 10.1016/j.jsps.2010.02.005

(46) Chapa, R., Li, C. Y., Basit, A., Thakur, A., Ladumor, M. K., Sharma, S., Singh, S., Selen, A., and Prasad, B. (2020) Contribution of uptake and efflux transporters to oral pharmacokinetics of furosemide. ACS Omega 5 (51), 32939–32950. DOI: 10.1021/acsomega.0c03930

(47) Zhang, S. and Morris, M. E. (2003) Effects of the flavonoids biochanin A, morin, phloretin, and silymarin on P-glycoprotein-mediated transport. J. Pharmacol. Exp. Ther. 304 (3), 1258–1267. DOI: 10.1124/jpet.102.044412

(48) Suzuki, K., Saito, K., Tsujimura, S., Nakayamada, S., Yamaoka, K., Sawamukai, N., Iwata, S., Nawata, M., Nakano, K., and Tanaka, Y. (2010) Tacrolimus, a calcineurin inhibitor, overcomes treatment unresponsiveness mediated by P-glycoprotein on lymphocytes in refractory rheumatoid arthritis. J. Rheumatol. 37 (3), 512–520. DOI: 10.3899/jrheum.090048

(49) Ritschel, T., Hermans, S. M., Schreurs, M., van den Heuvel, J. J., Koenderink, J. B., Greupink, R., and Russel, F. G. (2014) In silico identification and in vitro validation of potential cholestatic compounds through 3D ligand-based pharmacophore modeling of BSEP inhibitors. Chem. Res. Toxicol. 27 (5), 873– DOI: 10.1021/tx5000393

(50) Chearwae, W., Shukla, S., Limtrakul, P., and Ambudkar, S. V. (2006) Modulation of the function of the multidrug resistance-linked ATP-binding cassette transporter ABCG2 by the cancer chemopreventive agent curcumin. Mol. Cancer Ther. 5 (8), 1995–2006. DOI: 10.1158/1535-7163.MCT-06-0087

(51) Li, Y., Revalde, J. L., Reid, G., and Paxton, J. W. (2011) Modulatory effects of curcumin on multi-drug resistance-associated protein 5 in pancreatic cancer cells. Cancer. Chemother. Pharmacol. 68 (3), 603–610. DOI: 10.1007/s00280-010-1515-6

(52) Pratt, S., Shepard, R. L., Kandasamy, R. A., Johnston, P. A., Perry, W., and Dantzig, A. H. (2005) The multidrug resistance protein 5 (ABCC5) confers resistance to 5-fluorouracil and transports its monophosphorylated metabolites. Mol. Cancer. Ther. 4 (5), 855–863. DOI: 10.1158/1535-7163.MCT-04-0291

(53) Zhang, S., Yang, X., and Morris, M. E. (2004) Combined effects of multiple flavonoids on breast cancer resistance protein (ABCG2)-mediated transport. Pharm. Res. 21 (7), 1263–1273. DOI: 10.1023/b:pham.0000033015.84146.4c

(54) Wiese, M. and Stefan, S. M. (2019) The A-B-C of small-molecule ABC transport protein modulators: From inhibition to activation – A case study of multidrug resistance-associated protein 1 (ABCC1). Med. Res. Rev. 39 (6), 2031–2081. DOI: 10.1002/med.21573

(55) Yaguchi, T., and Onishi, T. (2018) Estrogen induces cell proliferation by promoting abcg2-mediated efflux in endometrial cancer cells. Biochem Biophys Rep 16, 74-78. 10.1016/j.bbrep.2018.10.005: 10.1016/j.bbrep.2018.10.005

(56) Weiss, J., Sauer, A., Divac, N., Herzog, M., Schwedhelm, E., Boger, R. H., Haefeli, W. E., and Benndorf, R. A. (2010) Interaction of angiotensin receptor type 1 blockers with ATP-binding cassette transporters. Biopharm. Drug Dispos. 31 (2–3), 150–161. DOI: 10.1002/bdd.699

(57) Shubbar, M. H., and Penny, J. I. (2020) Therapeutic drugs modulate ATP-binding cassette transporter-mediated transport of amyloid beta$_{(1-42)}$ in brain microvascular endothelial cells. Eur. J. Pharmacol. 874, 173009. DOI: 10.1016/j.ejphar.2020.173009

(58) Weiss, J., Sauer, A., Herzog, M., Boger, R. H., Haefeli, W. E., and Benndorf, R. A. (2009) Interaction of thiazolidinediones (glitazones) with the ATP-binding cassette transporters P-glycoprotein and breast cancer resistance protein. Pharmacology 84 (5), 264–270. DOI: 10.1159/000241734

(59) Marroquin, L. D., Bonin, P. D., Keefer, J., and Schroeter, T. (2017) Assessment of bile salt export pump (BSEP) inhibition in membrane vesicles using radioactive and LC/MS-based detection methods. Curr. Protoc. Toxicol. 71, 14 DOI: 10.1002/cptx.15

(60) Yang, K., Pfeifer, N. D., Kock, K., and Brouwer, K. L. (2015) Species differences in hepatobiliary disposition of taurocholic acid in human and rat sandwich-cultured hepatocytes: Implications for drug-induced liver injury. J. Pharmacol. Exp. Ther. 353 (2), 415–423. DOI: 10.1124/jpet.114.221564

(61) Katayama, R., Koike, S., Sato, S., Sugimoto, Y., Tsuruo, T., and Fujita, N. (2009) Dofequidar fumarate sensitizes cancer stem-like side population cells to chemotherapeutic drugs by inhibiting ABCG2/BCRP-mediated drug export. Cancer Sci. 100 (11), 2060–2068. DOI: 10.1111/j.1349-7006.2009.01288.x

(62) Zhang, Y., Gupta, A., Wang, H., Zhou, L., Vethanayagam, R. R., Unadkat, J. D., and Mao, Q. (2005) BCRP transports dipyridamole and is inhibited by calcium channel blockers. Pharm. Res. 22 (12), 2023–2034. DOI: 10.1007/s11095-005-8384-4

(63) Shukla, S., Robey, R. W., Bates, S. E., and Ambudkar, S. V. (2009) Sunitinib (Sutent, SU11248), a small-molecule receptor tyrosine kinase inhibitor, blocks function of the ATP-binding cassette (ABC) transporters P-glycoprotein (ABCB1) and ABCG2. Drug Metab. Dispos. 37 (2), 359–365. DOI: 10.1124/dmd.108.024612

(64) Hu, S., Chen, Z., Franke, R., Orwick, S., Zhao, M., Rudek, M. A., Sparreboom, A., and Baker, S. D. (2009) Interaction of the multikinase inhibitors sorafenib and sunitinib with solute carriers and ATP-binding cassette transporters. Clin. Cancer Res. 15 (19), 6062–6069. DOI: 10.1158/1078-0432.CCR-09-0048

(65) Kühnle, M., Egger, M., Müller, C., Mahringer, A., Bernhardt, G., Fricker, G., König, B., and Buschauer, A. (2009) Potent and selective inhibitors of breast cancer resistance protein (ABCG2) derived from the p-glycoprotein (ABCB1) modulator tariquidar. J. Med. Chem. 52 (4), 1190–1197. DOI: 10.1021/jm8013822

(66) Kathawala, R. J., Wang, Y. J., Ashby, C. R., Jr., and Chen, Z. S. (2014) Recent advances regarding the role of ABC subfamily C member 10 (ABCC10) in the efflux of antitumor drugs. Chin. J. Cancer 33 (5), 223–230. DOI: 10.5732/cjc.013.10122

(67) Poller, B., Wagenaar, E., Tang, S. C., and Schinkel, A. H. (2011) Double-transduced MDCKII cells to study human P-glycoprotein (ABCB1) and breast cancer resistance protein (ABCG2) interplay in drug transport across the blood-brain barrier. Mol. Pharm. 8 (2), 571–582. DOI: 10.1021/mp1003898

(68) Leggas, M., Adachi, M., Scheffer, G. L., Sun, D., Wielinga, P., Du, G., Mercer, K. E., Zhuang, Y., Panetta, J. C., Johnston, B., Scheper, R. J., Stewart, C. F., and Schuetz, J. D. (2004) MRP4 confers resistance to topotecan and protects

the brain from chemotherapy. Mol. Cell Biol. 24 (17), 7612–7621. DOI: 10.1128/MCB.24.17.7612-7621.2004
